# Regio- and stereospecific rhodium-catalyzed allylic alkylation with an acyl anion equivalent: an approach to acyclic α-ternary β,γ-unsaturated aryl ketones†
†Electronic supplementary information (ESI) available. See DOI: 10.1039/c6sc05705e



**DOI:** 10.1039/c6sc05705e

**Published:** 2017-03-31

**Authors:** Ben W. H. Turnbull, Jungha Chae, Samuel Oliver, P. Andrew Evans

**Affiliations:** a Department of Chemistry , Queen's University , 90 Bader Lane , Kingston , K7L 3N6 , Ontario , Canada . Email: Andrew.Evans@chem.queensu.ca; b Department of Chemistry , The University of Liverpool , Crown Street , Liverpool , L69 7ZD , UK

## Abstract

The regio- and stereospecific rhodium-catalyzed allylic alkylation of secondary allylic carbonates with cyanohydrin pronucleophiles facilitates the direct construction of acyclic α-ternary β,γ-unsaturated aryl ketones. Interestingly, this study illustrates the impact of deaggregating agents on regiocontrol and the electronic nature of the aryl component to suppress olefin isomerization. In addition, we demonstrate that the dimethylamino substituent, which modulates the p*K*_a_ of the α-ternary β,γ-unsaturated aryl ketone, provides a useful synthetic handle for further functionalization *via* Kumada cross-coupling of the aryl trimethylammonium salt. Finally, the stereospecific alkylation of a chiral nonracemic secondary allylic carbonate affords the enantioenriched α-ternary aryl ketone, which was employed in a formal synthesis of trichostatic acid to illustrate that the allylic alkylation proceeds with net retention of configuration.

## Abstract 2

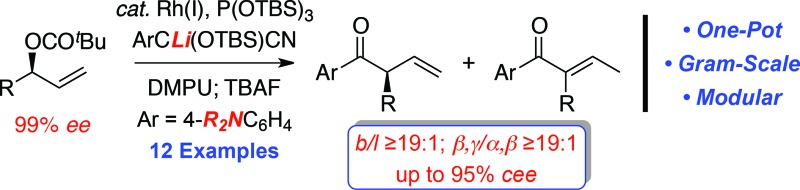
The regio- and stereospecific rhodium-catalyzed allylic alkylation of secondary allylic carbonates with cyanohydrin pronucleophiles facilitates the direct construction of acyclic α-ternary β,γ-unsaturated aryl ketones.

## Introduction

**Scheme 1:**
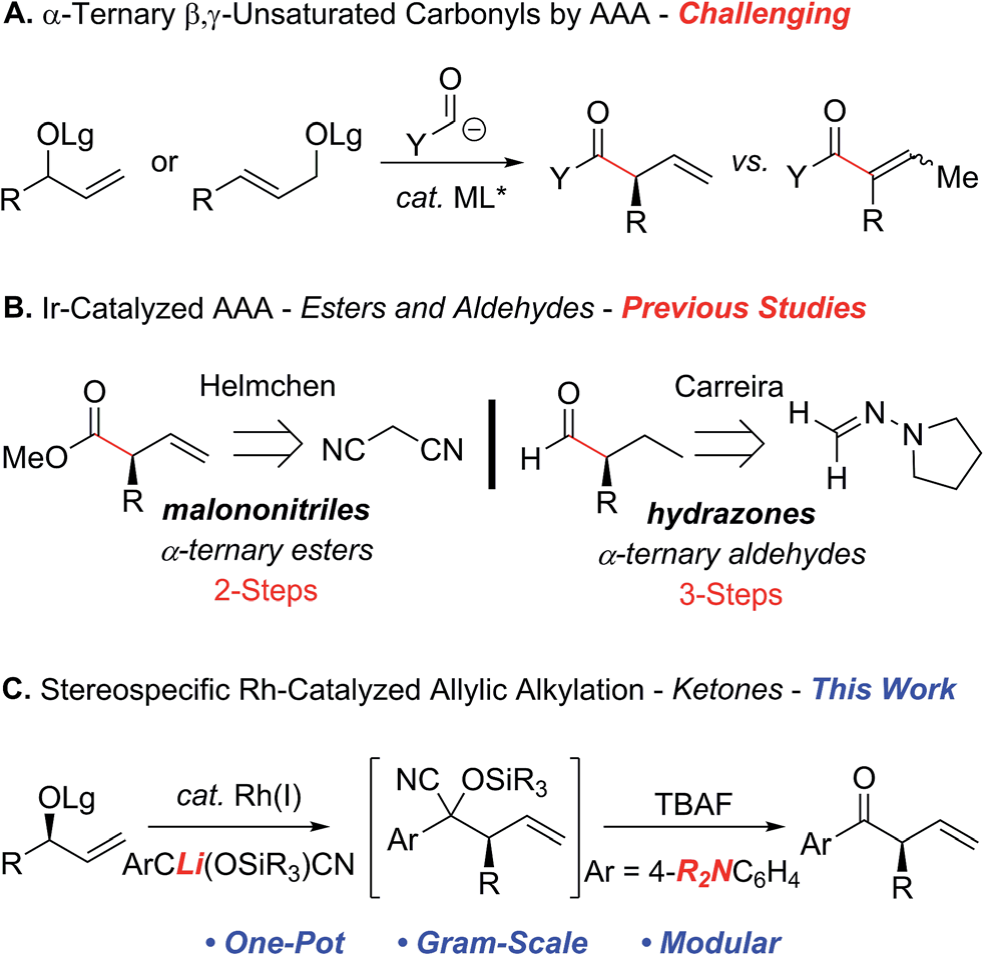
Factors affecting the development of the rhodium-catalyzed allylic alkylation with an acyl anion equivalent.

The ability to reverse the natural polarity of a functional group using an *umpolung* synthon can often provide a strategic advantage by circumventing the underlying limitations associated with a more conventional approach.^1^ For example, the combination of acyl anion equivalents with the asymmetric transition metal-catalyzed allylic alkylation reaction (AAA)^2–5^ provides an attractive strategy for the construction of acyclic α-ternary β,γ-unsaturated carbonyl derivatives (Scheme 1A). Although this approach avoids some of the challenges associated with asymmetric enolate alkylation, namely, polyalkylation, epimerization and electrophile scope,^6–8^ the reaction requires the construction of an intermediate that can either suppress the isomerization of the olefin to the thermodynamically more stable α,β-unsaturated derivative or requires the removal of the olefin prior to the unveiling of the carbonyl motif.^9^ In addition, many of the acyl anion equivalents that have been successfully deployed in the asymmetric transition metal-catalyzed allylic alkylation reaction require additional functionalization steps to reveal the carbonyl moiety at the desired oxidation state, which detracts from the overall efficiency and utility of this approach (Scheme 1B). For example, Helmchen *et al.* reported malononitrile as a methoxycarbonyl anion surrogate in the asymmetric iridium-catalyzed allylic substitution reaction, which requires selective oxidation and functionalization to afford the corresponding methyl ester.^5*d*^ In addition, Breitler and Carreira demonstrated the utility of *N*,*N*-dialkylhydrazones as a formyl anion equivalent, albeit the acidic conditions required to provide the α-ternary aldehyde necessitate prior hydrogenation of the alkene to avoid isomerization.^5*f*^

In a program directed toward the development of rhodium-catalyzed allylic substitution reactions,^10,11^ we recently reported a highly regio- and stereospecific reaction of chiral nonracemic *tertiary* allylic alcohol derivatives with an acyl anion equivalent, which provides a convenient approach to quaternary stereogenic centers.^12^ For instance, *tert*-butyldimethylsilyl-protected cyanohydrins derived from the corresponding aryl and alkenyl aldehydes,^13^ function as acyl anion equivalents that can be unmasked *in situ* to afford the requisite ketone. Hence, we envisioned that the application of this strategy to acyclic chiral nonracemic *secondary* allylic carbonates should provide a direct one-pot approach to enantiomerically enriched α-ternary ketones, provided the isomerization of the olefin could be suppressed.^14,15^ To this end, we envisioned that the electronic nature of the aryl ketones could be tailored to modulate olefin isomerization and permit further functionalization. Herein, we now describe the first rhodium-catalyzed allylic alkylation of secondary allylic carbonates with cyanohydrin pronucleophiles to facilitate the construction of acyclic α-ternary β,γ-unsaturated aryl ketones (Scheme 1C).

## Results and discussion

In accord with our supposition, preliminary studies demonstrated that the aryl ketones were indeed prone to facile isomerization. For example, treatment of the secondary allylic carbonate **1a′** (Lg = CO_2_Me) with the lithium anion of the aryl cyanohydrin **2a′** in the presence of [Rh(COD)Cl]_2_ *modified* with triphenyl phosphite at –10 °C for *ca.* 16 hours, followed by the addition of tetra-*n*-butylammonium fluoride (TBAF) at –40 °C^16^ furnished the trisubstituted enone **5a′** in 41% yield with poor regioselectivity, albeit as a single geometrical isomer (*E*/*Z* ≥19 : 1) (Table 1, entry 1).^17^ Hence, we reasoned that the isomerization could be suppressed by employing a more electron-rich aryl cyanohydrin to modulate the p*K*_a_ of the α-proton in the ketone product. Interestingly, while the 4-methoxy-substituted aryl cyanohydrin **2aa′** afforded less of the trisubstituted α,β-unsaturated ketone (entry 2), the 4-dimethylamino derivative **2a** reverses the outcome to afford the α-ternary β,γ-unsaturated ketone **3a** (entry 3) in good yield and with modest regiocontrol. In an effort to further improve the efficiency and selectivity of this process, we elected to examine the effect of the leaving group, which can often have a dramatic impact on the outcome of metal-catalyzed allylic substitution reactions.^10^ To this end, the more bulky *tert*-butyl carbonate **1a** (Lg = CO_2_^*t*^Bu) improves the formation of **3a** whilst maintaining similar regioselectivity (entry 4). Additional studies probed the effect of the phosphite ligand on regioselectivity, in which tris(*tert*-butyldimethylsilyl) phosphite provided further improvement in regiocontrol (entry 5). Finally, we envisioned the addition of an additive to coordinate the alkali metal would deaggregate the nucleophile and thereby increase the rate of allylic alkylation to provide improved selectivity. Gratifyingly, in accord with this hypothesis, the addition of DMPU provided significant improvement in regioselectivity to afford the α-ternary β,γ-unsaturated aryl ketone **3a** in 89% yield and with ≥19 : 1 selectivity (entry 6).

**Table 1:** Optimization of the regioselective rhodium-catalyzed allylic alkylation of secondary allylic carbonates with an aryl cyanohydrin pronucleophile^*a*^

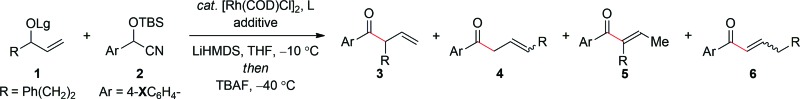								
Entry	**1** where Lg =		**2** where X =		Phosphite L	*b*/*l* ^*b*^ (**3** + **5**) : (**4** + **6**)	**3** : **5** ^*c*^ ^,^ ^*d*^	Yield of **3**/**5** ^*e*^ (%)
1	CO_2_Me	**a′**	H	**a′**	P(OPh)_3_	2 : 1	≤1 : 19	41
2	′′	′′	OMe	**aa′**	′′	3 : 1	1 : 3	62
3	′′	′′	NMe_2_	**a**	′′	5 : 1	8 : 1	59
4	CO_2_^*t*^Bu	**a**	′′	′′	′′	5 : 1	13 : 1	72
5	′′	′′	′′	′′	P(OTBS)_3_	10 : 1	≥19 : 1	80
**6** ^*f*^	**CO**_**2**_^***t***^ **Bu**	′′	**NMe**_**2**_	**a**	**P(OTBS)**_**3**_	**≥19** **:** **1**	**≥19** **:** **1**	**89**

^*a*^All reactions were performed on a 0.25 mmol scale using 2.5 mol% [Rh(COD)Cl]_2_, 10 mol% L, 1.3 equiv. **2** and 1.8 equiv. LiHMDS in THF (2.5 mL) at –10 °C for *ca.* 16 hours, followed by the addition of 4.0 equiv. TBAF at –40 °C.

^*b*^Regioselectivity was determined by 500 MHz ^1^H NMR analysis of the reaction mixtures before deprotection of the cyanohydrin adducts.

^*c*^The ratio of **3** : **5** was determined by 500 MHz ^1^H NMR analysis of the crude ketones.

^*d*^Geometrical selectivity (*E*/*Z* ≥ 19 : 1) was determined by 500 MHz ^1^H NMR analysis of the crude enones.

^*e*^Isolated yields of the branched regioisomer.

^*f*^Reaction was conducted in THF (2.25 mL) and DMPU (0.25 mL).

Table 2 summarizes the application of the optimized reaction conditions (Table 1, entry 6) to the synthesis of a range of α-ternary β,γ-unsaturated aryl ketones **3** using electron-rich aryl cyanohydrins. The reaction is tolerant of a number of important substituents within the allylic carbonate moiety. For example, in addition to the phenethyl derivative, a benzyl group (entries 1–2), long and short alkyl chains (entries 3–4), including branched alkyl chains (entries 5–6) afford the corresponding aryl ketones **3a–f** in excellent yield and selectivity. Additionally, the ability to employ a range of functionalized groups (entries 7–9) is also particularly noteworthy given the problems often associated with these types of electrophiles in conventional enolate alkylation reactions. Finally, a number of related electron-rich aryl cyanohydrins were examined as pronucleophiles for this process, namely, those containing pyrrolidine, piperidine and morpholine substituents, which also facilitate the allylic alkylation without isomerization to the trisubstituted α,β-unsaturated aryl ketone (entries 10–12). Hence, this process affords a great deal of versatility, despite the necessity to utilize a 4-dialkylamino-substituted cyanohydrin to suppress olefin isomerization.

**Table 2:** Scope of the rhodium-catalyzed allylic alkylation to form α-ternary aryl ketones^*a*^

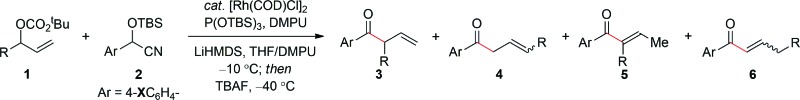						
Entry	**1** R =	**2** X =	*b*/*l* ^*b*^ (**3** + **5**) : (**4** + **6**)		**3** : **5** ^*c*^	Yield of **3** ^*d*^ (%)
1	Ph(CH_2_)_2_	NMe_2_	**a**	≥19 : 1	≥19 : 1	89
2	PhCH_2_	′′	**b**	≥19 : 1	≥19 : 1	83
3	Pr	′′	**c**	≥19 : 1	≥19 : 1	86
4	Me	′′	**d**	≥19 : 1	≥19 : 1	81
5	^*i*^Bu	′′	**e**	≥19 : 1	≥19 : 1	73
6	^*i*^Pen	′′	**f**	≥19 : 1	≥19 : 1	74
7	CH_2_ =CH(CH_2_)_2_	′′	**g**	≥19 : 1	≥19 : 1	71
8	BnOCH_2_	′′	**h**	≥19 : 1	≥19 : 1	71
9	BnO(CH_2_)_2_	′′	**i**	≥19 : 1	≥19 : 1	74
10	Ph(CH_2_)_2_	N(CH_2_)_4_	**j**	≥19 : 1	≥19 : 1	76
11	Ph(CH_2_)_2_	N(CH_2_)_5_	**k**	≥19 : 1	≥19 : 1	70
12	Ph(CH_2_)_2_	N[(CH_2_)]_2_O	**l**	≥19 : 1	≥19 : 1	67

^*a*^All reactions were performed on a 0.5 mmol reaction scale using 2.5 mol% [Rh(COD)Cl]_2_, 10 mol% P(OTBS)_3_, 1.3 equiv. **2** and 1.8 equiv. LiHMDS in THF (4.5 mL) and DMPU (0.5 mL) at –10 °C for *ca.* 16 hours, followed by the addition of 4.0 equiv. TBAF at –40 °C.

^*b*^Regioselectivity was determined by 500 MHz ^1^H NMR analysis of the isolated ketones.

^*c*^The ratio of **3** : **5** was determined by 500 MHz ^1^H NMR analysis of the isolated ketones.

^*d*^Isolated yields.

**Scheme 2:**
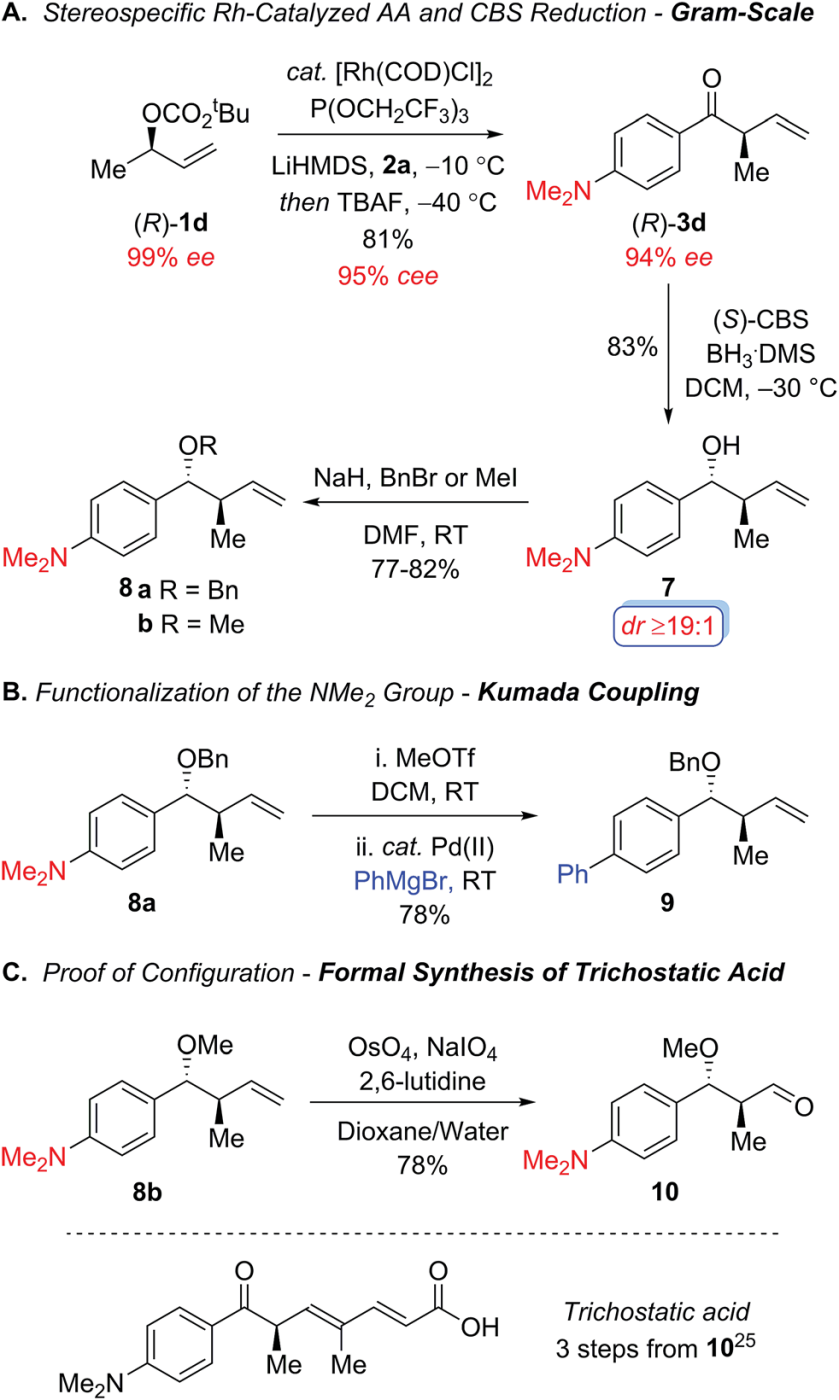
Stereospecific rhodium-catalyzed allylic alkylation to form α-ternary aryl ketone (*R*)-**3d** and further functionalization reactions.

Scheme 2 outlines additional studies to highlight the synthetic utility of this protocol using the chiral nonracemic secondary allylic carbonate (*R*)-**1d**, which addresses the stereospecificity of this process. In accord with our previous studies using enantiomerically enriched tertiary carbonates,^12^ the electron-poor tris(2,2,2-trifluoroethyl)-phosphite ligand proved optimal, furnishing the acyclic ketone (*R*)-**3d** with excellent regiocontrol (*b*/*l* ≥19 : 1) and conservation of enantiomeric excess (95% *cee*) on gram scale (Scheme 2A).^18^ Stereoselective reduction of the resulting aryl ketone (*R*)-**3d** with the Corey–Bakshi–Shibata reagent^19^ afforded the secondary alcohol **7** with excellent diastereocontrol, which was subsequently protected as the benzyl ether **8a** and methyl ether **8b** under standard reaction conditions to provide the intermediates required to functionalize the dimethylamino group and establish the absolute configuration in the allylic alkylation. Scheme 2B illustrates the metal-catalyzed cross-coupling of the dimethylamino group in order to broaden the scope of this process in the context of the aryl ketone component.^20,21^ For instance, Reeves and coworkers recently reported an elegant one-pot palladium-catalyzed Kumada coupling with aryl trimethylammonium salts at room temperature.^22^ To this end, the quaternization of the amine **8a** with methyl triflate followed by palladium-catalyzed Kumada coupling with phenylmagnesium bromide furnished the biaryl **9**, which illustrates the synthetic utility of the dialkylamino motif. Additional studies focused on establishing the stereochemical course of the allylic alkylation through a formal synthesis of trichostatic acid, which is a hydrolysis product of the potent histone deacetylase inhibitor (+)-trichostatin A (Scheme 2C).^23^ Oxidative cleavage of the olefin **8b** using Jin's modification of the Lemieux-Johnson oxidation,^24^ furnished the known aldehyde **10** ^25^ which confirmed the alkylation proceeds *via* net retention of configuration. Overall, this work represents an important advance for the metal-catalyzed allylic alkylation of secondary allylic alcohol derivatives with an acyl anion equivalent to prepare acyclic α-ternary β,γ-unsaturated aryl ketones in a highly regio- and stereospecific manner.

## Conclusions

In conclusion, we have developed an approach to acyclic α-ternary β,γ-unsaturated aryl ketones *via* the rhodium-catalyzed allylic alkylation of secondary allylic carbonates with an acyl anion equivalent. The ability to control the degree of isomerization using a dimethylamino-substituted aryl cyanohydrin to modulate the p*K*_a_ of the resulting α-proton in the aryl ketone is a critical component to the successful development of this method. Furthermore, the dialkylamine-substituted cyanohydrin facilitates the regio- and stereospecific construction of α-ternary β,γ-unsaturated ketones, which can either participate in the Kumada cross-coupling *via* the requisite trimethylammonium salts or be retained to facilitate a formal synthesis of trichostatic acid and thereby establish the stereochemical course of this process. Overall, this study constitutes a novel approach to the construction of enantiomerically enriched α-ternary β,γ-unsaturated aryl ketones, which represent important intermediates for target-directed synthesis, in addition to providing a strategy for controlling the isomerization of β,γ-unsaturated aryl ketones.
