# Supplementary material for: Regio- and stereospecific rhodium-catalyzed allylic alkylation with an acyl anion equivalent: an approach to acyclic α-ternary β,γ-unsaturated aryl ketones
Source: Chem Sci. 2017 Mar 31;8(5):4001–5. doi: 10.1039/c6sc05705e (PMC5433512; doi:10.1039/c6sc05705e)
Supplement: Supplementary file 1 [file SC-008-C6SC05705E-s001.pdf]

Electronic Supplementary Material (ESI) for Chemical Science.
This journal is © The Royal Society of Chemistry 2017

Regio- and Stereospecfic Rhodium-Catalyzed Allylic Alkylation with an Acyl Anion Equivalent: An Approach to Acyclic α-Ternary β,γ-Unsaturated Aryl Ketones

Ben W. H. Turnbull,[a] Jungha Chae,[b] Samuel Oliver[b] and P. Andrew Evans*,[a]

[a] *Department of Chemistry, Queen's University, 90 Bader Lane, Kingston, ON, K7L 3N6, Canada*

[b] *Department of Chemistry, The University of Liverpool, Crown Street, Liverpool, L69 7ZD, UK*

Contents of Supporting Information:

1. General Information S1
2. Representative Experimental Procedure for the Preparation of Secondary Allylic Carbonates **1a-i** S2
3. Spectral Data for the Secondary Allylic Carbonates **1a-i** S3
4. Spectral Data for the Aryl Cyanohydrins **2a-d** S6
5. Representative Experimental Procedure for the Rhodium-Catalyzed Allylic Alkylation with an Acyl Anion Equivalent S7
6. Spectral Data for the α-Ternary β,γ-Unsaturated Aryl Ketones **3a-l** S8
7. Stereospecific Rhodium-Catalyzed Allylic Alkylation/CBS reduction S13
8. Preparation of Biaryl **9** S16
9. Formal Synthesis of Trichostatic Acid S17
10. References S17
11. Proton and Carbon NMR Spectra S19
12. HPLC Chromatograms S80

1. General Information

All reactions were carried out under an argon atmosphere in anhydrous solvent using commercially available reagents that were purchased and used as received. Tetrahydrofuran was freshly distilled from sodium benzophenone ketyl. Analytical thin layer chromatography (TLC) was performed on pre-coated 0.25 mm thick silica gel 60-F_{254} plates (*Merck* or *Whatman PE SIL G/UV*); visualized using UV light and by treatment with a $KMnO_4$, followed by heating. All compounds were purified by flash chromatography using silica gel 60 (40-63 μm, *Silicycle* or *FluoroChem*) and gave spectroscopic data consistent with being ≥95% the assigned structure. Melting points (uncorrected) were obtained from a Büchi M560 melting point instrument.

Optical rotations ($[\alpha]_D^{°C}$) were measured on a *Anton Parr MCP 200* polarimeter with a tungsten halogen lamp (589 nm) at the stated temperature (indicated in ºC as superscript) using a 0.7 mL quartz cell of 100 mm length; solution concentrations (*c*) are given in g/100 mL. ^{1}H NMR and ^{13}C NMR spectra were recorded on a *Bruker Avance DRX-500* spectrometer in $CDCl_3$ at ambient temperature; chemical shifts (δ) are given in ppm and calibrated using the signal of residual undeuterated solvent as internal reference (δ_H = 7.26 ppm and δ_C = 77.16 ppm). ^{1}H NMR data are reported as follows: chemical shift (multiplicity, 1st order spin system if available, coupling constant, integration). Coupling constants (*J*) are reported in Hz and apparent splitting patterns are designated using the following abbreviations: s (singlet), d (doublet), t (triplet), q (quartet), m (multiplet), br (broad), app. (apparent) and the appropriate combinations. ^{13}C NMR spectra with complete proton decoupling were described with the aid of an APT sequence, separating methylene and quaternary carbons (e, even), from methyl and methine carbons (o, odd). IR spectra were recorded on an *Agilent Technologies Cary 630 FT-IR* (ATR); wavenumbers (ν) are given in cm^{-1}; and the abbreviations w (weak, <33%), m (medium, 33-66%), s (strong, >66%), vs (very strong, ≥95%) and br (broad) are used to describe the relative intensities of the IR absorbance bands. Mass spectra were obtained through the Queen's University Mass Spectrometry and Proteomics Services Unit. All liquid chromatographs were obtained on an Agilent 1260 Infinity series HPLC equipped with a variable wavelength UV detector. The instrument was fitted with a CHIRALPAK AS-H™ column (Diacel, 4.6 mm × 25cm).

2. Representative Experimental Procedure for the Preparation of Secondary Allylic Carbonates 1a-i.

Lithium bis(trimethylsilyl)amide (6.50 mL, 6.50 mmol; 1M solution in THF) was added dropwise to a stirred solution 5-phenylpent-1-en-3-ol (811 mg, 5.0 mmol) in anhydrous tetrahydrofuran (50 mL) at 0 °C under an atmosphere of argon. The mixture was stirred for *ca.* 15 minutes at this temperature and 2-(boc-oxyimino)-2-phenylacetonitrile (1.85 g, 7.5 mmol) was added. The reaction was then allowed to slowly warm to room temperature, stirred for *ca.* 16 hours and quenched by the dropwise addition of saturated aqueous ammonium chloride solution (10 mL). The reaction mixture was then partitioned between diethyl ether and saturated aqueous ammonium chloride solution. The combined organic layers were dried (anhyd. $MgSO_4$), filtered and concentrated *in vacuo* to afford the crude product. Purification by flash column chromatography (silica gel, eluting with 2-6% diethyl ether/hexane) afforded the *carbonate* **1a** (1.18 g, 90%) as a pale yellow oil.

3. Spectral Data for the Secondary Allylic Carbonates 1a-i

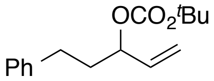

***tert*-Butyl 5-phenylpent-1-en-3-yl carbonate (1a)**

Color and State: Pale yellow oil

^{1}H NMR (500 MHz, $CDCl_3$) δ 7.30-7.27 (m, 2H), 7.21-7.18 (m, 3H), 5.84 (ddd, J = 17.2, 10.6, 6.7 Hz, 1H), 5.31 (d, J = 17.3 Hz, 1H), 5.23 (d, J = 10.5 Hz, 1H), 5.04 (app. q, J = 6.7 Hz, 1H), 2.72 (ddd, A of ABXY, J_{AB} = 14.0 Hz, J_{AX} = 10.1 Hz, J_{AY} = 6.0 Hz, 1H), 2.67 (ddd, B of ABXY, J_{AB} = 13.9 Hz, J_{BX} = 9.9 Hz, J_{BY} = 6.3 Hz, 1H), 2.05 (dddd, A of ABXYZ, J_{AB} = 13.8 Hz, J_{AX} = 9.8 Hz, J_{AY} = 7.6 Hz, J_{AZ} = 6.1 Hz, 1H), 1.91 (ddt, B of ABXYZ, J_{AB} = 13.9 Hz, J_{BX}= 10.0 Hz, J_{BY} = J_{BZ} = 6.1 Hz, 1H), 1.51 (s, 9H).

^{13}C NMR (125 MHz, $CDCl_3$) δ 153.08 (e), 141.38 (e), 136.30 (o), 128.56 (o), 128.50 (o), 126.11 (o), 117.43 (e), 82.14 (e), 77.44 (o), 36.03 (e), 31.51 (e), 27.94 (o).

IR (Neat) 3087 (w), 3065 (w), 3028 (w), 2981 (w), 2935 (w), 2866 (w), 1736 (s), 1647 (w), 1604 (w), 1498 (w), 1369 (m), 1272 (s), 1252 (vs), 1156 (s), 928 (m), 699 (s) cm^{-1}.

HRMS (ESI, $[M+Na]^+$) calcd for $C_{16}H_{22}O_3Na$ 285.1461, found 285.1467.

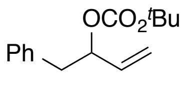

***tert*-Butyl 1-phenylbut-3-en-2-yl carbonate (1b)**

Color and State: Pale yellow oil

^{1}H NMR (500 MHz, $CDCl_3$) δ 7.30-7.27 (m, 2H), 7.23-7.20 (m, 3H), 5.83 (ddd, J = 17.2, 10.6, 6.5 Hz, 1H), 5.25 (app. q, J = 6.8 Hz, 1H), 5.23 (d, J = 17.1 Hz, 1H), 5.17 (d, J = 10.6 Hz, 1H), 3.04 (dd, A of ABX, J_{AB} = 13.7 Hz, J_{AX} = 7.2 Hz, 1H), 2.89 (dd, B of ABX, J_{AB} = 13.7 Hz, J_{BX} = 6.4 Hz, 1H), 1.44 (s, 9H).

^{13}C NMR (125 MHz, $CDCl_3$) δ 152.89 (e), 136.86 (e), 135.71 (o), 129.72 (o), 128.44 (o), 126.67 (o), 117.44 (e), 82.14 (e), 78.28 (o), 41.18 (e), 27.88 (o).

IR (Neat) 3088 (w), 3065 (w), 3031 (w), 2982 (w), 2935 (w), 1736 (s), 1648 (w), 1604 (w), 1498 (w), 1369 (m), 1272 (s), 1251 (vs), 1156 (s), 929 (m), 699 (s) cm^{-1}.

HRMS (ESI, $[M+Na]^+$) calcd for $C_{15}H_{20}O_3Na$ 271.1305, found 271.1296.

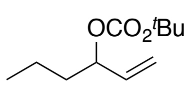

***tert*-Butyl hex-1-en-3-yl carbonate (1c)**

Color and State: Colorless oil

^{1}H NMR (500 MHz, $CDCl_3$) δ 5.77 (ddd, J = 17.3, 10.5, 6.7 Hz, 1H), 5.24 (d, J = 17.1 Hz, 1H), 5.15 (d, J = 10.4 Hz, 1H), 4.97 (app. q, J = 6.7 Hz, 1H), 1.69-1.62 (m, 1H), 1.58-1.50 (m, 1H), 1.46 (s, 9H), 1.41-1.29 (m, 2H), 0.90 (t, J = 7.3 Hz, 3H).

^{13}C NMR (125 MHz, $CDCl_3$) δ 153.18 (e), 136.60 (o), 116.96 (e), 81.90 (e), 77.92 (o), 36.47 (e), 27.91 (o), 18.47 (e), 13.88 (o).

IR (Neat) 3088 (w), 2963 (w), 2937 (w), 2876 (w), 1738 (s), 1648 (w), 1369 (m), 1273 (s), 1251 (vs), 1162 (s), 929 (m) cm^{-1}.

HRMS (ESI, $[M+Na]^+$) calcd for $C_{11}H_{20}O_3Na$ 223.1305, found 223.1310.

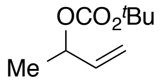

But-3-en-2-yl *tert*-butyl carbonate (1d)

Color and State: Colorless oil

^{1}H NMR (500 MHz, $CDCl_3$) δ 5.86 (ddd, J = 17.1, 10.7, 6.3 Hz, 1H), 5.27 (app. dt, J = 17.3, 1.2 Hz, 1H), 5.15 (app. dt, J = 10.5, 1.5 Hz, 1H), 5.13 (app. pentet, J = 6.4 Hz, 1H), 1.49 (s, 9H), 1.35 (d, J = 6.6 Hz, 3H).

^{13}C NMR (125 MHz, $CDCl_3$) δ 152.96 (e), 137.62 (o), 116.17 (e), 81.97 (e), 74.11 (o), 27.91 (o), 20.14 (o).

IR (Neat) 3091 (w), 2982 (w), 2937 (w), 1736 (s), 1647 (w), 1370 (m), 1273 (s), 1252 (vs), 1162 (s) cm^{-1}.

HRMS (ESI, $[M+H]^+$) calcd for $C_9H_{17}O_3$ 173.1172, found 173.1169.

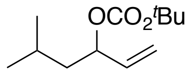

***tert*-Butyl 5-methylhex-1-en-3-yl carbonate (1e)**

Color and State: Colorless oil

^{1}H NMR (500 MHz, $CDCl_3$) δ 5.79 (ddd, J = 17.3, 10.5, 6.8 Hz, 1H), 5.28 (app. dt, J = 17.3, 1.2 Hz, 1H), 5.17 (app. dt, J = 10.5, 1.1 Hz, 1H), 5.07 (app. qq, J = 6.9, 0.9 Hz, 1H), 1.67 (nonet, J = 6.6 Hz, 1H), 1.63 (app. dt, A of ABXY, J_{AB} = 13.4 Hz, J_{AX} = J_{AY} = 6.8 Hz, 1H), 1.48 (s, 9H), 1.39 (app. dt, B of ABXY, J_{AB} = 13.2 Hz, J_{BX} = J_{BY} = 6.2 Hz, 1H), 0.93 (d, J = 6.3 Hz, 3H), 0.92 (d, J = 6.6 Hz, 3H).

^{13}C NMR (125 MHz, $CDCl_3$) δ 153.17 (e), 136.89 (o), 116.93 (e), 81.95 (e), 76.64 (o), 43.38 (e), 27.95 (o), 24.49 (o), 22.75 (o), 22.53 (o).

IR (Neat) 3088 (w), 2959 (w), 2935 (w), 2873 (w), 1738 (s), 1648 (w), 1369 (m), 1274 (s), 1252 (vs), 1161 (s), 931 (m) cm^{-1}.

HRMS (ESI, $[M+Na]^+$) calcd for $C_{12}H_{22}O_3Na$ 237.1461, found 237.1464.

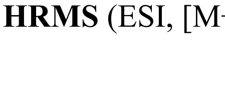

***tert*-Butyl 6-methylhept-1-en-3-yl carbonate (1f)**

Color and State: Pale yellow oil

^{1}H NMR (500 MHz, $CDCl_3$) δ 5.79 (ddd, J = 17.3, 10.5, 6.8 Hz, 1H), 5.26 (app. dt, J = 17.3, 1.2 Hz, 1H), 5.18 (app. dt, J = 10.5, 1.1 Hz, 1H), 4.96 (app. q, J = 6.7 Hz, 1H), 1.74-1.65 (m, 1H), 1.62-1.52 (m, 2H), 1.48 (s, 9H), 1.29-1.15 (m, 2H), 0.88 (d, J = 6.6 Hz, 3H), 0.88 (d, J = 6.6 Hz, 3H).

^{13}C NMR (125 MHz, $CDCl_3$) δ 153.18 (e), 136.63 (o), 117.08 (e), 81.94 (e), 78.44 (o), 34.17 (e), 32.30 (e), 27.95 (o), 22.64 (o), 22.58 (o).

IR (Neat) 3085 (w), 2957 (w), 2936 (w), 2872 (w), 1737 (s), 1648 (w), 1369 (m), 1273 (vs), 1252 (s), 1161 (s), 930 (m) cm^{-1}.

HRMS (ESI, $[M+Na]^+$) calcd for $C_{13}H_{24}O_3Na$ 251.1618, found 251.1612.

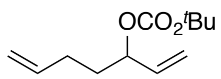

***tert*-Butyl hepta-1,6-dien-3-yl carbonate (1g)**

Color and State: Colorless oil.

^{1}H NMR (500 MHz, $CDCl_3$) δ 5.80 (ddt, J = 17.3, 10.4, 6.8 Hz, 1H), 5.79 (ddd, J = 17.3, 10.4, 6.8 Hz, 1H), 5.28 (app. dt, J = 17.3, 1.1 Hz, 1H), 5.20 (app. dt, J = 10.5, 1.1 Hz, 1H), 5.03 (app. dq, J = 17.2, 1.7 Hz, 1H), 5.01 (app. q, J = 6.4 Hz, 1H), 4.98 (app. dq, J = 10.1, 1.4 Hz, 1H), 2.14-2.10 (m, 2H), 1.81 (app. dq, A of $ABXY_2$, J_{AB} = 13.8 Hz, J_{AX} = J_{AY} = 7.4 Hz, 1H), 1.68 (ddt, B of $ABXY_2$, J_{AB} = 13.7 Hz, J_{BX} = 8.4 Hz, J_{BY} = 6.5 Hz, 1H), 1.48 (s, 9H).

^{13}C NMR (125 MHz, $CDCl_3$) δ 153.05 (e), 137.54 (o), 136.30 (o), 117.28 (e), 115.34 (e), 82.00 (e), 77.41 (o), 33.49 (e), 29.40 (e), 27.91 (o).

IR (Neat) 3080 (w), 2981 (w), 2935 (w), 2870 (w), 1738 (s), 1643 (w), 1369 (m), 1272 (s), 1253 (vs), 1160 (s) cm^{-1}.

HRMS (ESI, $[M+H]^+$) calcd for $C_{12}H_{21}O_3$ 213.1485, found 213.1475.

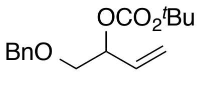

1-(Benzyloxy)but-3-en-2-yl *tert*-butyl carbonate (1h)

Color and State: Pale yellow oil.

^{1}H NMR (500 MHz, $CDCl_3$) δ 7.36-7.32 (m, 4H), 7.30-7.27 (m, 1H), 5.85 (ddd, J = 17.2, 10.7, 6.4 Hz, 1H), 5.37 (app. dt, J = 17.4, 1.3 Hz, 1H), 5.29-5.25 (m, 2H), 4.59 (d, A of AB, J_{AB} = 12.1 Hz, 1H), 4.56 (d, B of AB, J_{AB} = 12.2 Hz, 1H), 3.61 (dd, A of ABX, J_{AB} = 10.6 Hz, J_{AX} = 7.0 Hz, 1H), 3.57 (dd, B of ABX, J_{AB} = 10.7 Hz, J_{BX} = 4.4 Hz, 1H), 1.49 (s, 9H)

^{13}C NMR (125 MHz, $CDCl_3$) δ 152.99 (e), 138.06 (e), 133.34 (o), 128.47 (o), 127.76 (o), 127.74 (o), 118.36 (e), 82.30 (e), 76.23 (o), 73.31 (e), 71.48 (e), 27.91 (o).

IR (Neat) 3090 (w), 3067 (w), 3031 (w), 2982 (w), 2936 (w), 2865 (w), 1740 (s), 1648 (w), 1369 (m), 1273 (s), 1252 (s), 1160 (s), 910 (m), 697 (s) cm^{-1}.

HRMS (ESI, $[M+H]^+$) calcd for $C_{16}H_{23}O_4$ 279.1591, found 279.1579.

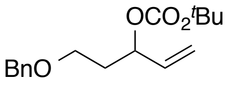

5-(Benzyloxy)pent-1-en-3-yl *tert*-butyl carbonate (1i)

Color and State: Pale yellow oil.

^{1}H NMR (500 MHz, $CDCl_3$) δ 7.36-7.32 (m, 4H), 7.30-7.27 (m, 1H), 5.81 (ddd, J = 17.2, 10.5, 6.7 Hz, 1H), 5.29 (app. dt, J = 17.2, 1.1 Hz, 1H), 5.21 (app. q, J = 6.7 Hz, 1H), 5.19 (app. dt,

10.5, 1.0 Hz, 1H), 4.51 (d, A of AB, J_{AB} = 12.0 Hz, 1H), 4.48 (d, B of AB, J_{AB} = 11.9 Hz, 1H), 3.55 (ddd, A of ABXY, J_{AB} = 12.9 Hz, J_{AX} = 6.9 Hz, J_{AY} = 6.1 Hz, 1H), 3.52 (dt, B of ABXY, J_{AB} = 12.5 Hz, J_{BX} = J_{BY} = 6.3 Hz, 1H), 2.02 (ddt, A of $ABXY_2$, J_{AB} = 14.0 Hz, J_{AX} = 7.8 Hz, J_{AY} = 6.2 Hz, 1H), 1.91 (app. dq, B of $ABXY_2$, J_{AB} = 12.9 Hz, J_{BX} = J_{BY} = 6.4 Hz, 1H), 1.48 (s, 9H).

^{13}C NMR (125 MHz, $CDCl_3$) δ 152.98 (e), 138.48 (e), 136.27 (o), 128.49 (o), 127.78 (o), 127.68 (o), 117.22 (e), 82.07 (e), 75.32 (o), 73.22 (e), 66.26 (e), 34.63 (e), 27.94 (o).

IR (Neat) 3067 (w), 3030 (w), 2981 (w), 2936 (w), 2865 (w), 1737 (s), 1648 (w), 1369 (m), 1273 (s), 1252 (vs), 1155 (s), 930 (m), 698 (s) cm^{-1}.

HRMS (ESI, $[M+Na]^+$) calcd for $C_{17}H_{24}O_4Na$ 315.1567, found 315.1554.

4. Spectral Data for the Aryl Cyanohydrins 2a-d

The aryl cyanohydrins **2a-d** were prepared from the corresponding aldehydes according to the previously reported procedure.[1,2] The spectral data for the cyanohydrins **2a′** and **2aa′** has been previously reported.[2b]

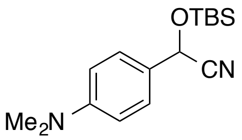

2-(*tert*-Butyldimethylsilyloxy)-2-(4-(dimethylamino)phenyl)acetonitrile (2a)

Color and State: Yellow oil.

^{1}H NMR (500 MHz, $CDCl_3$) δ 7.32-7.29 (m, 2H), 6.72-6.69 (m, 2H), 5.41 (s, 1H), 2.98 (s, 6H), 0.92 (s, 9H), 0.19 (s, 3H), 0.11 (s, 3H).

^{13}C NMR (125 MHz, $CDCl_3$) δ 151.16 (e), 127.63 (o), 124.06 (e), 119.90 (e), 112.29 (o), 64.16 (o), 40.48 (o), 25.71 (o), 18.30 (e), –4.90 (o).

IR (Neat) 2932 (w), 2889 (w), 2859 (w), 2813 (w), 1613 (m), 1524 (m), 1255 (m), 1063 (s), 835 (s), 777 (s) cm^{-1}.

HRMS (EI, M^+) calcd for $C_{16}H_{26}N_2OSi$ 290.1814, found 290.1819.

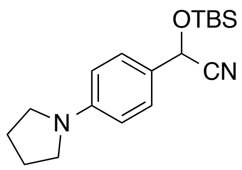

2-(*tert*-Butyldimethylsilyloxy)-2-(4-pyrrolidin-1-yl)phenyl)acetonitrile (2b)

Color and State: Yellow oil.

^{1}H NMR (500 MHz, $CDCl_3$) δ 7.30-7.27 (m, 2H), 6.56-6.53 (m, 2H), 3.40 (s, 1H), 3.31-3.28 (m, 4H), 2.03-2.00 (m, 4H), 0.92 (s, 9H), 0.18 (s, 3H), 0.10 (s, 3H).

^{13}C NMR (125 MHz, $CDCl_3$) δ 148.60 (e), 127.80 (o), 123.11 (e), 119.99 (e), 111.67 (o), 64.34 (o), 47.70 (e), 25.73 (o), 25.60 (e), 18.30 (e), –4.87 (o), –4.89 (o).

IR (Neat) 2955 (w), 2932 (w), 2857 (w), 1612 (m), 1524 (s), 1254 (m), 1180 (m), 1066 (m), 835 (s), 778 (s) cm^{-1}.

HRMS (EI, M$^+$) calcd for $C_{18}H_{28}N_2OSi$ 316.1971, found 316.1978.

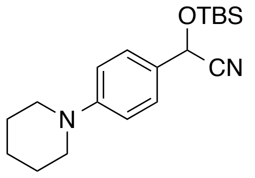

2-(*tert*-Butyldimethylsilyloxy)-2-(4-(piperidin-1-yl)phenyl)acetonitrile (2c)

Color and State: Yellow oil.

^{1}H NMR (500 MHz, $CDCl_3$) δ 7.33-7.30 (m, 2H), 6.93-6.90 (m, 2H), 5.43 (s, 1H), 3.20 (t, J = 5.5 Hz, 4H), 1.70 (pentet, J = 5.6 Hz, 4H), 1.62-1.57 (m, 2H), 0.93 (s, 9H), 0.20 (s, 3H), 0.12 (s, 3H).

^{13}C NMR (125 MHz, $CDCl_3$) δ 152.73 (e), 127.46 (o), 126.38 (e), 119.75 (e), 116.00 (o), 64.05 (o), 50.10 (e), 25.77 (e), 25.71 (o), 24.41 (e), 18.30 (e), –4.90 (o), –4.93 (o).

IR (Neat) 2932 (m), 2858 (w), 2809 (w), 1611 (m), 1516 (m), 1254 (m), 1238 (m), 1073 (m), 836 (s), 778 (s) cm^{-1}.

HRMS (EI, M$^+$) calcd for $C_{19}H_{30}N_2OSi$ 330.2127, found 330.2120.

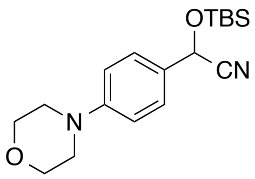

2-(*tert*-Butyldimethylsilyloxy)-2-(4-morpholinophenyl)acetonitrile (2d)

Color and State: Yellow oil.

^{1}H NMR (500 MHz, $CDCl_3$) δ 7.35 (d, J = 8.7 Hz, 2H), 6.91 (d, J = 8.7 Hz, 2H), 5.44 (s, 1H), 3.86 (t, J = 4.8 Hz, 4H), 3.19 (t, J = 4.9 Hz, 4H), 0.92 (s, 9H), 0.20 (s, 3H), 0.12 (s, 3H).

^{13}C NMR (125 MHz, $CDCl_3$) δ 151.98 (e), 127.61 (e), 127.53 (o), 119.64 (e), 115.50 (o), 66.91 (e), 63.91 (o), 48.92 (e), 25.69 (o), 18.30 (e), –4.91 (o), –4.96 (o).

IR (Neat) 2956 (w), 2930 (w), 2889 (w), 2857 (m), 1611 (m), 1518 (m), 1255 (m), 1235 (s), 1070 (s), 835 (vs), 779 (s) cm^{-1}.

HRMS (EI, M$^+$) calcd for $C_{18}H_{28}N_2O_2Si$ 332.1920, found 332.1928.

5. Representative Experimental Procedure for the Rhodium-Catalyzed Allylic Alkylation with an Acyl Anion Equivalent

Lithium bis(trimethylsilyl)amide (0.90 mL, 0.90 mmol; 1M solution in THF) was added dropwise to a stirred solution of 2-(*tert*-butyldimethylsilyloxy)-2-(4-(dimethylamino)phenyl)acetonitrile **2a** (189 mg, 0.65 mmol) in anhydrous tetrahydrofuran (2.5 mL) and DMPU (0.5 mL) at –10 °C under an atmosphere of argon. The anion was allowed to form over *ca.* 30 minutes, resulting in a green homogeneous solution. In a separate flask, $[RhCl(COD)]_2$ (6.20 mg, 0.013 mmol) and tris(*tert*-butyldimethylsilyl) phosphite (23.0 μL, 0.050 mmol) were dissolved in anhydrous tetrahydrofuran (2 mL) at room temperature under an atmosphere of argon. The mixture was

stirred for *ca.* 5 minutes, resulting in a light yellow homogeneous solution, and then cooled to –10 °C. The catalyst solution was then added to the anion *via* Teflon® cannula, followed immediately by the addition of *tert*-butyl 5-phenylpent-1-en-3-yl carbonate **1a** (131 mg, 0.50 mmol) *via* tared 500 µL gastight syringe. The mixture was allowed to stir for *ca.* 16 hours and then cooled to –40 °C (dry ice/acetonitrile bath). TBAF (2.00 mL, 2.00 mmol; 1M solution in THF) was then added dropwise and the solution was allowed to stir for *ca.* 1 hour at –40 °C. The reaction mixture was quenched with saturated aqueous ammonium chloride solution (2 mL), allowed to warm to room temperature and then partitioned between diethyl ether and saturated aqueous ammonium chloride solution. The combined organic layers were dried (anhyd. $MgSO_4$), filtered and concentrated *in vacuo* to give the crude product. Purification by flash column chromatography (silica gel, eluting with 3-9% diethyl ether/hexane) afforded the *ketone* **3a** (131 mg, 89%) as a white solid.

6. Spectral Data for the α-Ternary β,γ-Unsaturated Aryl Ketones 3a-l

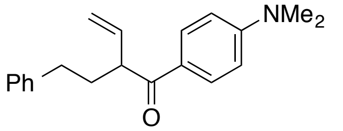

1-(4-(Dimethylamino)phenyl)-2-phenethylbut-3-en-1-one (3a)

Color and State: White solid; **mp** = 43-44 °C; *b:l* ≥19:1

^{1}H NMR (500 MHz, $CDCl_3$) δ 7.85-7.82 (m, 2H), 7.28-7.25 (m, 2H), 7.20-7.16 (m, 3H), 6.65-6.62 (m, 2H), 5.95 (ddd, J = 17.1, 10.3, 8.6 Hz, 1H), 5.16 (app. dt, J = 17.3, 1.1 Hz, 1H), 5.15 (app. dt, J = 10.3, 1.4 Hz, 1H), 3.99 (app. q, J = 7.5 Hz, 1H), 3.05 (s, 6H), 2.69-2.60 (m, 2H), 2.23 (ddt, A of $ABXY_2$, J_{AB} = 13.6 Hz, J_{AX} = 8.6 Hz, J_{AY} = 6.8 Hz, 1H), 1.91 (ddt, B of $ABXY_2$, J_{AB} = 13.8 Hz, J_{BX} = 8.3 Hz, J_{BY} = 7.0 Hz, 1H).

^{13}C NMR (125 MHz, $CDCl_3$) δ 198.67 (e), 153.52 (e), 142.06 (e), 138.03 (o), 130.80 (o), 128.70 (o), 128.42 (o), 125.92 (o), 124.65 (e), 117.14 (e), 110.80 (o), 50.08 (o), 40.09 (o), 33.84 (e), 33.45 (e).

IR (Neat) 3078 (w), 3058 (w), 3024 (w), 2915 (w), 2854 (w), 2816 (w), 1655 (m), 1590 (vs), 1548 (s), 1525 (m), 1367 (s), 1163 (s), 991 (m), 937 (m), 824 (m), 698 (s) cm^{-1}.

HRMS (EI, M^+) calcd for $C_{20}H_{23}NO$ 293.1780, found 293.1791.

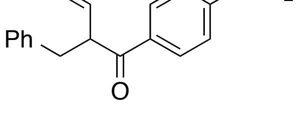

2-Benzyl-1-(4-(dimethylamino)phenyl)but-3-en-1-one (3b)

Color and State: White solid; **mp** = 40-42 °C; *b:l* ≥19:1

^{1}H NMR (500 MHz, $CDCl_3$) δ 7.90-7.87 (m, 2H), 7.27-7.21 (m, 4H), 7.18-7.14 (m, 1H), 6.64-6.62 (m, 2H), 5.96 (ddd, J = 17.2, 10.1, 8.5 Hz, 1H), 5.10 (d, J = 10.5 Hz, 1H), 5.07 (d, J = 17.3 Hz, 1H), 4.31 (app. q, J = 7.5 Hz, 1H), 3.28 (dd, A of ABX, J_{AB} = 13.8 Hz, J_{AX} = 6.9 Hz, 1H), 3.04 (s, 6H), 2.91 (dd, B of ABX, J_{AB} = 13.8 Hz, J_{BX} =7.4 Hz, 1H).

^{13}C NMR (125 MHz, $CDCl_3$) δ 198.13 (e), 153.49 (e), 140.05 (e), 137.54 (o), 130.84 (o), 129.34 (o), 128.29 (o), 126.11 (o), 124.55 (e), 117.45 (e), 110.76 (o), 52.80 (o), 40.09 (o), 38.32 (e).

IR (Neat) 3078 (w), 3032 (w), 2999 (w), 2912 (m), 2850 (w), 2744 (w), 1653 (m), 1591 (vs), 1549 (s), 1530 (m), 1374 (s), 1167 (s), 999 (s), 933 (s), 819 (s), 698 (s) cm^{-1}.

HRMS (EI, M^+) calcd for $C_{19}H_{21}NO$ 279.1623, found 279.1615.

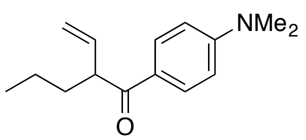

1-(4-(Dimethylamino)phenyl)-2-vinylpentan-1-one (3c)

Color and State: White solid; **mp** = 39-41 °C; *b:l* ≥19:1

^{1}H NMR (500 MHz, $CDCl_3$) δ 7.93-7.90 (m, 2H), 6.67-6.64 (m, 2H), 5.92 (ddd, J = 17.3, 10.0, 8.6 Hz, 1H), 5.13 (app. dt, J = 17.3, 0.6 Hz, 1H), 5.09 (dd, J = 10.2, 1.3 Hz, 1H), 4.00 (app. q, J = 7.6 Hz, 1H), 3.04 (s, 6H), 1.85 (ddt, A of ABXYZ, J_{AB} = 13.2 Hz, J_{AX} = 9.8 Hz, J_{AY} = J_{AZ} = 6.5 Hz, 1H), 1.58 (dddd, B of ABXYZ, J_{AB} = 13.1 Hz, J_{BX} = 10.0 Hz, J_{BY} = 7.6 Hz, J_{BZ} = 5.5 Hz, 1H), 1.42-1.25 (m, 2H), 0.91 (t, J = 7.3 Hz, 3H).

^{13}C NMR (125 MHz, $CDCl_3$) δ 199.23 (e), 153.46 (e), 138.42 (o), 130.73 (o), 124.76 (e), 116.48 (e), 110.79 (o), 50.78 (o), 40.09 (o), 34.65 (e), 20.61 (e), 14.15 (o).

IR (Neat) 3079 (w), 2956 (w), 2930 (w), 2870 (w), 2819 (w), 1655 (m), 1591 (vs), 1551 (m), 1527 (m), 1367 (s), 1185 (s), 1167 (s), 994 (m), 823 (s) cm^{-1}.

HRMS (EI, M^+) calcd for $C_{15}H_{21}NO$ 231.1623, found 231.1630.

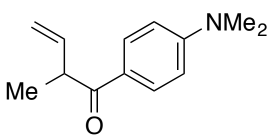

1-(4-(Dimethylamino)phenyl)-2-methylbut-3-en-1-one (3d)

Color and State: Yellow solid; **mp** = 45-47 °C; *b:l* ≥19:1

^{1}H NMR (500 MHz, $CDCl_3$) δ 7.93-7.90 (m, 2H), 6.67-6.64 (m, 2H), 6.01 (ddd, J = 17.4, 10.1, 7.5 Hz, 1H), 5.15 (app. dt, J = 17.2, 1.3 Hz, 1H), 5.09 (app. dt, J = 10.2, 1.0 Hz, 1H), 4.11 (app. pentet, J = 7.1 Hz, 1H), 3.05 (s, 6H), 1.31 (d, J = 6.8 Hz, 3H).

^{13}C NMR (125 MHz, $CDCl_3$) δ 199.32 (e), 153.45 (e), 139.34 (o), 130.84 (o), 124.19 (e), 115.66 (e), 110.78 (o), 44.77 (o), 40.10 (o), 17.41 (o).

IR (Neat) 3079 (w), 2974 (w), 2929 (w), 2870 (w), 2816 (w), 1659 (m), 1634 (w), 1591 (vs), 1550 (s), 1526 (s), 1366 (s), 1187 (s), 1160 (s), 944 (s), 825 (s) cm^{-1}.

HRMS (EI, M^+) calcd for $C_{13}H_{17}NO$ 203.1310, found 203.1312.

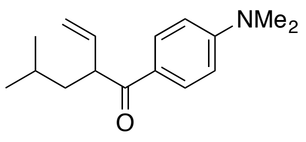

1-(4-(Dimethylamino)phenyl)-4-methyl-2-vinylpentan-1-one (3e)

Color and State: Pale yellow solid; **mp** = 61-63 °C; *b:l* ≥19:1

^{1}H NMR (500 MHz, $CDCl_3$) δ 7.93-7.90 (m, 2H), 6.68-6.65 (m, 2H), 5.90 (ddd, J = 17.2, 10.1, 8.7 Hz, 1H), 5.13 (app. dt, J = 17.2, 1.1 Hz, 1H), 5.09 (dd, J = 10.2, 1.4

Hz, 1H), 4.10 (app. q, J = 7.7 Hz, 1H), 3.06 (s, 6H), 1.74 (ddd, A of ABXY, J_{AB} = 13.3 Hz, J_{AX} = 7.6 Hz, J_{AY} = 6.7 Hz, 1H), 1.65-1.57 (m, 1H), 1.50 (ddd, B of ABXY, J_{AB} = 13.4 Hz, J_{BX} = 7.6 Hz, J_{BY} = 6.0 Hz, 1H), 0.92 (d, J = 6.5 Hz, 3H), 0.90 (d, J = 6.6 Hz, 3H).

^{13}C NMR (125 MHz, $CDCl_3$) δ 199.21 (e), 153.48 (e), 138.55 (o), 130.74 (o), 124.69 (e), 116.45 (e), 110.84 (o), 48.96 (o), 41.46 (e), 40.13 (o), 25.75 (o), 23.15 (o), 22.40 (o).

IR (Neat) 3079 (w), 2954 (m), 2930 (m), 2870 (m), 2808 (w), 1649 (m), 1596 (vs), 1548 (s), 1529 (m), 1368 (s), 1187 (s), 1169 (s), 999 (m), 826 (s) cm^{-1}.

HRMS (EI, M^+) calcd for $C_{16}H_{23}NO$ 245.1780, found 245.1768.

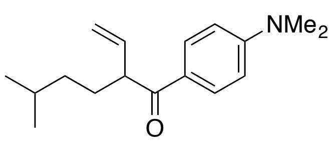

1-(4-(Dimethylamino)phenyl)-5-methyl-2-vinylhexan-1-one (3f)

Color and State: Yellow oil; *b:l* ≥19:1

^{1}H NMR (500 MHz, $CDCl_3$) δ 7.93-7.90 (m, 2H), 6.68-6.65 (m, 2H), 5.91 (ddd, J = 17.2, 10.1, 8.6 Hz, 1H), 5.13 (app. dt, J = 17.3, 1.1 Hz, 1H), 5.10 (dd, J = 10.2, 1.4 Hz, 1H), 3.94 (app. q, J = 7.6 Hz, 1H), 3.06 (s, 6H), 1.91-1.83 (m, 1H), 1.59 (dddd, A of ABXYZ, J_{AB} = 13.1 Hz, J_{AX} = 11.3 Hz, J_{AY} = 7.6 Hz, J_{AZ} = 5.3 Hz, 1H), 1.55 (nonet, J = 6.7 Hz, 1H), 1.25-1.12 (m, 2H), 0.87 (d, J = 6.6 Hz, 3H), 0.86 (d, J = 6.6 Hz, 3H).

^{13}C NMR (125 MHz, $CDCl_3$) δ 199.28 (e), 153.48 (e), 138.50 (o), 130.75 (o), 124.80 (e), 116.55 (e), 110.80 (o), 51.32 (o), 40.11 (o), 36.68 (e), 30.39 (e), 28.21 (o), 22.75 (o), 22.58 (o).

IR (Neat) 3079 (w), 2952 (m), 2904 (w), 2868 (w), 1658 (m), 1633 (w), 1592 (vs), 1551 (m), 1527 (m), 1366 (s), 1185 (s), 1167 (s), 994 (m), 824 (m) cm^{-1}.

HRMS (EI, M^+) calcd for $C_{17}H_{25}NO$ 259.1936, found 259.1939.

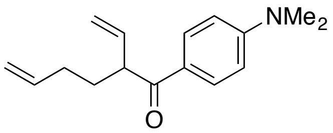

1-(4-(Dimethylamino)phenyl)2-vinylhex-5-en-1-one (3g)

Color and State: Pale yellow oil; *b:l* ≥19:1

^{1}H NMR (500 MHz, $CDCl_3$) δ 7.92-7.89 (m, 2H), 6.67-6.64 (m, 2H), 5.90 (ddd, J = 17.2, 10.2, 8.7 Hz, 1H), 5.80 (ddt, J = 17.0, 10.3, 6.7 Hz, 1H), 5.15 (app. dt, J = 17.0, 1.0 Hz, 1H), 5.12 (dd, J = 10.2, 1.4 Hz, 1H), 5.00 (app. dq, J = 17.2, 1.7 Hz, 1H), 4.97 (ddt, J = 10.1, 2.0, 1.1 Hz, 1H), 4.03 (app. q, J = 7.5 Hz, 1H), 3.06 (s, 6H), 2.11-2.06 (m, 2H), 1.98 (ddt, A of $ABXY_2$, J_{AB} = 13.3 Hz, J_{AX} = 8.7 Hz, J_{AY} = 6.6 Hz, 1H), 1.69 (app. dq, B of $ABXY_2$, J_{AB} = 13.5 Hz, J_{BX} = J_{BY} = 7.2 Hz, 1H).

^{13}C NMR (125 MHz, $CDCl_3$) δ 198.89 (e), 153.50 (e), 138.36 (o), 138.07 (o), 130.79 (o), 124.68 (e), 116.97 (e), 115.19 (e), 110.79 (o), 50.10 (o), 40.11 (o), 31.43 (e), 31.37 (e).

IR (Neat) 3076 (w), 2976 (w), 2920 (w), 2855 (w), 2818 (w), 1655 (m), 1591 (vs), 1551 (m), 1527 (m), 1368 (s), 1185 (s), 1166 (s), 993 (m), 821 (s) cm^{-1}.

HRMS (EI, M+) calcd for $C_{16}H_{21}NO$ 243.1623, found 243.1631.

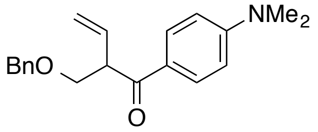

2-(Benzyloxymethyl)-1-(4-(dimethylamino)phenyl)but-3-en-1-one (3h)

Color and State: Yellow oil; *b:l* ≥19:1

^{1}H NMR (500 MHz, $CDCl_3$) δ 7.93-7.90 (m, 2H), 7.32-7.23 (m, 5H), 6.67-6.64 (m, 2H), 5.92 (ddd, *J* = 17.4, 10.1, 8.0 Hz, 1H), 5.22 (d, *J* = 17.3 Hz, 1H), 5.17 (d, *J* = 10.2 Hz, 1H), 4.56 (d, A of AB, J_{AB} = 12.1 Hz, 1H), 4.51 (d, B of AB, J_{AB} = 12.1 Hz, 1H), 4.39 (app. q, *J* = 7.3 Hz, 1H), 3.97 (dd, A of ABX, J_{AB} = 9.1 Hz, J_{AX} = 7.4 Hz, 1H), 3.66 (dd, B of ABX, J_{AB} = 9.1 Hz, J_{BX} = 6.4 Hz, 1H), 3.06 (s, 6H).

^{13}C NMR (125 MHz, $CDCl_3$) δ 197.02 (e), 153.55 (e), 138.43 (e), 135.04 (o), 130.92 (o), 128.36 (o), 127.68 (o), 127.54 (o), 124.73 (e), 118.35 (e), 110.75 (o), 73.41 (e), 71.44 (e), 51.20 (o), 40.07 (o).

IR (Neat) 3082 (w), 3061 (w), 3029 (w), 2900 (w), 2857 (w), 1654 (m), 1590 (vs), 1550 (m), 1527 (m), 1368 (s), 1186 (s), 1168 (s), 1093 (s), 994 (m), 825 (s), 697 (s) cm^{-1}.

HRMS (EI, M+) calcd for $C_{20}H_{23}NO_2$ 309.1729, found 309.1721.

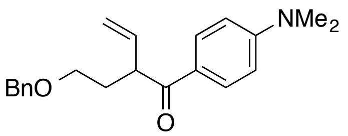

2-(2-(Benzyloxy)ethyl)-1-(4-(dimethylamino)phenyl)but-3-en-1-one (3i)

Color and State: Pale yellow oil; *b:l* ≥19:1

^{1}H NMR (500 MHz, $CDCl_3$) δ 7.95-7.92 (m, 2H), 7.33-7.24 (m, 5H), 6.66-6.63 (m, 2H), 5.90 (ddd, *J* = 17.2, 10.1, 8.6 Hz, 1H), 5.14 (ddd, *J* = 17.3, 1.4, 0.9 Hz, 1H), 5.11 (ddd, *J* = 10.1, 1.4, 0.5 Hz, 1H), 4.46 (d, A of AB, J_{AB} = 12.0 Hz, 1H), 4.44 (d, B of AB, J_{AB} = 12.0 Hz, 1H), 4.30 (app. q, *J* = 7.6 Hz, 1H), 3.54 (ddd, A of ABXY, J_{AB} = 9.5 Hz, J_{AX} = 7.2 Hz, J_{AY} = 5.2 Hz, 1H), 3.47 (ddd, B of ABXY, J_{AB} = 9.5 Hz, J_{BX} = 6.4 Hz, J_{BY} = 5.3 Hz, 1H), 3.06 (s, 6H), 2.23 (dtd, A of ABXYZ, J_{AB} = 14.1 Hz, J_{AX} = J_{AY} = 7.1 Hz, J_{AZ} = 5.3 Hz, 1H), 1.87 (dddd, B of ABXYZ, J_{AB} = 14.0 Hz, J_{BX} = 7.2 Hz, J_{BY} = 6.6 Hz, J_{BZ} = 5.3 Hz, 1H).

^{13}C NMR (125 MHz, $CDCl_3$) δ 198.76 (e), 153.50 (e), 138.66 (e), 137.62 (o), 130.92 (o), 128.39 (o), 127.73 (o), 127.55 (o), 124.63 (e), 117.19 (e), 110.77 (o), 72.99 (e), 67.88 (e), 47.29 (o), 40.10 (o), 32.26 (e).

IR (Neat) 3078 (w), 3030 (w), 2919 (w), 2856 (w), 1655 (m), 1591 (vs), 1551 (m), 1527 (m), 1366 (s), 1185 (s), 1167 (s), 1099 (s), 994 (m), 823 (s), 697 (s) cm^{-1}.

HRMS (EI, M+) calcd for $C_{21}H_{25}NO_2$ 323.1885, found 323.1880.

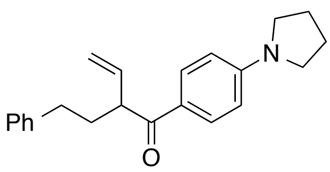

2-Phenethyl-1-(4-(pyrrolidine-1-yl)phenyl)but-3-en-1-one (3j)

Color and State: White solid; **mp** = 80-82 °C; *b:l* ≥19:1

^{1}H NMR (500 MHz, $CDCl_3$) δ 7.85-7.82 (m, 2H), 7.29-7.26 (m, 2H), 7.20-7.17 (m, 3H), 6.51-6.48 (m, 2H), 5.95 (ddd, *J* = 17.2, 10.2, 8.6 Hz, 1H), 5.16 (dd, *J* = 17.3, 0.9 Hz, 1H), 5.14 (dd, *J* = 10.2, 1.2, 1H), 3.99 (app. q, *J* = 7.1 Hz, 1H), 3.38-3.35 (m, 4H), 2.66-2.63 (m, 2H), 2.23 (ddt, A of $ABXY_2$, J_{AB} = 13.6 Hz, J_{AX} = 8.6 Hz, J_{AY} = 6.9 Hz, 1H), 2.05-2.02 (m, 4H), 1.91 (ddt, B of $ABXY_2$, J_{AB} = 13.8 Hz, J_{BX} = 8.2 Hz, J_{BY} = 7.0 Hz, 1H).

^{13}C NMR (125 MHz, $CDCl_3$) δ 198.62 (e), 151.11 (e), 142.11 (e), 138.14 (o), 130.98 (o), 128.72 (o), 128.42 (o), 125.91 (o), 124.14 (e), 117.06 (e), 110.85 (o), 50.00 (o), 47.66 (e), 33.88 (e), 33.47 (e), 25.56 (e).

IR (Neat) 3058 (w), 3023 (w), 2964 (w), 2944 (w), 2925 (w), 2848 (w), 1654 (m), 1588 (vs), 1546 (m), 1524 (m), 1388 (s), 1244 (m), 1177 (s), 1118 (m), 699 (m) cm^{-1}.

HRMS (EI, M^+) calcd for $C_{22}H_{25}NO$ 319.1936, found 319.1931.

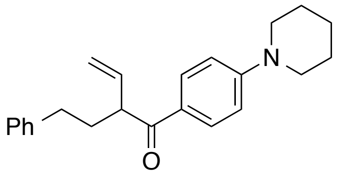

2-Phenethyl-1-(4-piperidin-1-yl)but-3-en-1-one (3k)

Color and State: White solid; **mp** = 81-82°C; *b:l* ≥19:1

^{1}H NMR (500 MHz, $CDCl_3$) δ 7.83-7.80 (m, 2H), 7.29-7.26 (m, 2H), 7.20-7.16 (m, 3H), 6.84-6.81 (m, 2H), 5.98-5.91 (m, 1H), 5.16 (d, *J* = 16.7 Hz, 1H), 5.16 (dd, *J* = 10.2, 1.1 Hz, 1H), 3.99 (app. q, *J* = 7.5 Hz, 1H), 3.37-3.35 (m, 4H), 2.66 (dt, A of ABX_2, J_{AB} = 13.9 Hz, J_{AX} = 6.6 Hz, 1H), 2.63 (dt, B of ABX_2, J_{AB} = 13.7 Hz, J_{BX} = 7.1 Hz, 1H), 2.22 (dtd, A of ABX_2Y, J_{AB} = 13.6 Hz, J_{AX} = 6.8 Hz, J_{AY} = 1.8 Hz, 1H), 1.91 (dtd, B of ABX_2Y, J_{AB} = 13.9 Hz, J_{BX} = 6.8 Hz, J_{BY} = 1.5 Hz, 1H), 1.68-1.64 (m, 6H).

^{13}C NMR (125 MHz, $CDCl_3$) δ 198.71 (e), 154.47 (e), 142.00 (e), 137.86 (o), 130.77 (o), 128.69 (o), 128.43 (o), 125.94 (o), 125.83 (e), 117.27 (e), 113.29 (o), 50.12 (o), 48.60 (e), 33.80 (e), 33.42 (e), 25.46 (e), 24.47 (e).

IR (Neat) 3076 (w), 3024 (w), 2931 (m), 2853 (w), 1659 (m), 1592 (vs), 1557 (m), 1516 (m), 1451 (m), 1387 (m), 1223 (s), 1188 (s), 1124 (s), 699 (m) cm^{-1}.

HRMS (EI, M^+) calcd for $C_{23}H_{27}NO$ 333.2093, found 333.2099.

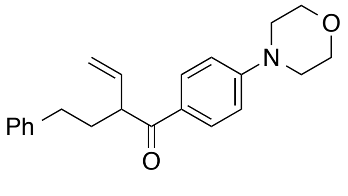

1-(4-Morpholinophenyl)-2-phenethylbut-3-en-1-one (3l)

Color and State: White solid; **mp** = 86-88 °C; *b:l* ≥19:1

^{1}H NMR (500 MHz, $CDCl_3$) δ 7.85-7.82 (m, 2H), 7.29-7.26 (m, 2H), 7.20-7.16 (m, 3H), 6.85-6.82 (m, 2H), 5.94 (ddd, *J* = 17.4,

10.0, 8.5 Hz, 1H), 5.18-5.15 (m, 2H), 3.98 (app. q, $J = 7.5$ Hz, 1H), 3.85 (br. t, $J = 4.9$ Hz, 4H), 3.30 (br. t, $J = 4.9$ Hz, 4H), 2.69-2.60 (m, 2H), 2.22 (ddt, A of $ABXY_2$, $J_{AB} = 13.5$ Hz, $J_{AX} = 8.6$ Hz, $J_{AY} = 6.8$ Hz, 1H), 1.92 (ddt, B of $ABXY_2$, $J_{AB} = 13.7$ Hz, $J_{BX} = 8.4$ Hz, $J_{BY} = 6.9$ Hz, 1H).

^{13}C NMR (125 MHz, $CDCl_3$) δ 198.92 (e), 154.31 (e), 141.91 (e), 137.63 (o), 130.66 (o), 128.70 (o), 128.47 (o), 127.46 (e), 126.00 (o), 117.50 (e), 113.40 (o), 66.68 (e), 50.26 (o), 47.62 (e), 33.74 (e), 33.38 (e).

IR (Neat) 3078 (w), 3060 (w), 3023 (w), 2958 (w), 2919 (w), 2854 (w), 1662 (m), 1596 (vs), 1516 (w), 1449 (w), 1381 (w), 1223 (m), 1190 (m), 1122 (m), 701 (w) cm^{-1}.

HRMS (EI, M^+) calcd for $C_{22}H_{25}NO_2$ 335.1885, found 335.1891.

7. Stereospecific Rhodium-Catalyzed Allylic Alkylation/CBS reduction

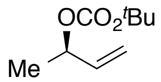

(*R*)-But-3-en-2-yl *tert*-butyl carbonate ((*R*)-1d)

Prepared according to the general procedure.

Color and State: Colorless oil.; $[\alpha]_D^{20}$ +15.4 (c = 1.0, $CHCl_3$).

^{1}H NMR (500 MHz, $CDCl_3$) δ 5.86 (ddd, $J = 17.1$, 10.7, 6.3 Hz, 1H), 5.27 (d, $J = 17.1$ Hz, 1H), 5.15 (d, $J = 10.5$ Hz, 1H), 5.12 (app. q, $J = 6.3$ Hz, 1H), 1.48 (s, 9H), 1.35 (d, $J = 6.6$ Hz, 3H).

IR (Neat) 2982 (w), 2935 (w), 1736 (s), 1647 (w), 1369 (m), 1273 (s), 1252 (vs), 1162 (s) cm^{-1}.

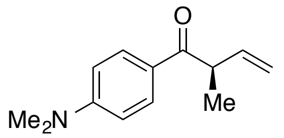

(*R*)-1-(4-(Dimethylamino)phenyl)-2-methylbut-3-en-1-one ((*R*)-3d). Lithium bis(trimethylsilyl)amide (12.6 mL, 12.6 mmol; 1M solution in THF) was added dropwise to a stirred solution of 2-(*tert*-butyldimethylsilyloxy)-2-(4-(dimethylamino)phenyl)acetonitrile **2a** (2.64 g, 9.10 mmol) and lithium chloride (594 mg, 14.0 mmol) in anhydrous tetrahydrofuran (22.5 mL) at –10 °C under an atmosphere of argon. The anion was allowed to form over *ca.* 30 minutes, resulting in a dark green homogeneous solution. In a separate flask, $[RhCl(COD)]_2$ (86.0 mg, 0.175 mmol) and tris(2,2,2-trifluoroethyl) phosphite (154 μL, 0.70 mmol) were dissolved in anhydrous tetrahydrofuran (12.5 mL) at room temperature under an atmosphere of argon. The mixture was stirred for *ca.* 5 minutes, resulting in a light yellow homogeneous solution, and then cooled to –10 °C. The catalyst solution was then added to the anion *via* Teflon® cannula, followed immediately by the addition of (*R*)-but-3-en-2-yl *tert*-butyl carbonate (*R*)**-1e** (1.21 g, 7.0 mmol). The mixture was allowed to stir for *ca.* 16 hours and then cooled to –40 °C (dry ice/acetonitrile bath). TBAF (35.0 mL, 35.0 mmol; 1M solution in THF) was then added dropwise and the solution was allowed to stir for *ca.* 1 hour at –40 °C. The reaction mixture was

quenched with saturated aqueous ammonium chloride solution (20 mL), allowed to warm to room temperature and then partitioned between diethyl ether and saturated aqueous ammonium chloride solution. The combined organic layers were dried (anhyd. $MgSO_4$), filtered and concentrated *in vacuo* to give the crude product. Purification by flash column chromatography (silica gel, eluting with 10-30% diethyl ether/hexane) afforded the *α-ternary ketone* **7** (1.15 g, 81%) as a white solid: **mp** = 46-48 °C; *b:l* ≥19:1; $[\alpha]_D^{20}$ –17.4 (*c* = 1.0, $CHCl_3$); Chiral HPLC analysis (CHIRALPAK AS-H column), 95:5 hexane/isopropanol at 1.0 mL/min flow rate; t_R (*R*)-enantiomer (major) = 13.4 min., t_R (*S*)-enantiomer (minor) = 14.8 min.; 94% *ee*; 95% *cee*.

^{1}H NMR (500 MHz, $CDCl_3$) δ 7.93-7.90 (m, 2H), 6.67-6.64 (m, 2H), 6.01 (ddd, *J* = 17.5, 10.2, 7.6 Hz, 1H), 5.15 (app. dt, *J* = 17.2, 1.3 Hz, 1H), 5.09 (ddd, *J* = 10.2, 1.3, 1.0 Hz, 1H), 4.11 (app. pentet of triplets, *J* = 6.8, 1.0 Hz, 1H), 3.06 (s, 6H), 1.31 (d, *J* = 6.8 Hz, 3H).

IR (Neat) 3083 (w), 3052 (w), 2975 (w), 2931 (w), 2870 (w), 2818 (w), 1659 (m), 1593 (vs), 1551 (m), 1527 (m), 1368 (s), 1188 (s), 1168 (s), 944 (m), 827 (m) cm^{-1}.

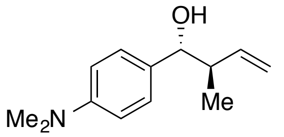

(1*R*,2*R*)-1-(4-(Dimethylamino)phenyl)-2-methylbut-3-en-1-ol (7). (*S*)-CBS (1.39 g, 5.0 mmol) was dissolved in anhydrous tetrahydrofuran (12.5 mL) and cooled with stirring to –30 °C under an atmosphere of argon. Borane dimethylsulfide complex (0.95 mL, 10 mmol) was added to the solution and the mixture was stirred for *ca.* 30 minutes. A solution of (*R*)-1-(4-(dimethylamino)phenyl)-2-methylbut-3-en-1-one (*R*)**-3d** (1.02 g, 5.0 mmol) in anhydrous tetrahydrofuran (12.5 mL) was then added dropwise and the reaction mixture was stirred for *ca.* 16 hours. The reaction mixture was quenched with methanol (5 mL) and partitioned between ethyl acetate and saturated aqueous sodium chloride solution. The combined organic layers were the sequentially washed with a saturated aqueous sodium bicarbonate and sodium chloride, dried (anhyd. $MgSO_4$), filtered and concentrated *in vacuo* to give the crude product. Purification by flash column chromatography (silica gel, eluting with 20-30% ethyl acetate/hexane) afforded the *alcohol* **8** (854 mg, 83%) as a pale yellow oil: *dr* ≥19:1; $[\alpha]_D^{20}$ +91.2 (*c* = 1.0, $CHCl_3$).

^{1}H NMR (500 MHz, $CDCl_3$) δ 7.22-7.19 (m, 2H), 6.73-6.70 (m, 2H), 5.85 (ddd, *J* = 17.4, 10.1, 8.1 Hz, 1H), 5.20 (app. dt, *J* = 17.3, 0.9 Hz, 1H), 5.16 (dd, *J* = 10.2, 1.8 Hz, 1H), 4.26 (dd, *J* = 8.2, 1.7 Hz, 1H), 2.95 (s, 6H), 2.48 (sextet, *J* = 7.4 Hz, 1H), 2.00 (br. d, *J* = 2.4 Hz, 1H), 0.85 (d, *J* = 6.8 Hz, 3H).

^{13}C NMR (125 MHz, $CDCl_3$) δ 150.35 (e), 141.51 (o), 130.54 (e), 127.79 (o), 116.26 (e), 112.49 (o), 77.88 (o), 46.28 (o), 40.76 (o), 16.79 (o).

IR (Neat) 3397 (br, w), 3076 (w), 2962 (w), 2879 (m), 2800 (w), 1614 (s), 1521 (s), 1345 (s), 1187 (m), 1162 (s), 1003 (s), 947 (m), 814 (s) cm^{-1}.

HRMS (ESI, $[M+H]^+$) calcd for $C_{13}H_{20}NO$ 206.1539, found 206.1539.

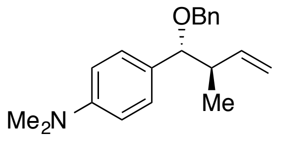

4-((1*R*,2*R*)-1-(Benzyloxy)-2-methylbut-3-enyl)-*N*,*N*-dimethylaniline (8a). A solution of (1*R*,2*R*)-1-(4-(Dimethylamino)phenyl)-2-methylbut-3-en-1-ol **7** (115 mg, 0.560 mmol) in dimethylformamide (1.4 mL) was added dropwise to a stirred suspension of sodium hydride (25.0 mg, 0.616 mmol; 60% dispersion in mineral oil) in dimethylformamide (4.2 mL) at 0 °C under an atmosphere of argon. The mixture was stirred for *ca.* 15 minutes before benzyl bromide (73.0 μL, 0.616 mmol) was added and the solution was allowed to warm to room temperature and stirred for *ca.* 16 hours. The reaction mixture was cooled to 0 °C and quenched with saturated aqueous ammonium chloride solution (5 mL), allowed to warm to room temperature and then partitioned between diethyl ether and saturated aqueous ammonium chloride solution. The combined organic layers were washed with water, dried (anhyd. $MgSO_4$), filtered and concentrated *in vacuo* to give the crude product. Purification by flash column chromatography (silica gel, eluting with 5-15% diethyl ether/hexane) afforded the *benzyl ether* **8a** (136 mg, 82%) as a pale yellow oil: $[\alpha]_D^{20}$ +99.7 (c = 1.0, $CHCl_3$).

^{1}H NMR (500 MHz, $CDCl_3$) δ 7.35-7.29 (m, 4H), 7.27-7.23 (m, 1H), 7.18-7.15 (m, 2H), 6.74-6.72 (m, 2H), 5.96 (ddd, J = 17.1, 10.6, 7.3 Hz, 1H), 5.04 (ddd, J = 17.1, 1.8, 1.3 Hz, 1H), 5.03 (ddd, J = 10.6, 1.8, 1.0 Hz, 1H), 4.46 (d, A of AB, J_{AB} = 12.3 Hz, 1H), 4.20 (d, B of AB, J_{AB} = 12.1 Hz, 1H), 4.00 (d, J = 7.6 Hz, 1H), 2.97 (s, 6H), 2.56 (app. sextet of triplets, J = 7.1, 1.1 Hz, 1H), 0.83 (d, J = 6.9 Hz, 3H).

^{13}C NMR (125 MHz, $CDCl_3$) δ 150.22 (e), 142.09 (o), 139.20 (e), 128.70 (o), 128.35 (e), 128.30 (o), 127.79 (o), 127.33 (o), 113.98 (e), 112.24 (o), 85.29 (o), 70.09 (e), 44.45 (o), 40.72 (o), 16.62 (o).

IR (Neat) 3065 (w), 3027 (w), 2962 (w), 2929 (w), 2863 (w), 2800 (w), 1613 (s), 1521 (s), 1453 (m), 1346 (m), 1186 (m), 1164 (m), 1088 (m), 1065 (s), 947 (m), 814 (s), 696 (vs) cm^{-1}.

HRMS (ESI, $[M+H]^+$) calcd for $C_{20}H_{26}NO$ 296.2009, found 296.2003.

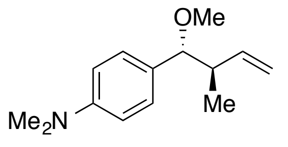

4-((1*R*,2*R*)-1-Methoxy-2-methylbut-3-enyl)-*N*,*N*-dimethylaniline (8b). A solution of (1*R*,2*R*)-1-(4-(Dimethylamino)phenyl)-2-methylbut-3-en-1-ol **7** (123 mg, 0.60 mmol) in dimethylformamide (1.5 mL) was added dropwise to a stirred suspension of sodium hydride (26.0 mg, 0.66 mmol; 60% dispersion in

mineral oil) in dimethylformamide (4.5 mL) at 0 °C under an atmosphere of argon. The mixture was stirred for *ca.* 15 minutes before iodomethane (41.0 µL, 0.66 mmol) was added and the solution was allowed to warm to room temperature and stirred for *ca.* 16 hours. The reaction mixture was cooled to 0 °C and quenched with saturated aqueous ammonium chloride solution (5 mL), allowed to warm to room temperature and then partitioned between diethyl ether and saturated aqueous ammonium chloride solution. The combined organic layers were washed with water, dried (anhyd. $MgSO_4$), filtered and concentrated *in vacuo* to give the crude product. Purification by flash column chromatography (silica gel, eluting with 5-15% diethyl ether/hexane) afforded the *methyl ether* **8b** (101 mg, 77%) as a colorless oil: $[\alpha]_D^{20}$ +51.4 (*c* = 1.0, $CHCl_3$).

^{1}H NMR (500 MHz, $CDCl_3$) δ 7.14-7.11 (m, 2H), 6.73-6.70 (m, 2H), 5.91 (ddd, *J* = 17.4, 10.2, 7.3 Hz, 1H), 5.05 (ddd, *J* = 17.2, 1.8, 1.3 Hz, 1H), 5.03 (ddd, *J* = 10.4, 1.8, 1.0 Hz, 1H), 3.81 (d, *J* = 7.7 Hz, 1H), 3.16 (s, 3H), 2.96 (s, 6H), 4.00 (sextet, *J* = 7.1 Hz, 1H), 0.82 (d, *J* = 6.9 Hz, 3H).

^{13}C NMR (125 MHz, $CDCl_3$) δ 150.20 (e), 142.06 (o), 128.52 (o), 128.21 (e), 114.00 (e), 112.21 (o), 88.09 (o), 56.68 (o), 44.51 (o), 40.71 (o), 16.68 (o).

IR (Neat) 3079 (w), 2961 (w), 2925 (m), 2853 (m), 2818 (w), 1614 (s), 1521 (s), 1347 (s), 1185 (m), 1164 (m), 1090 (vs), 948 (m), 814 (s) cm^{-1}.

HRMS (ESI, $[M+H]^+$) calcd for $C_{14}H_{22}NO$ 220.1696, found 220.1688.

8. Preparation of Biaryl 9

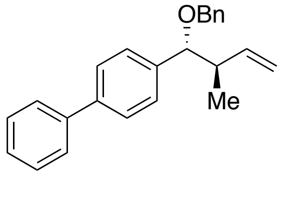

4-((1*R*,2*R*)-1-(Benzyloxy)-2-methylbut-3-enyl)biphenyl (9).[3] Methyl trifluoromethanesulfonate (23.0 µL, 0.21 mmol) was added dropwise to a stirred solution of 4-((1*R*,2*R*)-1-(benzyloxy)-2-methylbut-3-enyl)-*N*,*N*-dimethylaniline **8a** (59.0 mg, 0.20 mmol) in dichloromethane (0.67 mL) at 0 °C under an atmosphere of argon. The resulting mixture was allowed to warm to room temperature and stirred for *ca.* 3 hours. Tetrahydrofuran (1.33 mL) and bis(triphenylphosphine)palladium(II) dichloride (7.0 mg, 10 µmmol) were then added and the flask was evacuated and backfilled with argon (3×). Phenylmagnesium bromide (0.22 mL, 0.22 mmol; 1M solution in THF) was then added dropwise and the light brown, heterogeneous solution was stirred at room temperature for *ca.* 16 hours. The reaction mixture was quenched with water (0.75 mL) and partitioned between ether and a 2M hydrochloric acid solution. The combined organic layers were dried (anhyd. $MgSO_4$), filtered and concentrated *in vacuo* to give the crude product. Purification by flash column chromatography (silica gel, eluting with 1-3%

diethyl ether/hexane) afforded the *biaryl* **9** (51.0 mg, 78%) as a white solid: **mp** = 71-73 °C; $[\alpha]_D^{20}$ +112.3 (*c* = 1.0, $CHCl_3$).

^{1}H NMR (500 MHz, $CDCl_3$) δ 7.65-7.60 (m, 4H), 7.48-7.44 (m, 2H), 7.39-7.33 (m, 7H), 7.31-7.27 (m, 1H), 5.98 (ddd, J = 17.2, 10.5, 7.4 Hz, 1H), 5.08-5.04 (m, 2H), 4.53 (d, A of AB, J_{AB} = 12.1 Hz, 1H), 4.29 (d, B of AB, J_{AB} = 12.1 Hz, 1H), 4.19 (d, J = 7.2 Hz, 1H), 2.63 (sextet, J = 7.0 Hz, 1H), 0.92 (d, J = 6.9 Hz, 3H).

^{13}C NMR (125 MHz, $CDCl_3$) δ 141.32 (o), 141.02 (e), 140.55 (e), 139.81 (e), 138.82 (e), 128.91 (o), 128.40 (o), 128.23 (o), 127.82 (o), 127.54 (o), 127.38 (o), 127.19 (o), 126.99 (o), 114.56 (e), 85.28 (o), 70.64 (e), 44.43 (o), 16.55 (o).

IR (Neat) 3064 (w), 3029 (w), 2964 (w), 2926 (w), 2863 (w), 1600 (w), 1487 (m), 1454 (m), 1089 (m), 1070 (s), 913 (m), 734 (m), 696 (vs) cm^{-1}.

HRMS (ESI, $[M+Na]^+$) calcd for $C_{24}H_{24}ONa$ 351.1719, found 351.1706.

9. Formal Synthesis of Trichostatic Acid

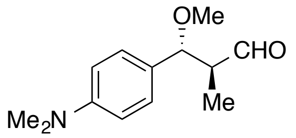

(2*S*,3*R*)-3-(4-(Dimethylamino)phenyl)-3-methoxy-2-methylpropanal (10).[4] 2,6-lutidine (47 μL, 0.40 mmol), osmium tetroxide (31 μL, 4.0 μmmol; 4% aqueous solution) and sodium periodate (171 mg, 0.80 mmol) were added to a stirred solution of 4-((1*R*,2*R*)-1-Methoxy-2-methylbut-3-enyl)-*N*,*N*-dimethylaniline **8b** in dioxane (1.5 mL) and water (0.5 mL) at room temperature under an atmosphere of argon. The resulting hetereogenous mixture was allowed to stir for *ca.* 3 hours before being partitioned between dichloromethane and water. The combined organic layers were washed with saturated aqueous sodium chloride solution, dried (anhyd. $MgSO_4$), filtered and concentrated *in vacuo* to give the crude product. Purification by flash column chromatography (silica gel, eluting with 10-20% ethyl acetate/hexane) afforded the *aldehyde* **10** (34 mg, 78%) as a yellow oil: $[\alpha]_D^{20}$ +90.0 (*c* = 0.76, $CHCl_3$), [lit.[5]: $[\alpha]_D^{20}$ +63.4 (*c* = 0.76, $CHCl_3$)].

^{1}H NMR (500 MHz, $CDCl_3$) δ 9.81 (d, J = 3.1 Hz, 1H), 7.17-7.14 (m, 2H), 6.74-6.71 (m, 2H), 4.16 (d, J = 9.2 Hz, 1H), 3.15 (s, 3H), 2.97 (s, 6H), 2.70 (dqd, A of AMX_3Y, J_{AM} = 9.3 Hz, J_{AX} = 7.1 Hz, J_{AY} = 3.1 Hz, 1H), 0.83 (d, J = 7.1 Hz, 3H).

IR (Neat) 2979 (w), 2928 (w), 2893 (w), 2851 (w), 2819 (m), 2711 (w), 1724 (s), 1670 (w), 1612 (s), 1522 (s), 1446 (m), 1349 (s), 1081 (s), 944 (m), 815 (s) cm^{-1}.

10. References

1. N. Kurono, M. Yamaguchi, K. Suzuki, T. Ohkuma, *J. Org. Chem.* 2005, **70**, 6530.

2. (a) P. A. Evans, S. Oliver, J. Chae, *J. Am. Chem. Soc.* 2012, **134**, 19314. (b) P. A. Evans, S. Oliver, *Org. Lett.* 2013, **15**, 5626. (c) B. W. H. Turnbull, S. Oliver, P. A. Evans, *J. Am. Chem. Soc.* 2015, **137**, 15374.

3. Reeves, J. T.; Fandrick, D. R.; Tan, Z.; Song, J. J.; Lee, H.; Yee, N. K.; Senanayake, C. H. *Org. Lett.* 2010, **12**, 4388.

4. Yu, W.; Mei, Y.; Kang, Y.; Hua, Z.; Jin, Z. *Org. Lett.* 2004, **6**, 3217.

5. Markiewicz, J. T.; Schauer, D. J.; Löfstedt, J.; Corden, S. J.; Wiest, O.; Helquist, P. *J. Org. Chem.* 2010, **75**, 2061.

11. Proton and Carbon NMR Spectra

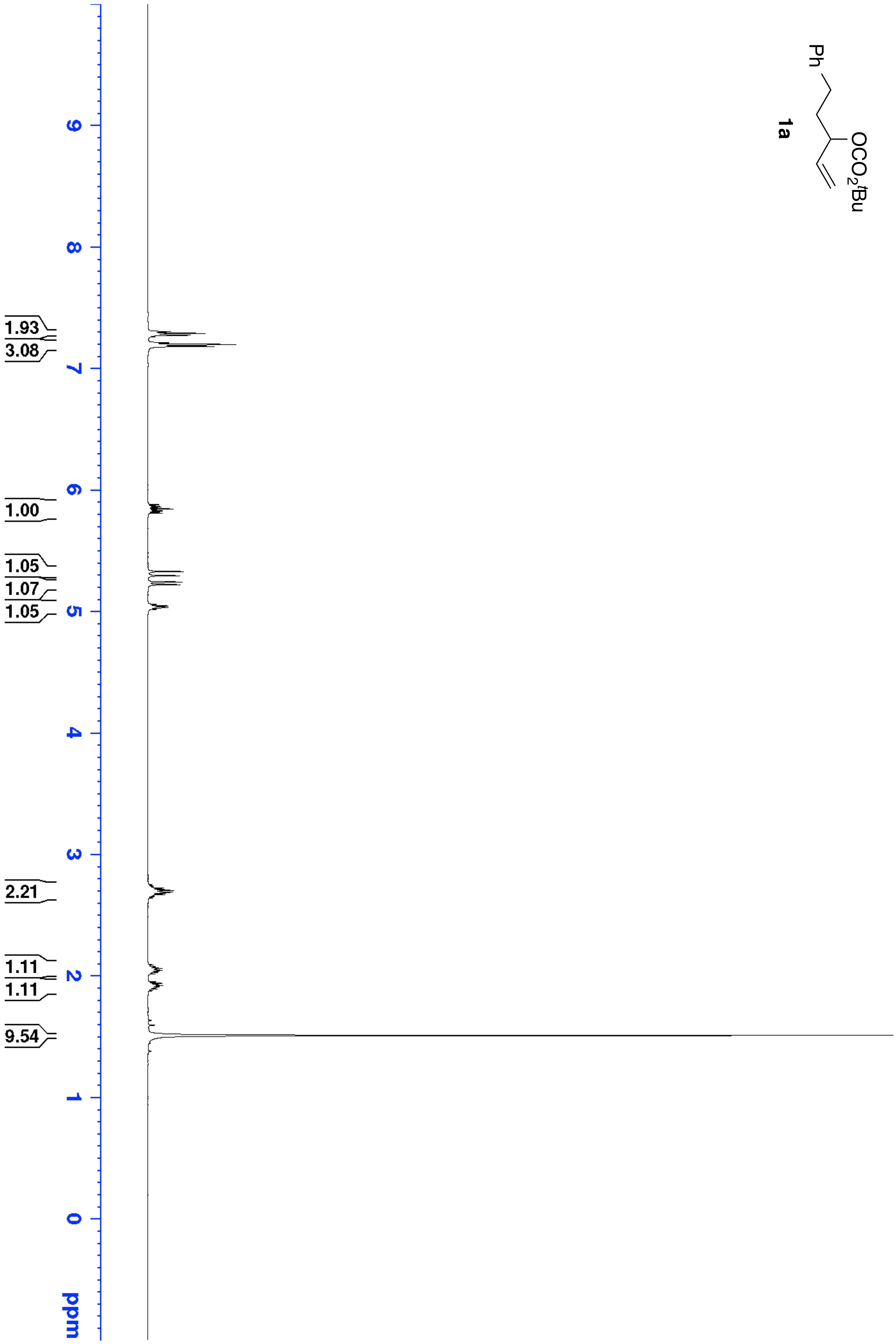

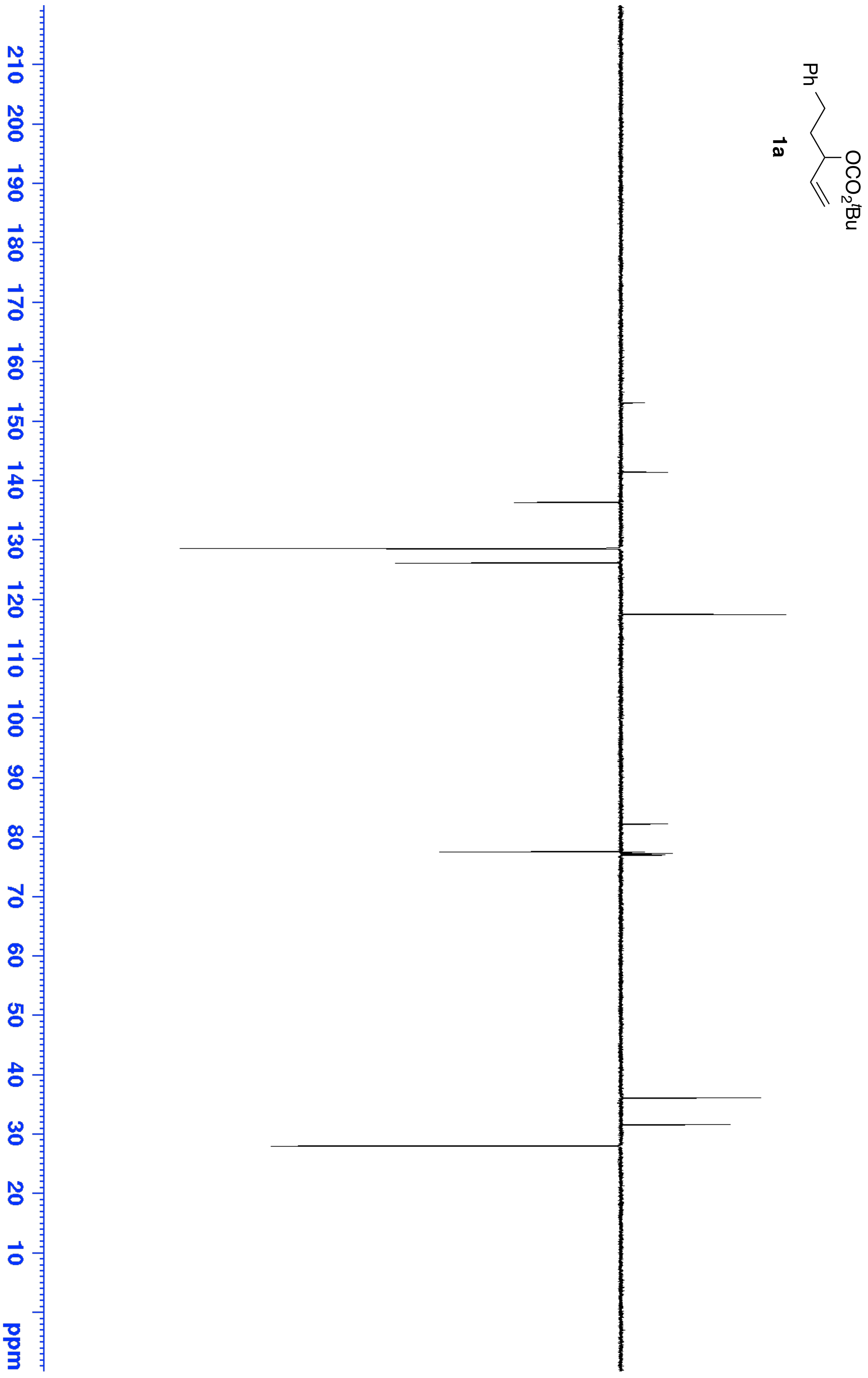

Ph
OCO2tBu
1a
210 200 190 180 170 160 150 140 130 120 110 100 90 80 70 60 50 40 30 20 10 ppm

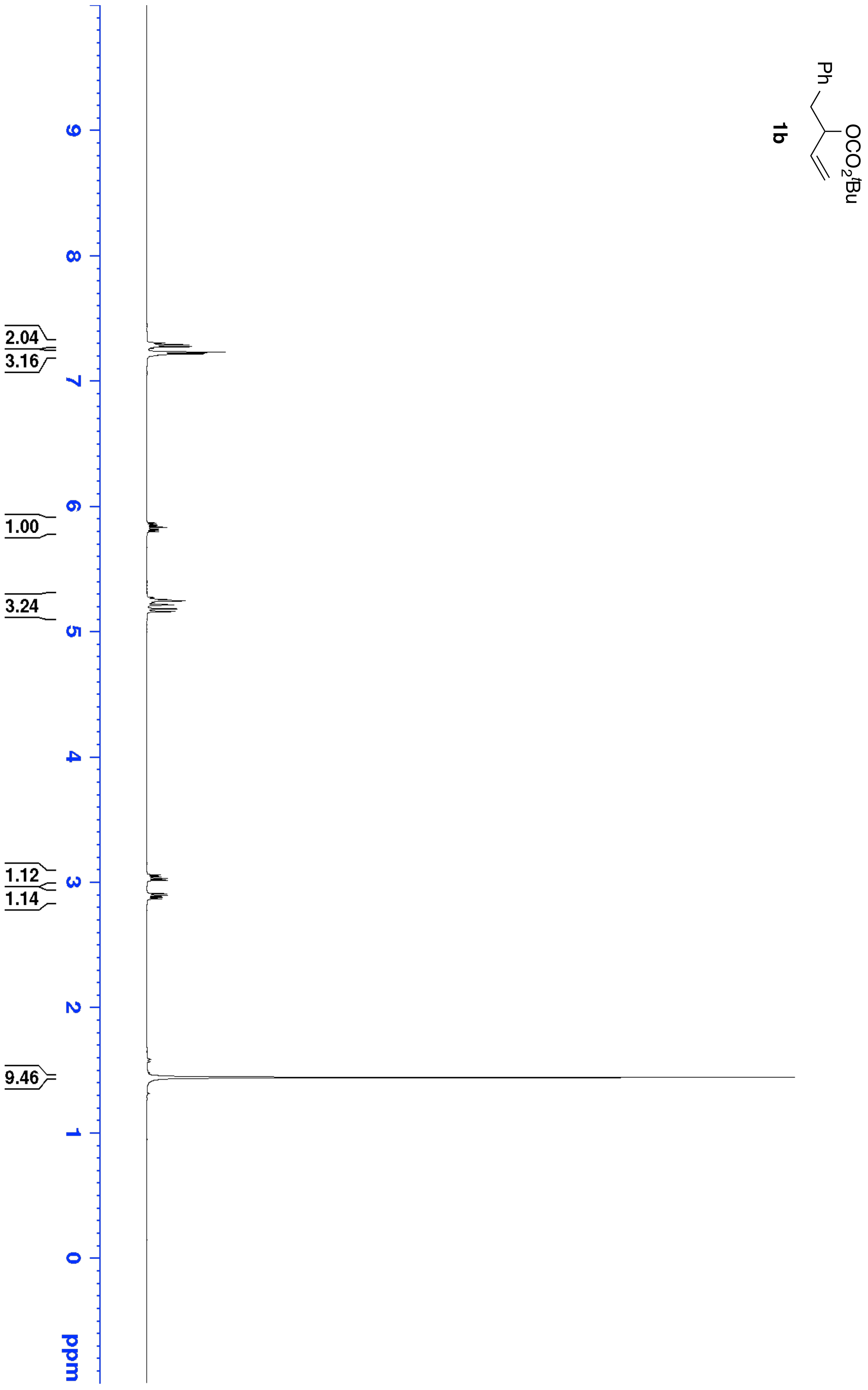
Ph
OCO2tBu
1b
2.04
3.16
1.00
3.24
1.12
1.14
9.46
9
8
7
6
5
4
3
2
1
0
ppm

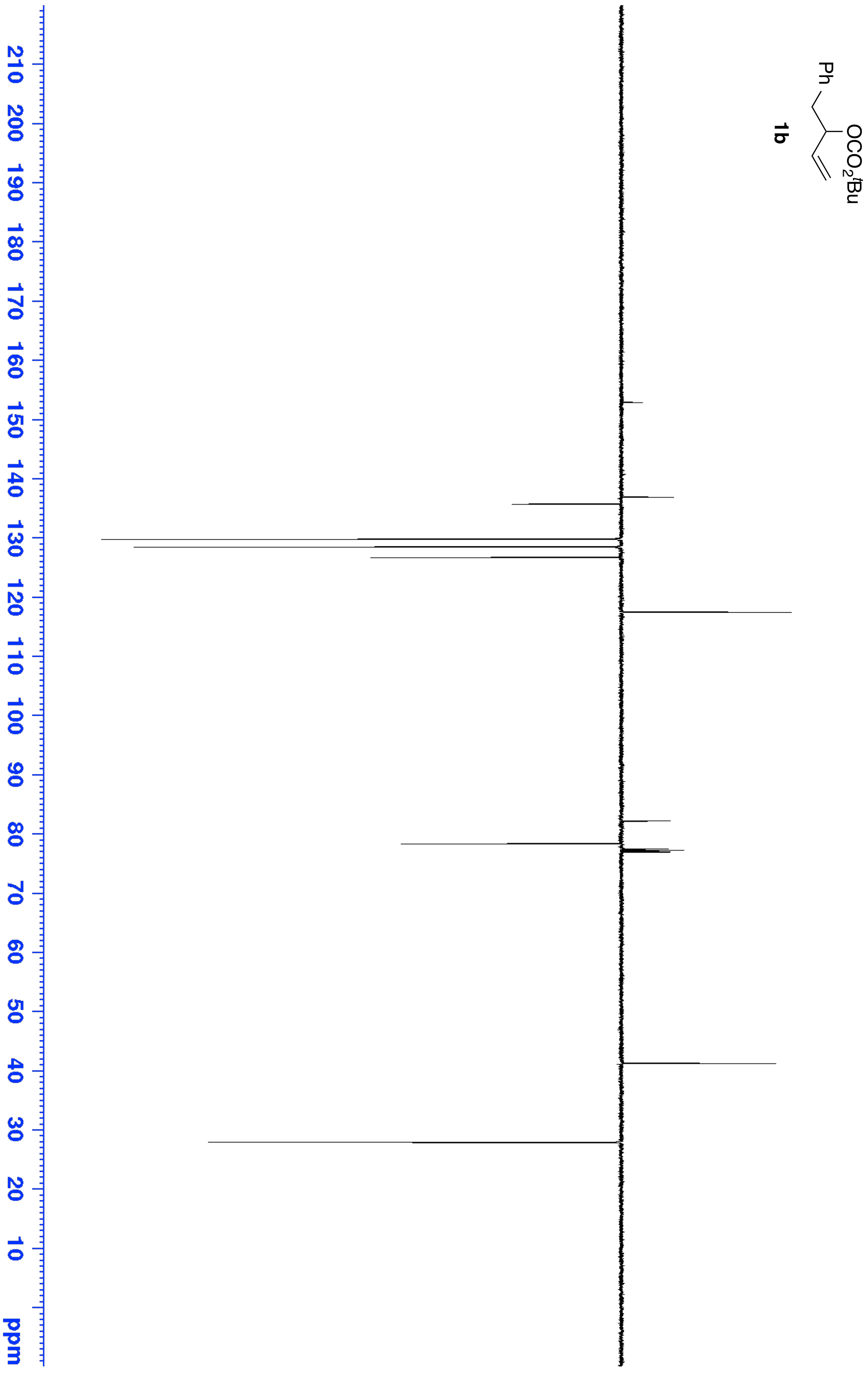
Ph
OCO2tBu
1b
210 200 190 180 170 160 150 140 130 120 110 100 90 80 70 60 50 40 30 20 10 ppm

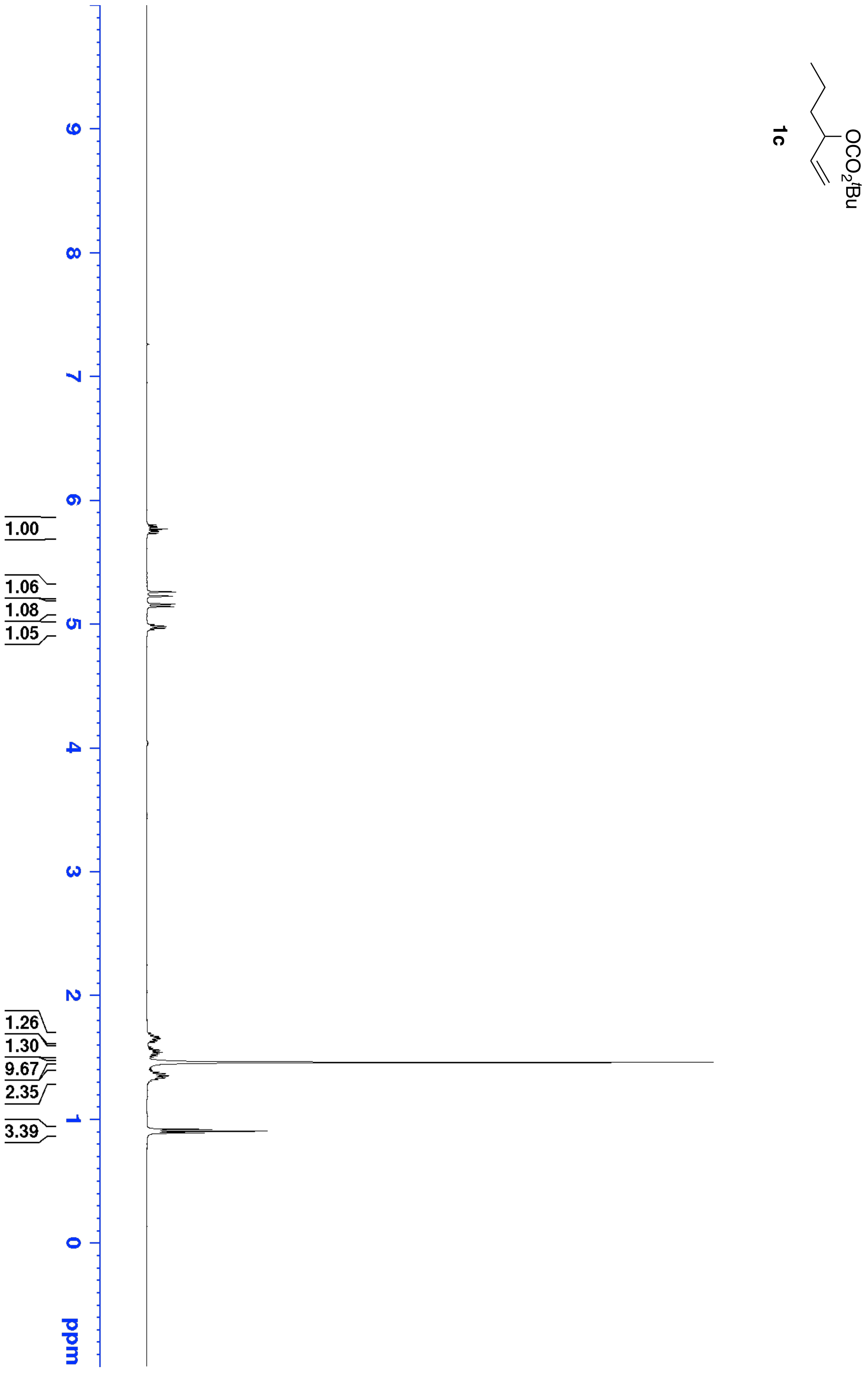

OCO2tBu
1c
1.00
1.06
1.08
1.05
1.26
1.30
9.67
2.35
3.39
9
8
7
6
5
4
3
2
1
0
ppm

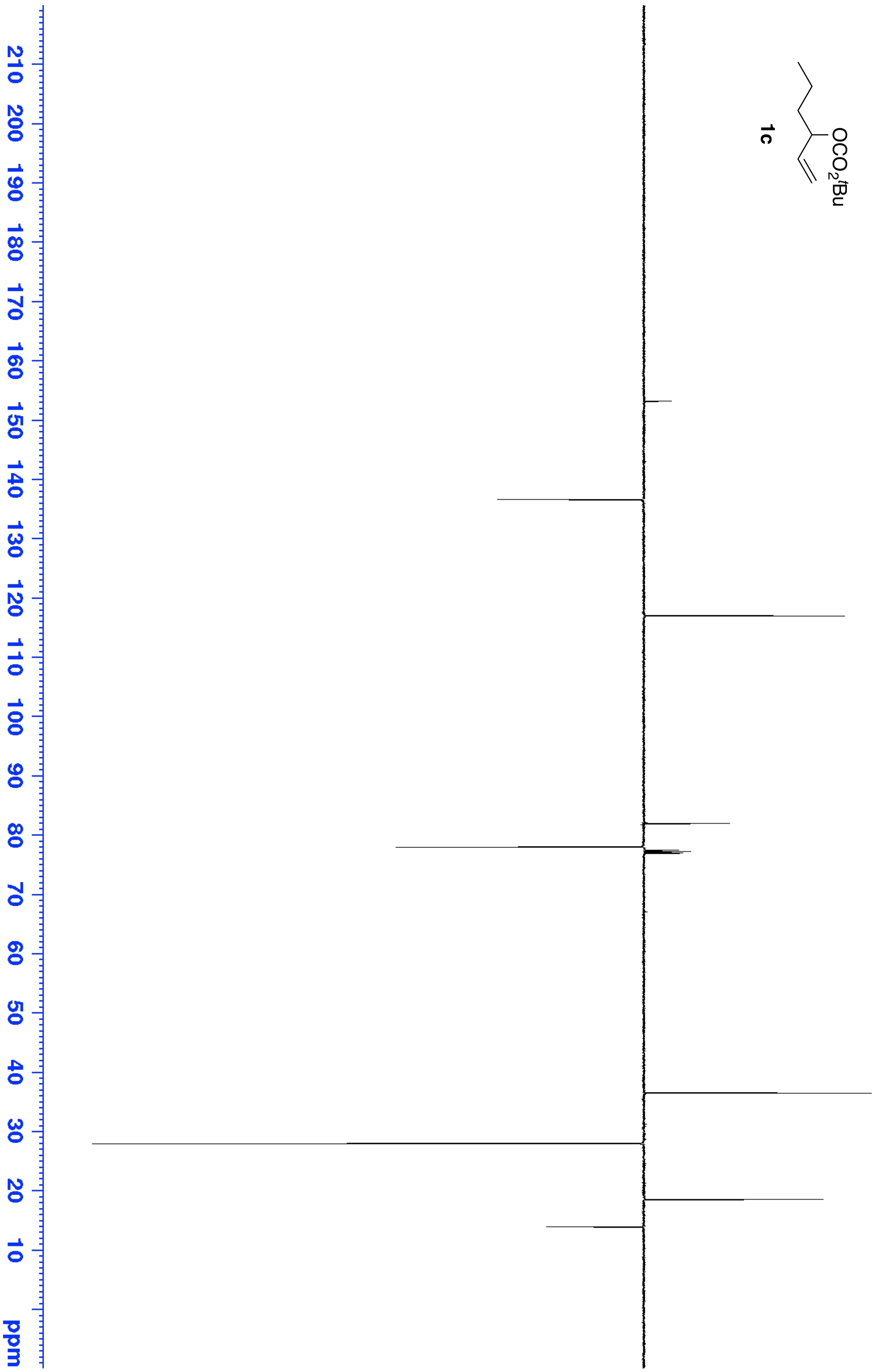

OCO$_2$^{t}Bu
1c
210 200 190 180 170 160 150 140 130 120 110 100 90 80 70 60 50 40 30 20 10 ppm

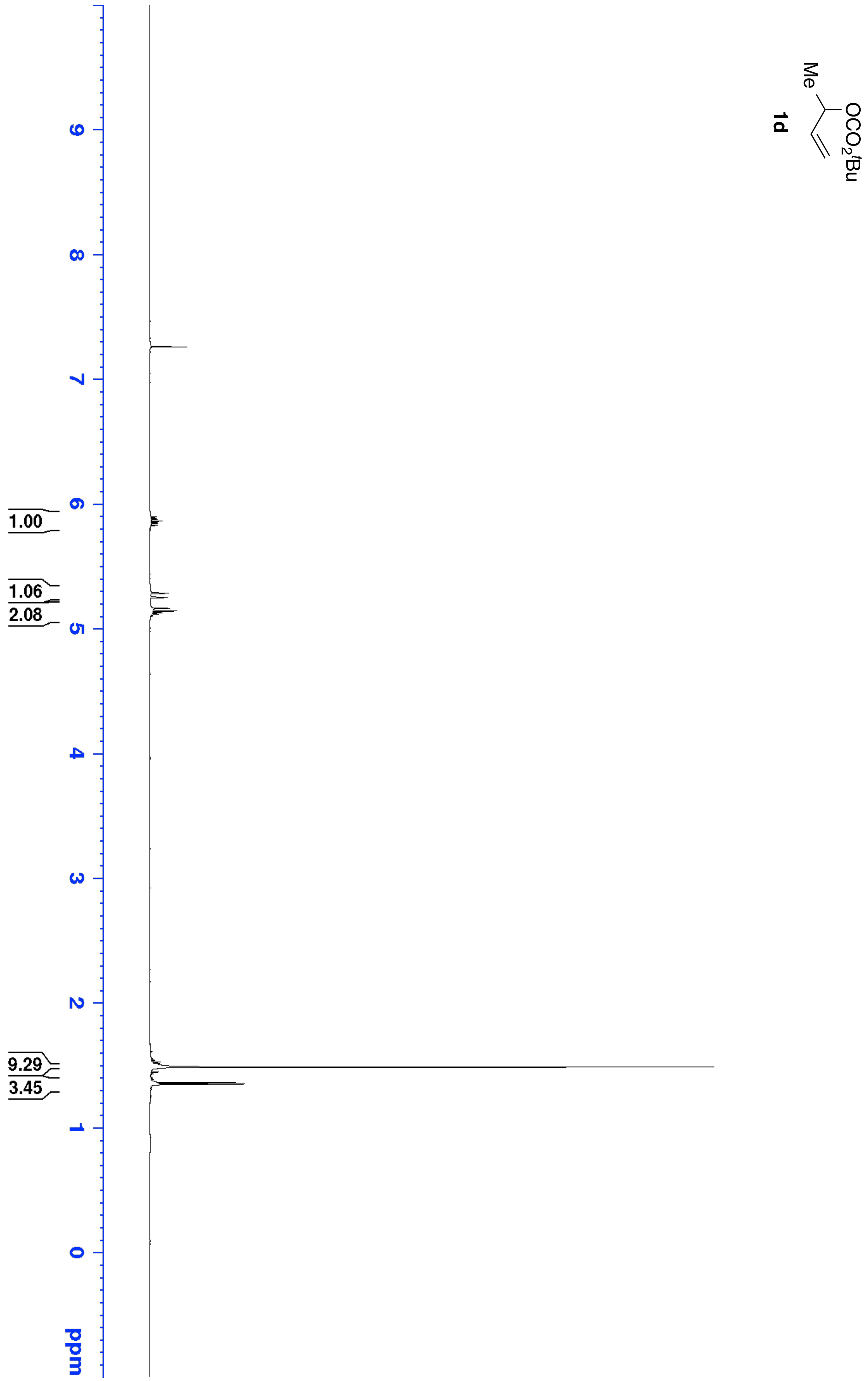
OCO2tBu
Me
1d
1.00
1.06
2.08
9.29
3.45
9
8
7
6
5
4
3
2
1
0
ppm

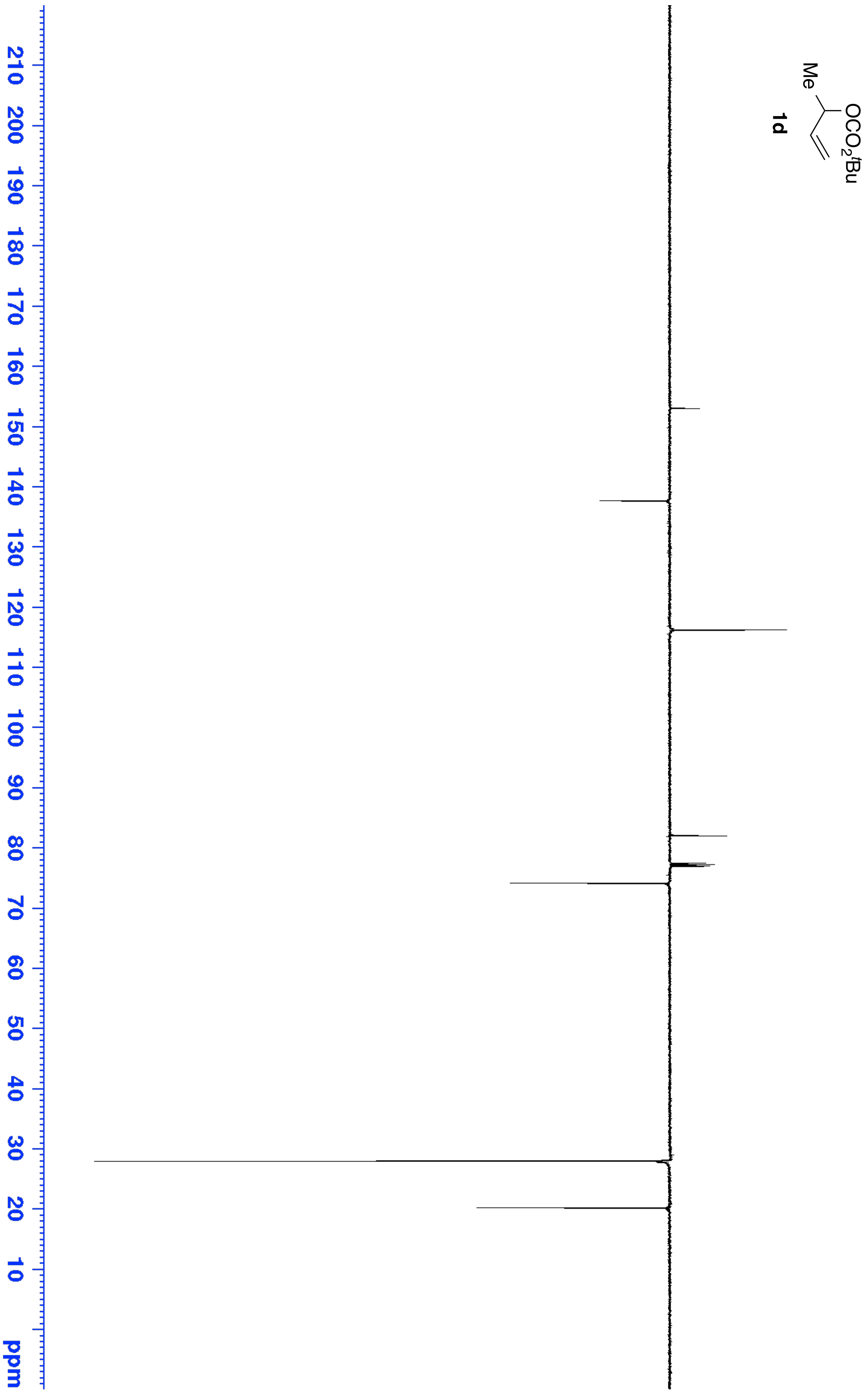
OCO2tBu
Me
1d
210 200 190 180 170 160 150 140 130 120 110 100 90 80 70 60 50 40 30 20 10 ppm

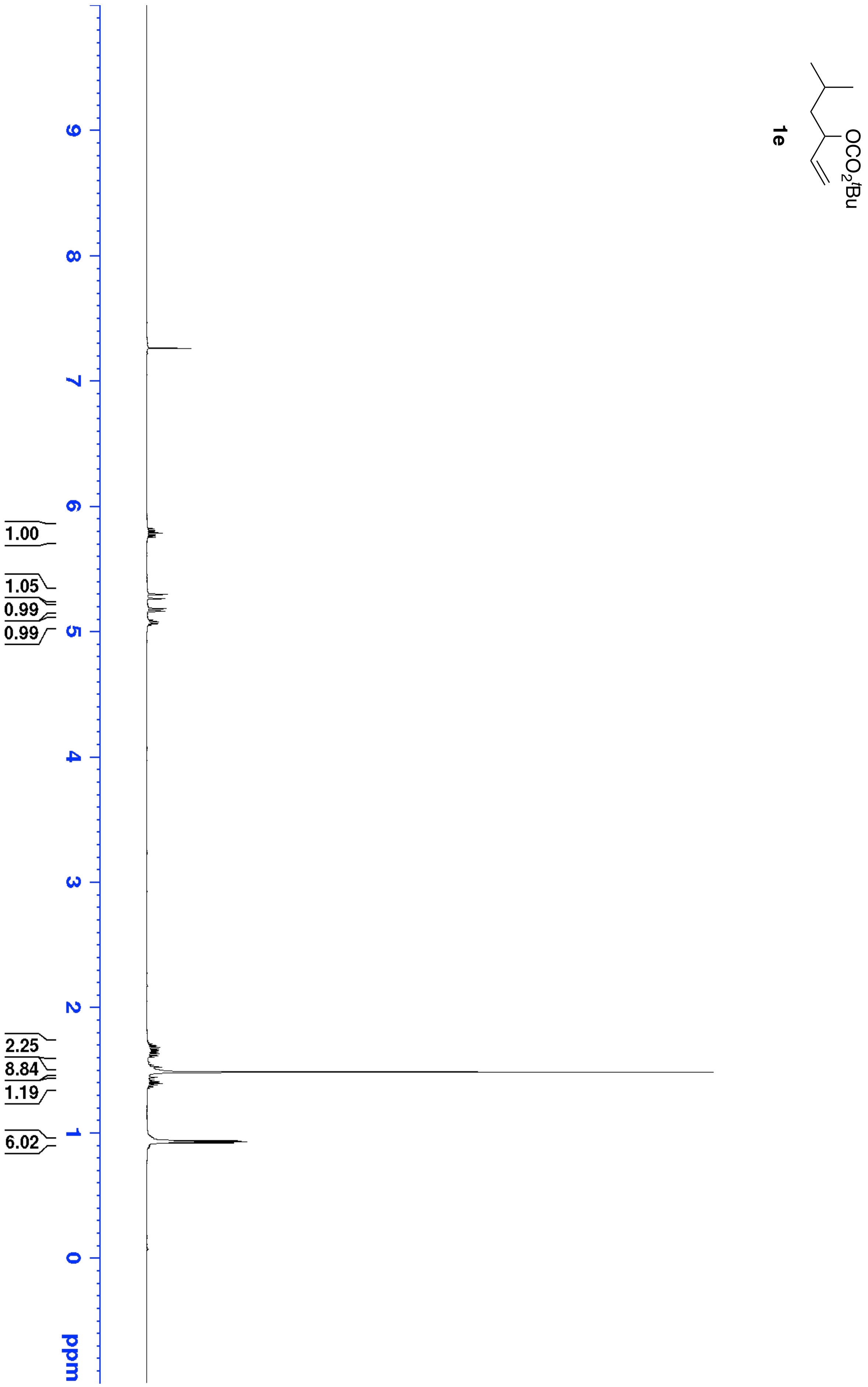
OCO2tBu
1e
1.00
1.05
0.99
0.99
2.25
8.84
1.19
6.02
9
8
7
6
5
4
3
2
1
0
ppm

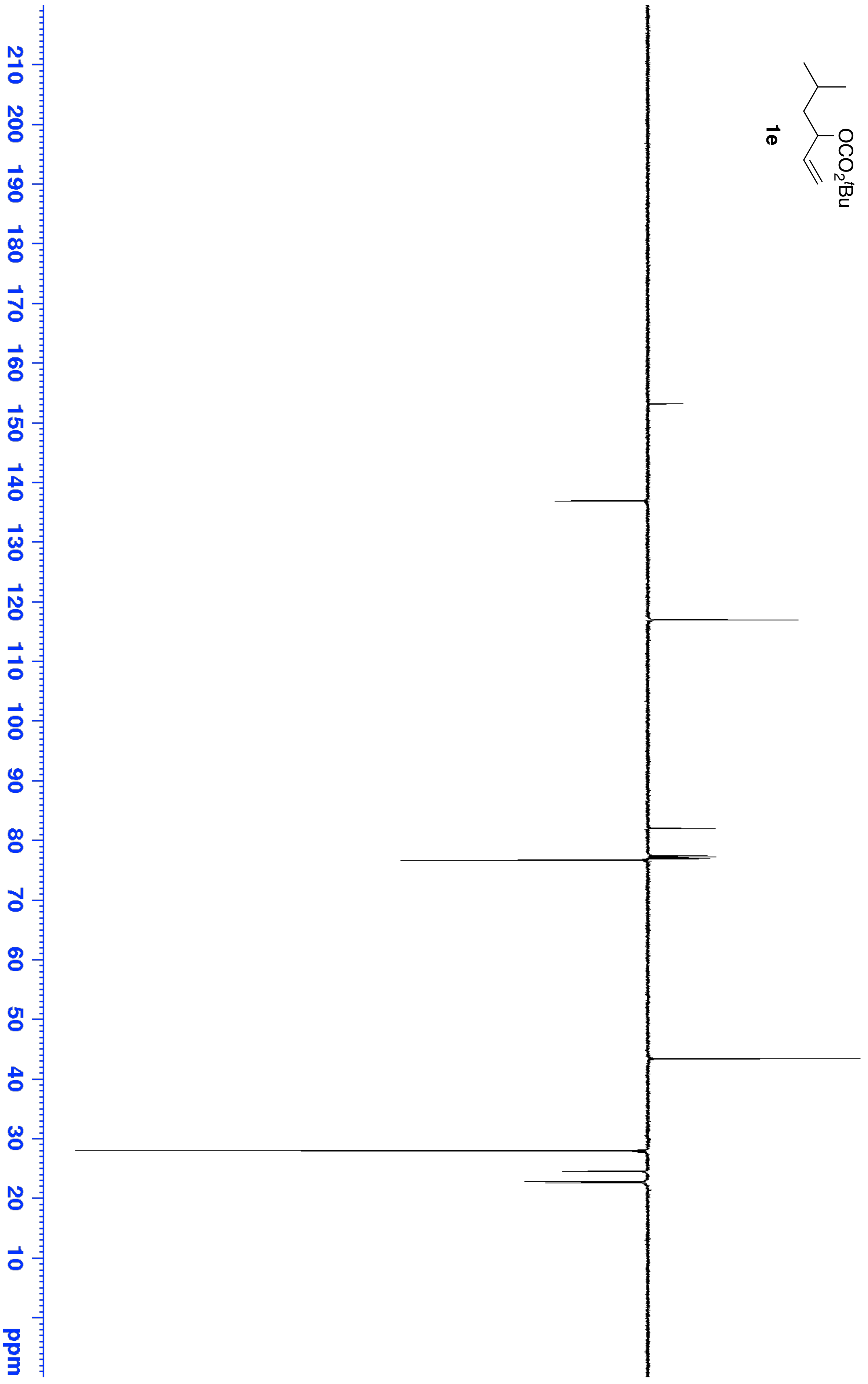
OCO2tBu
1e
210 200 190 180 170 160 150 140 130 120 110 100 90 80 70 60 50 40 30 20 10 ppm

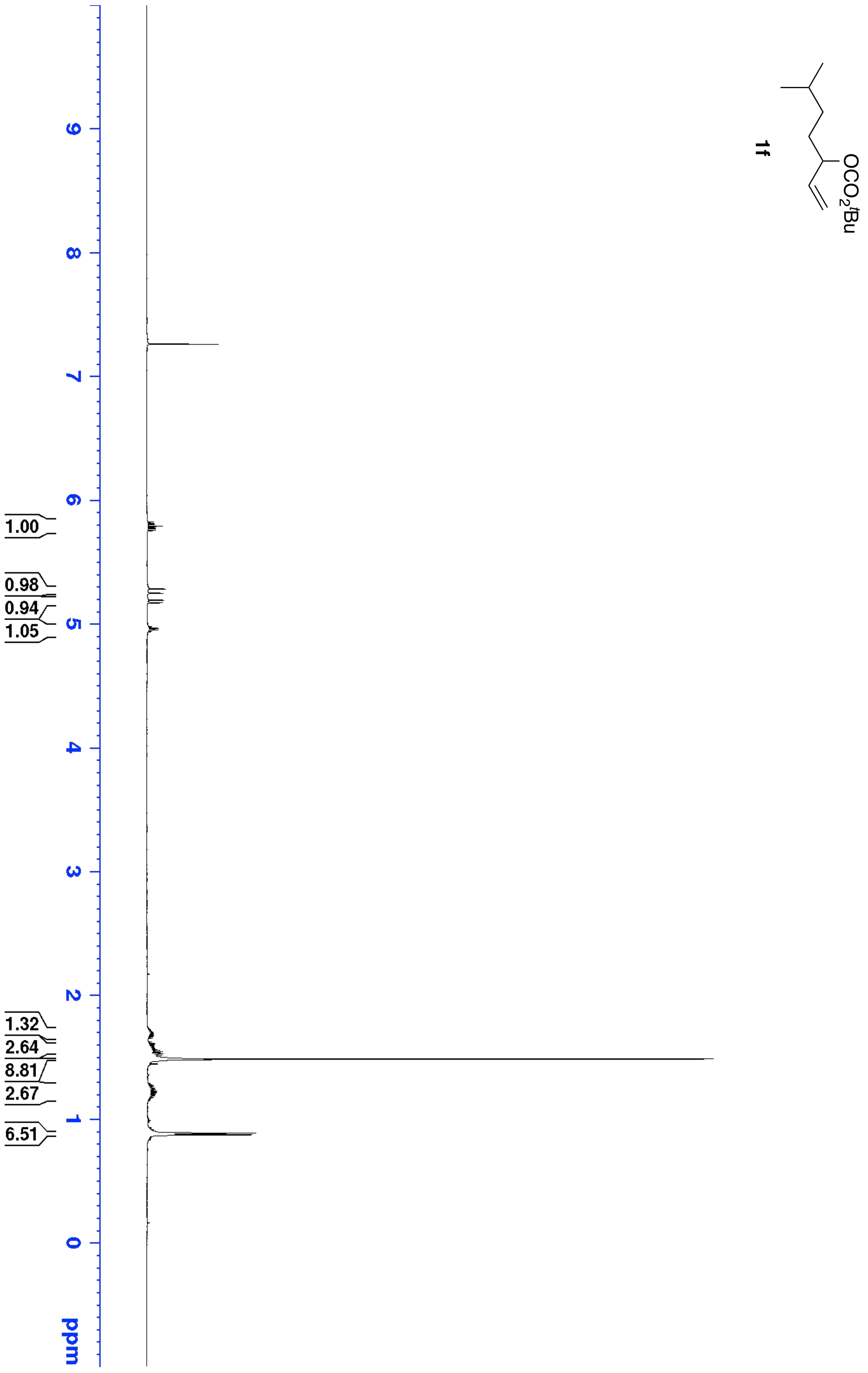
OCO2tBu
1f
1.00
0.98
0.94
1.05
1.32
2.64
8.81
2.67
6.51
9
8
7
6
5
4
3
2
1
0
ppm

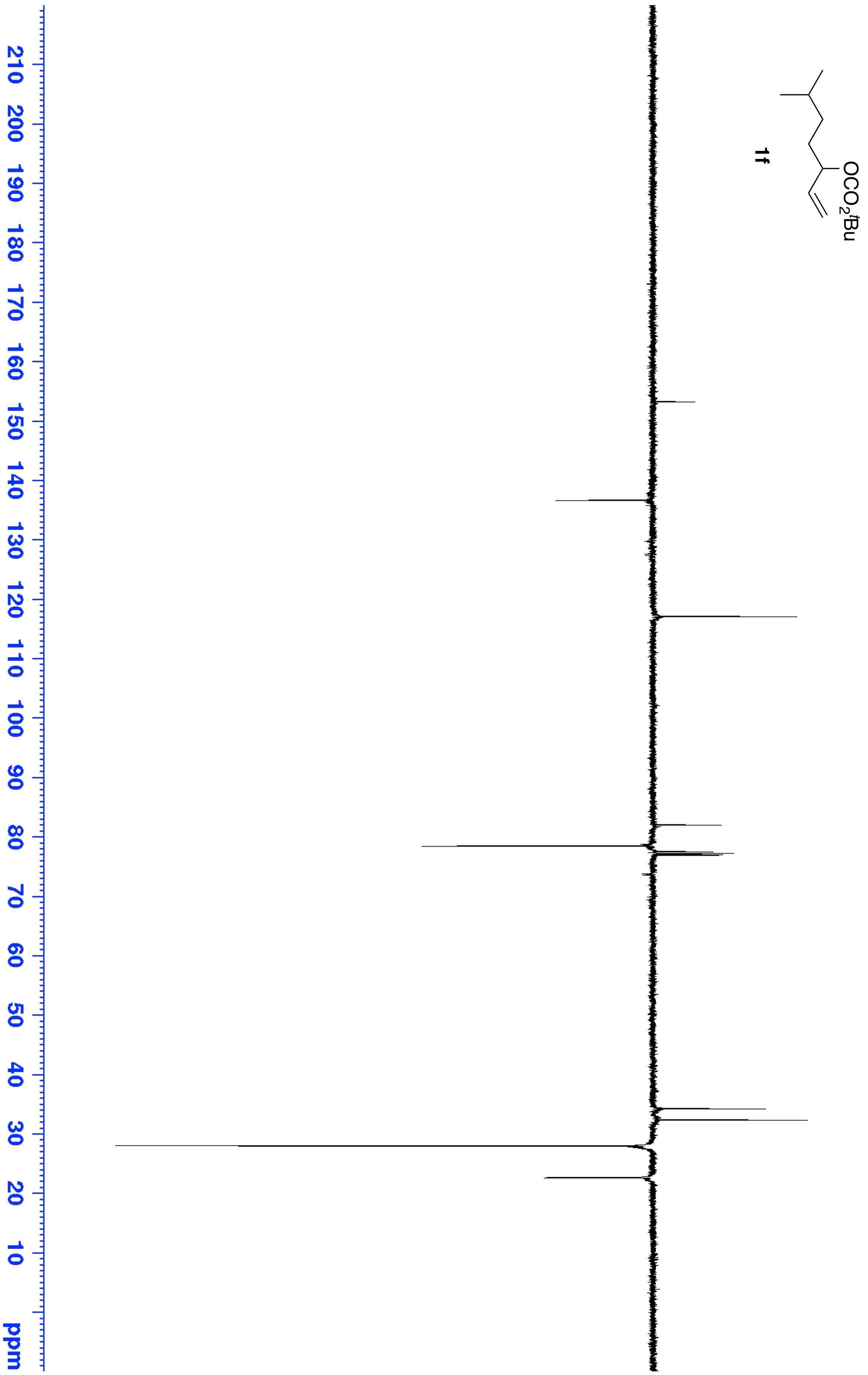

OCO2tBu
1f
210
200
190
180
170
160
150
140
130
120
110
100
90
80
70
60
50
40
30
20
10
ppm

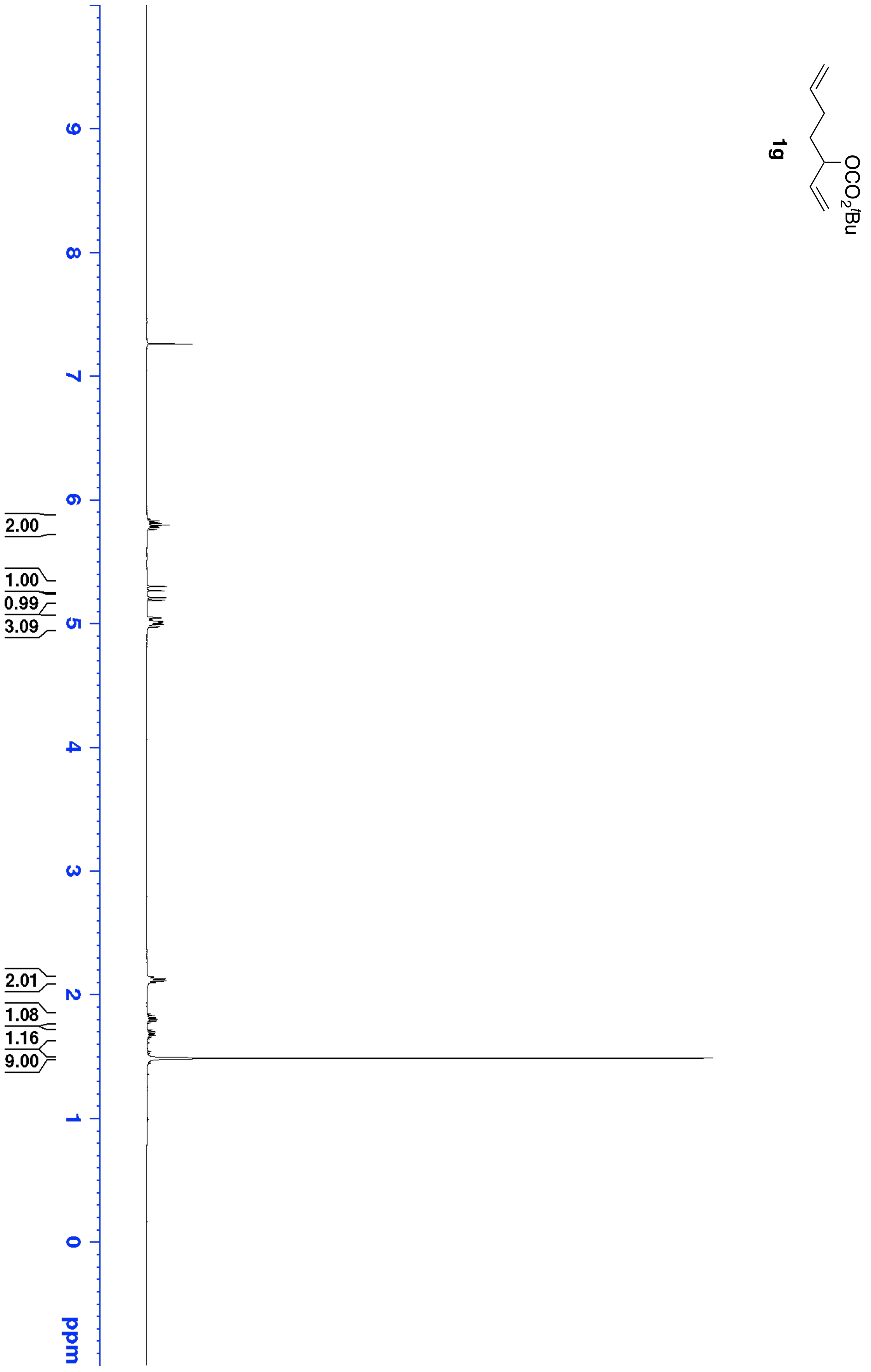
OCO2tBu
1g
2.00
1.00
0.99
3.09
2.01
1.08
1.16
9.00
9
8
7
6
5
4
3
2
1
0
ppm

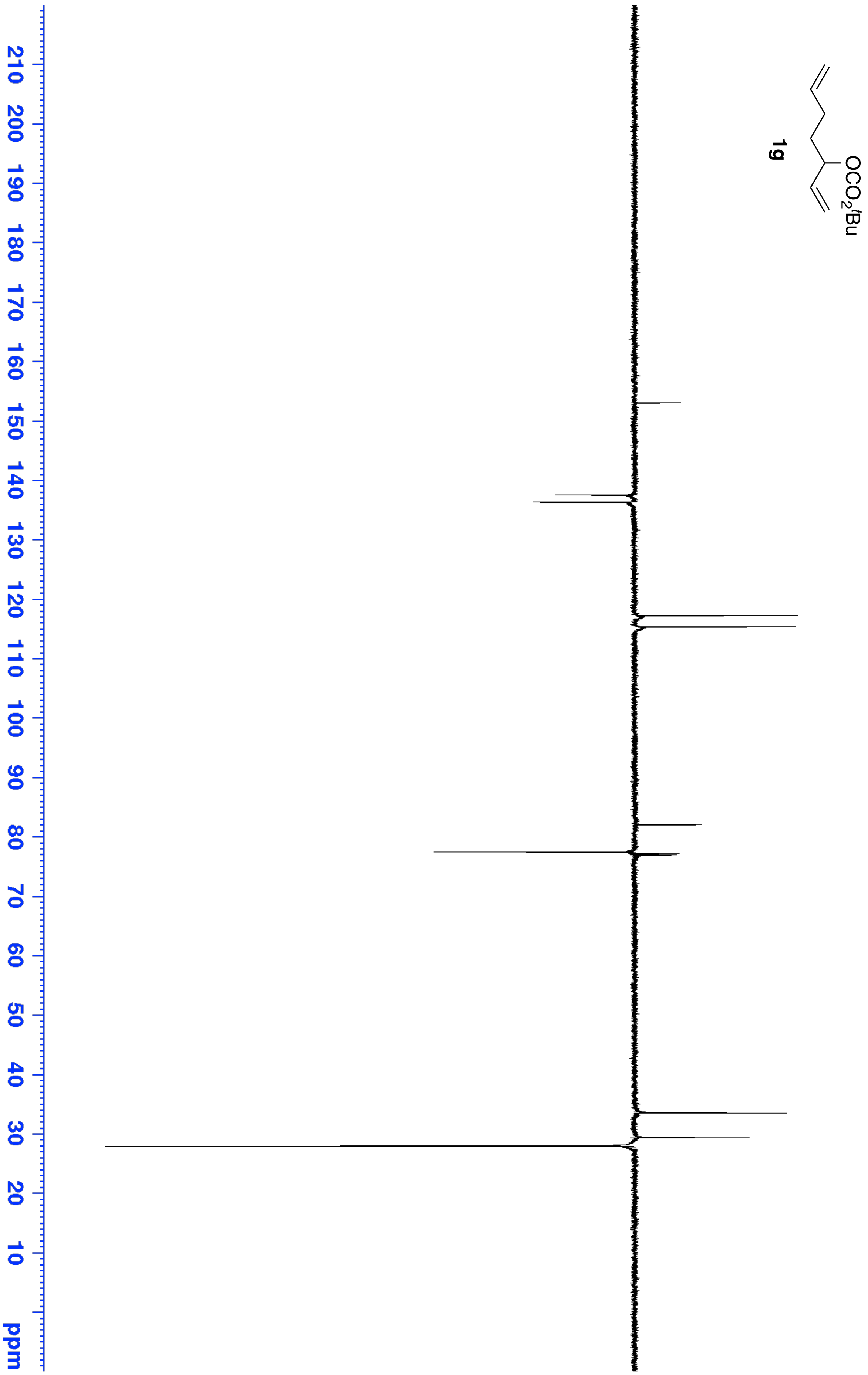
OCO2tBu
1g
210 200 190 180 170 160 150 140 130 120 110 100 90 80 70 60 50 40 30 20 10 ppm

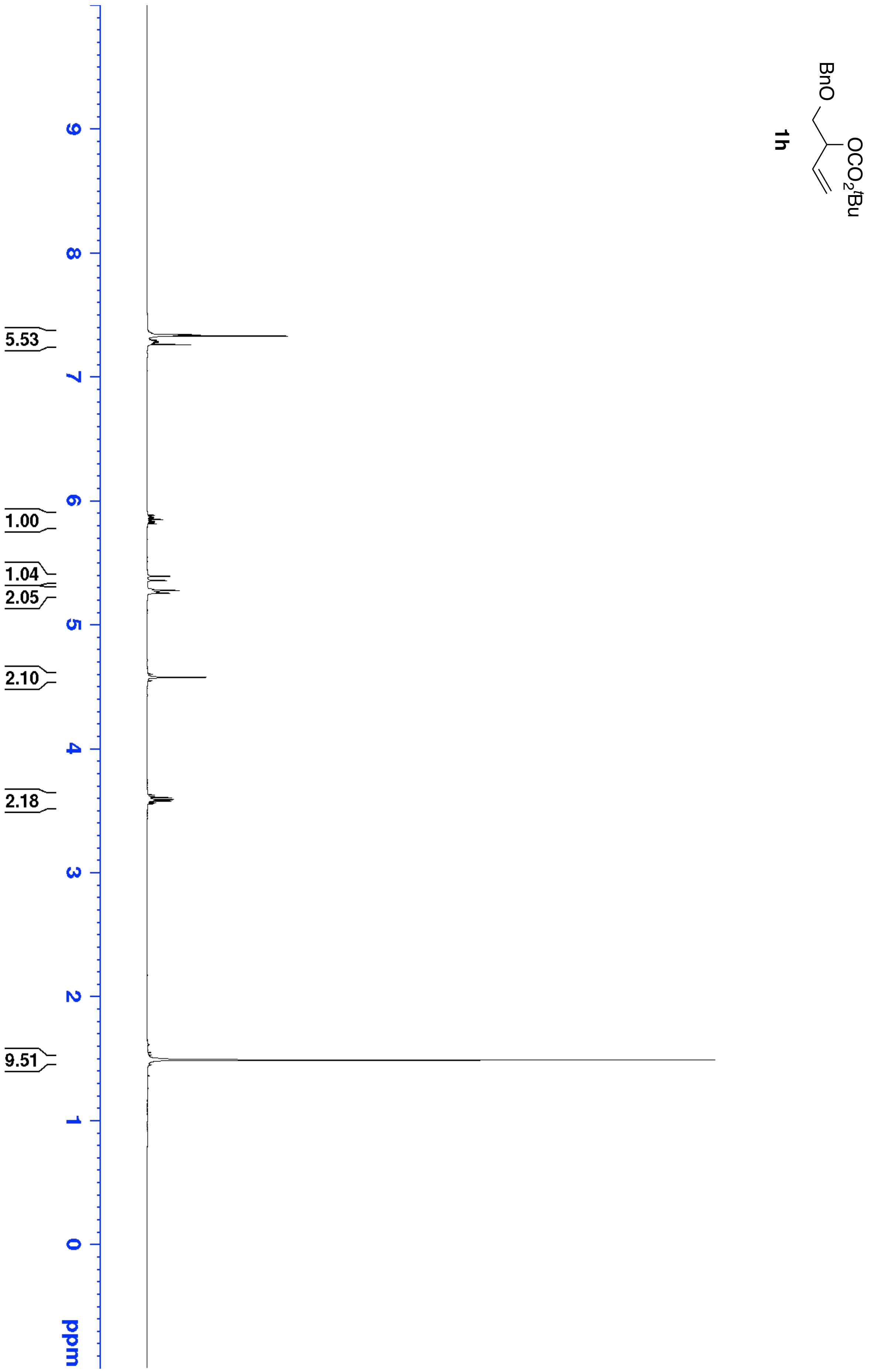

BnO
OCO2tBu
1h
5.53
1.00
1.04
2.05
2.10
2.18
9.51
9
8
7
6
5
4
3
2
1
0
ppm

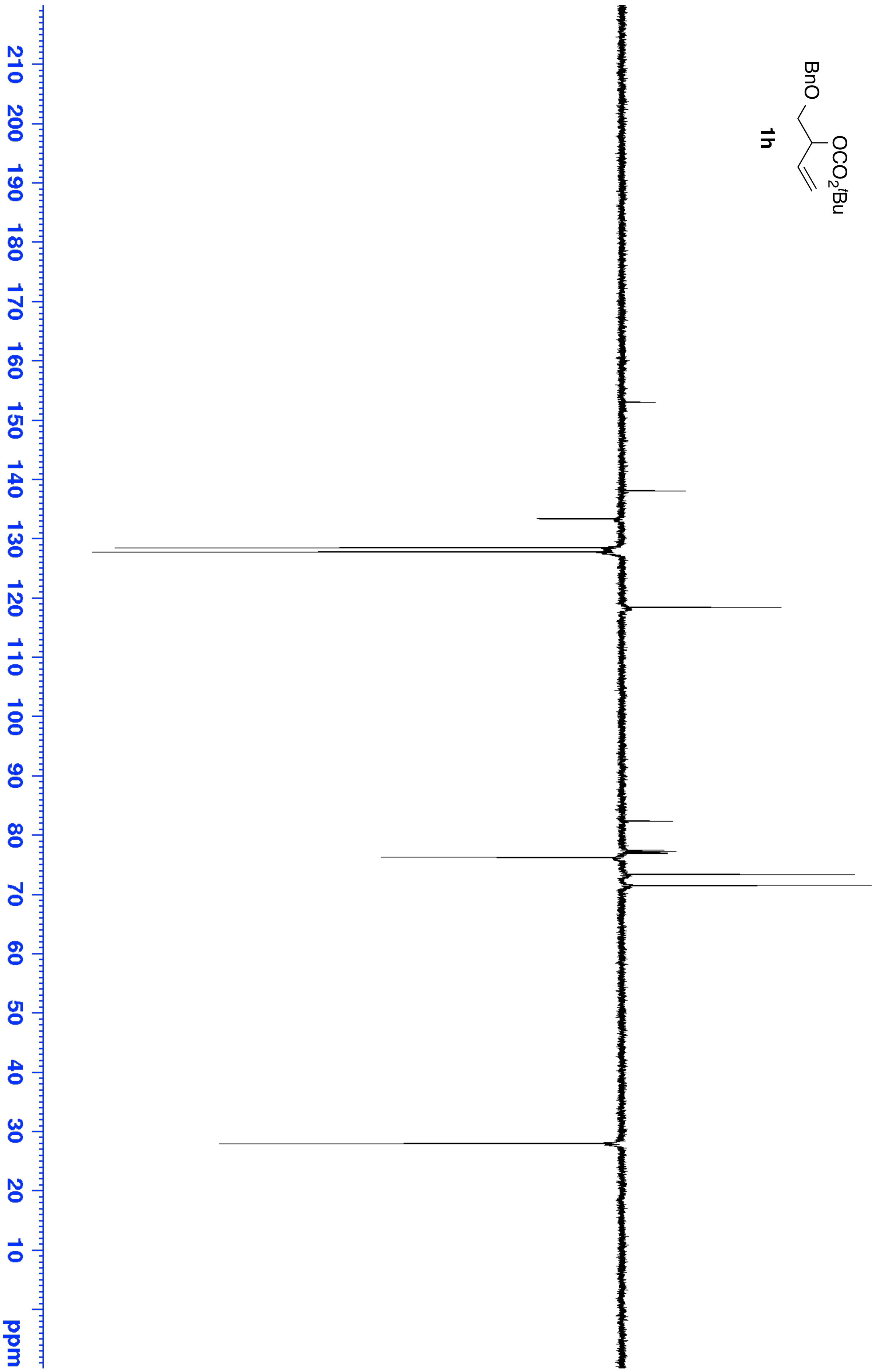
OCO2tBu
BnO
1h
210 200 190 180 170 160 150 140 130 120 110 100 90 80 70 60 50 40 30 20 10 ppm

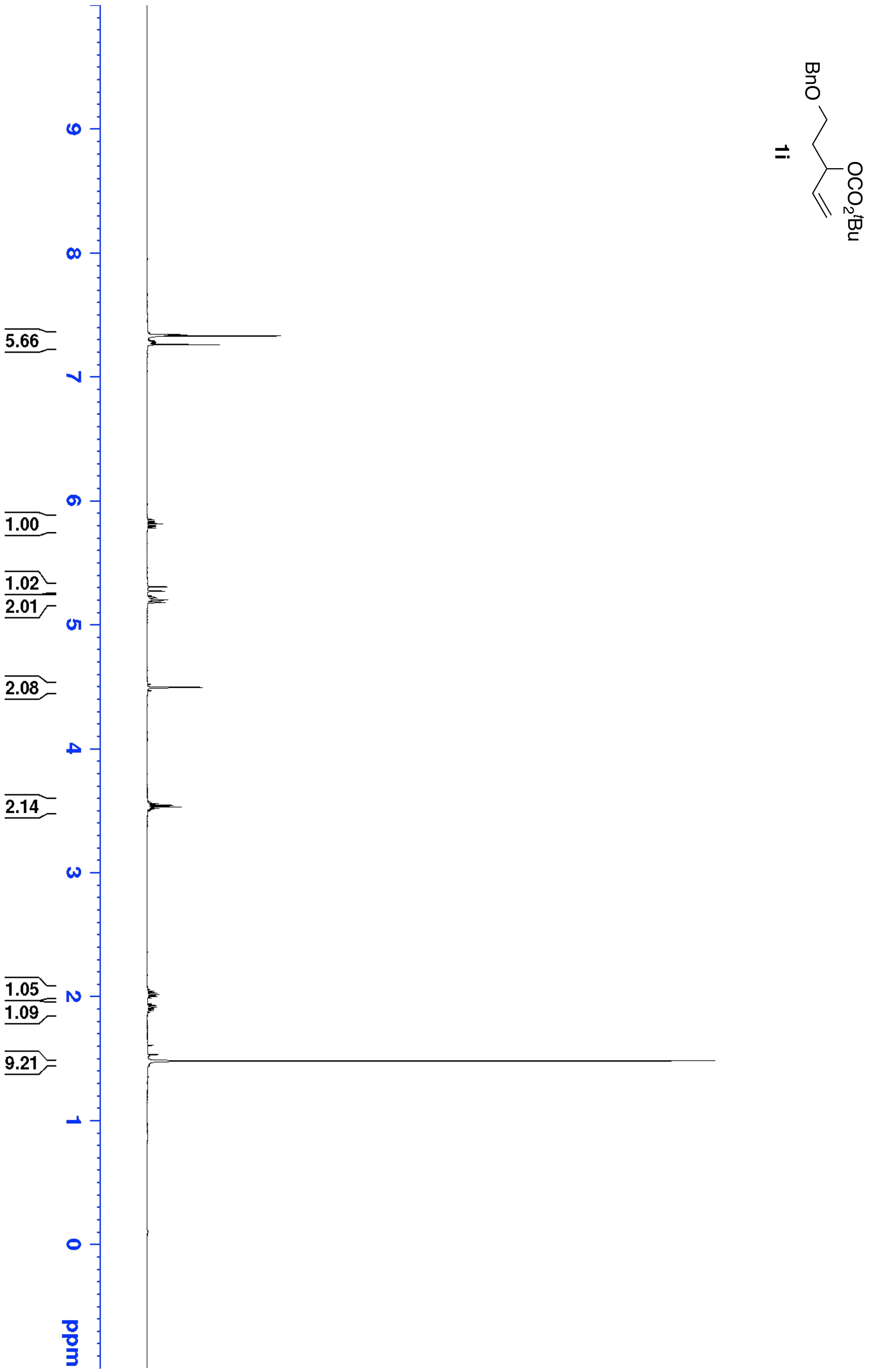
BnO
OCO2tBu
1i
5.66
1.00
1.02
2.01
2.08
2.14
1.05
1.09
9.21
9
8
7
6
5
4
3
2
1
0
ppm

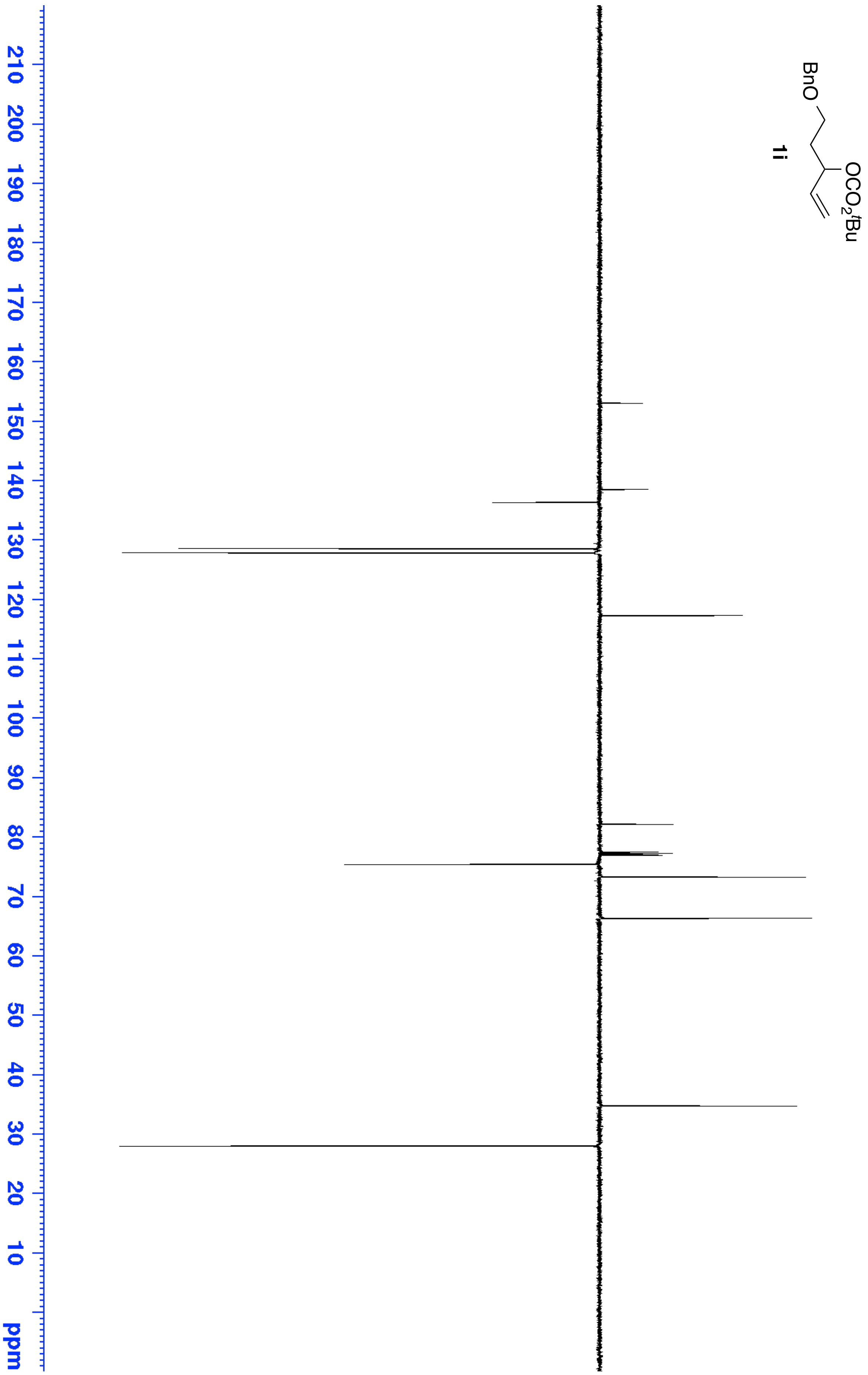

BnO
OCO2tBu
1i
210 200 190 180 170 160 150 140 130 120 110 100 90 80 70 60 50 40 30 20 10 ppm

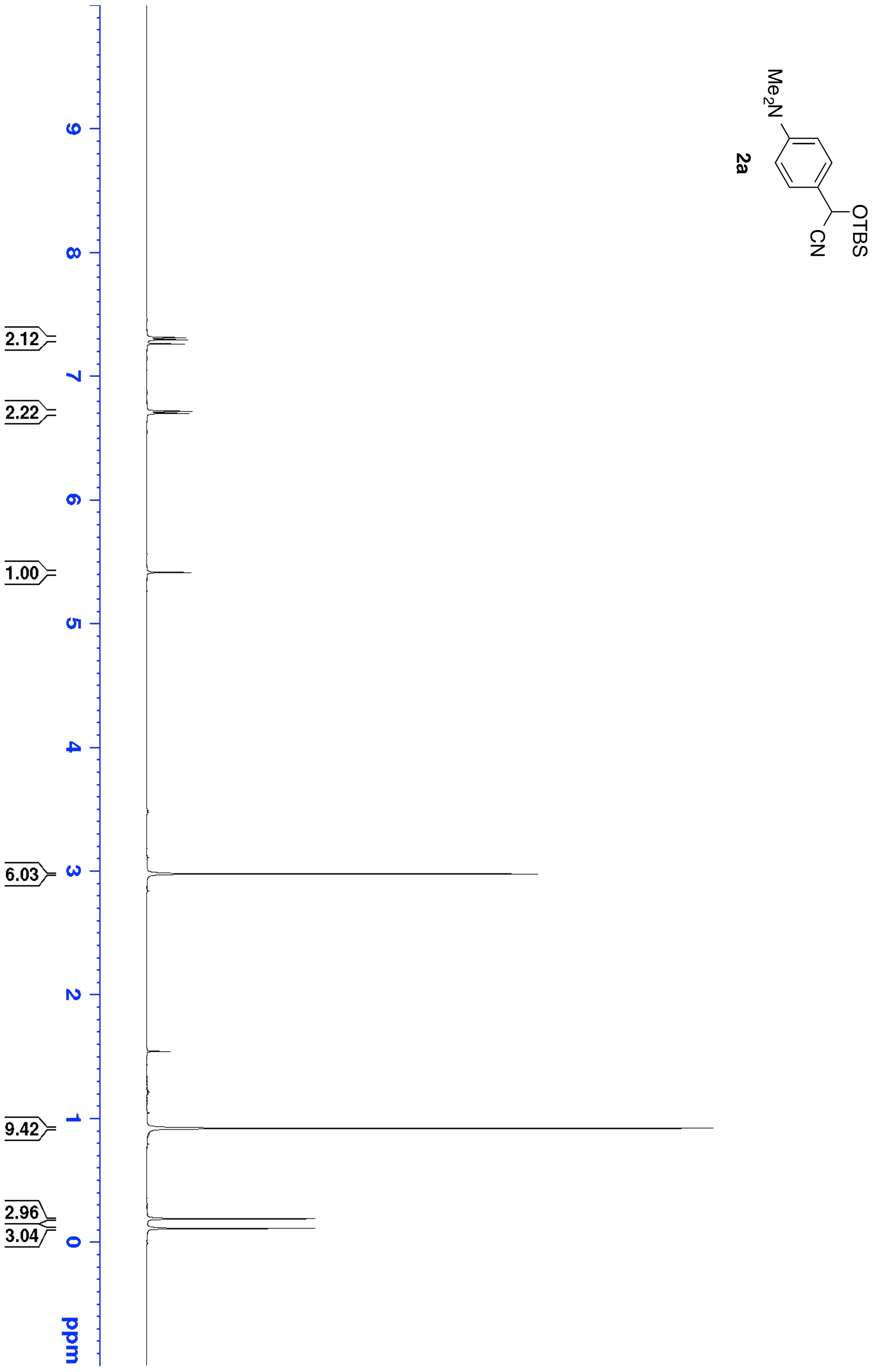
Me2N
OTBS
CN
2a
2.12
2.22
1.00
6.03
9.42
2.96
3.04
9
8
7
6
5
4
3
2
1
0
ppm

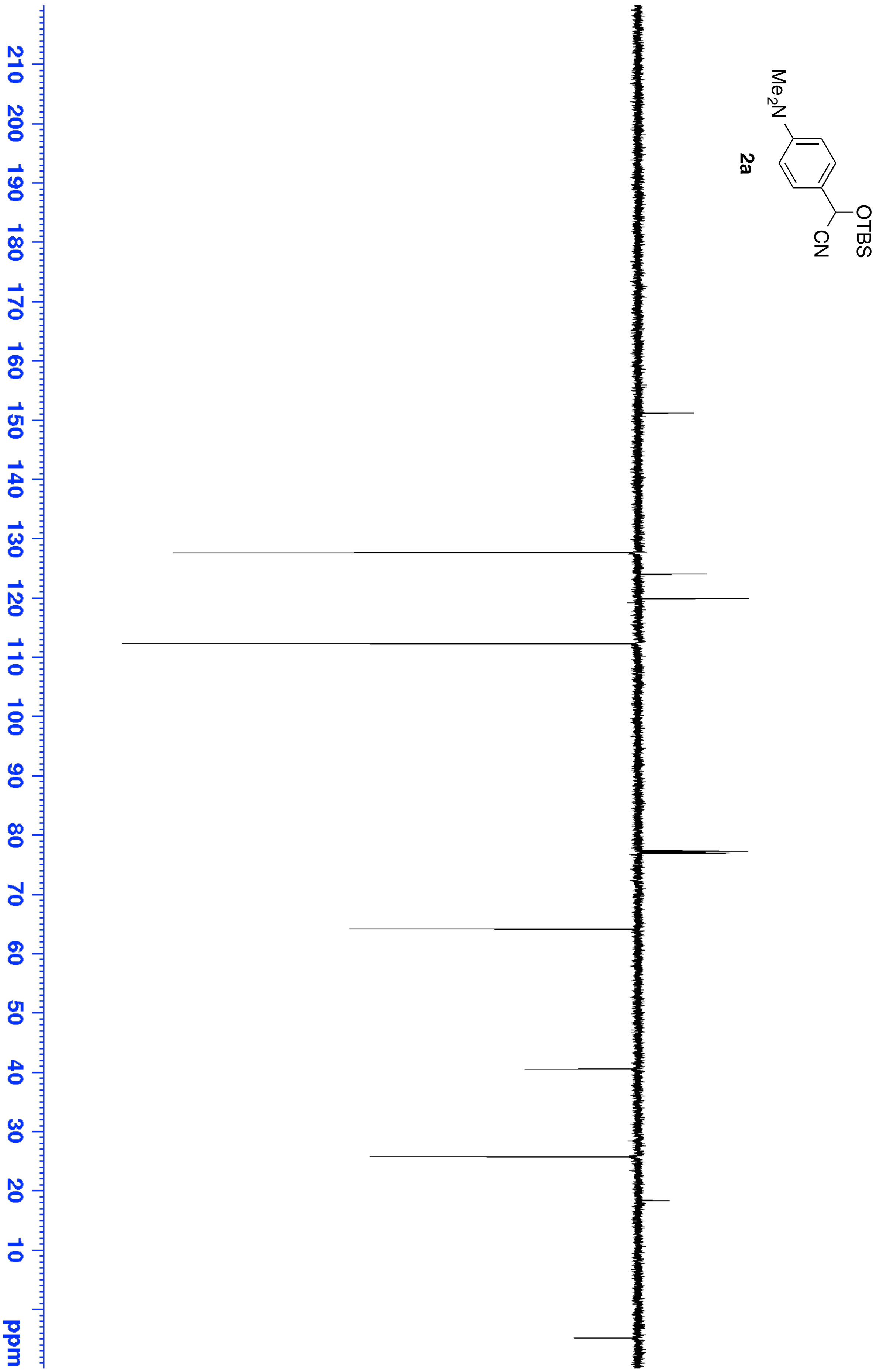

Me2N
OTBS
CN
2a
210 200 190 180 170 160 150 140 130 120 110 100 90 80 70 60 50 40 30 20 10 ppm

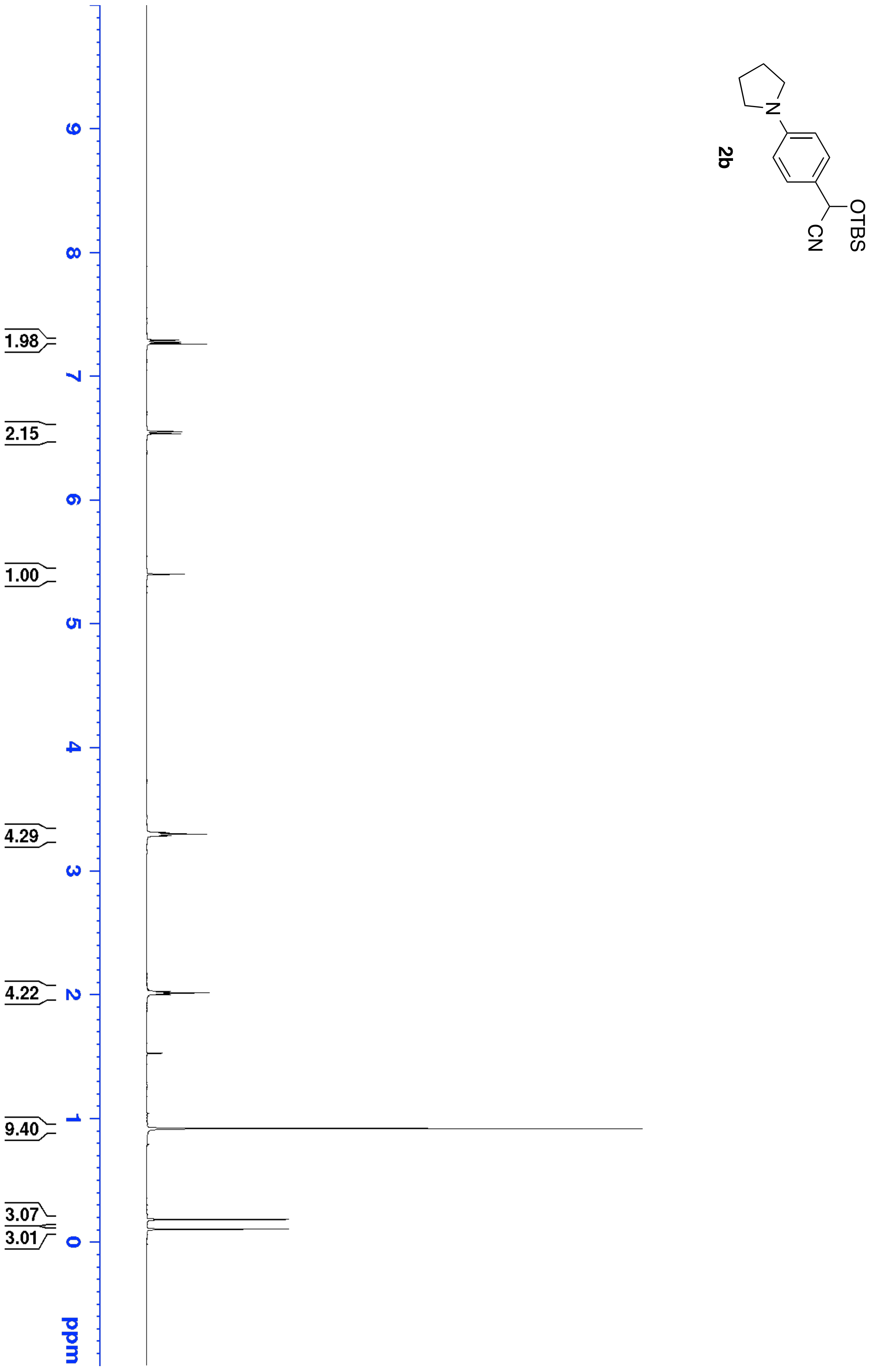
2b
OTBS
CN
N
1.98
2.15
1.00
4.29
4.22
9.40
3.07
3.01
9
8
7
6
5
4
3
2
1
0
ppm

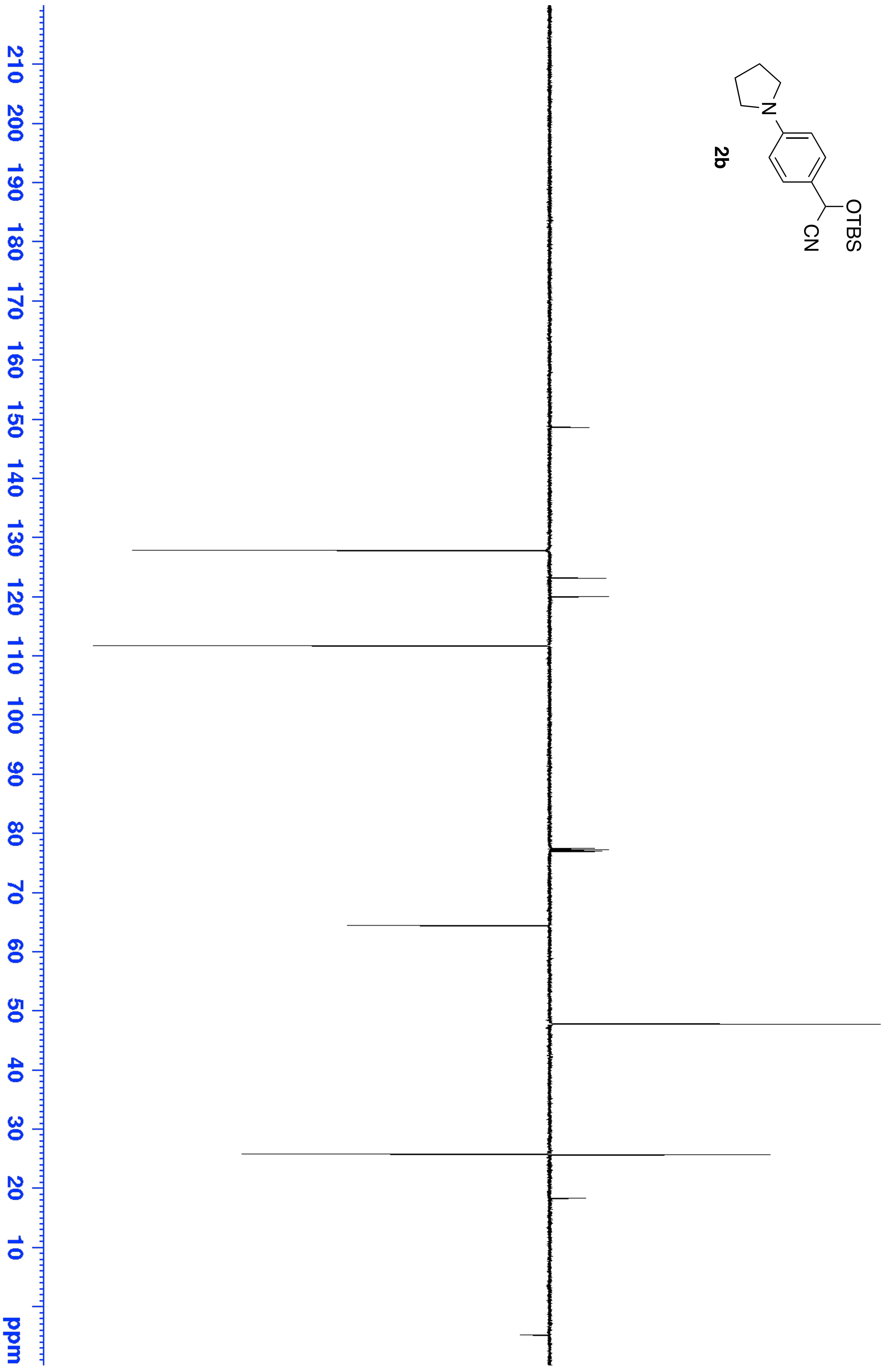
OTBS
CN
N
2b
210 200 190 180 170 160 150 140 130 120 110 100 90 80 70 60 50 40 30 20 10 ppm

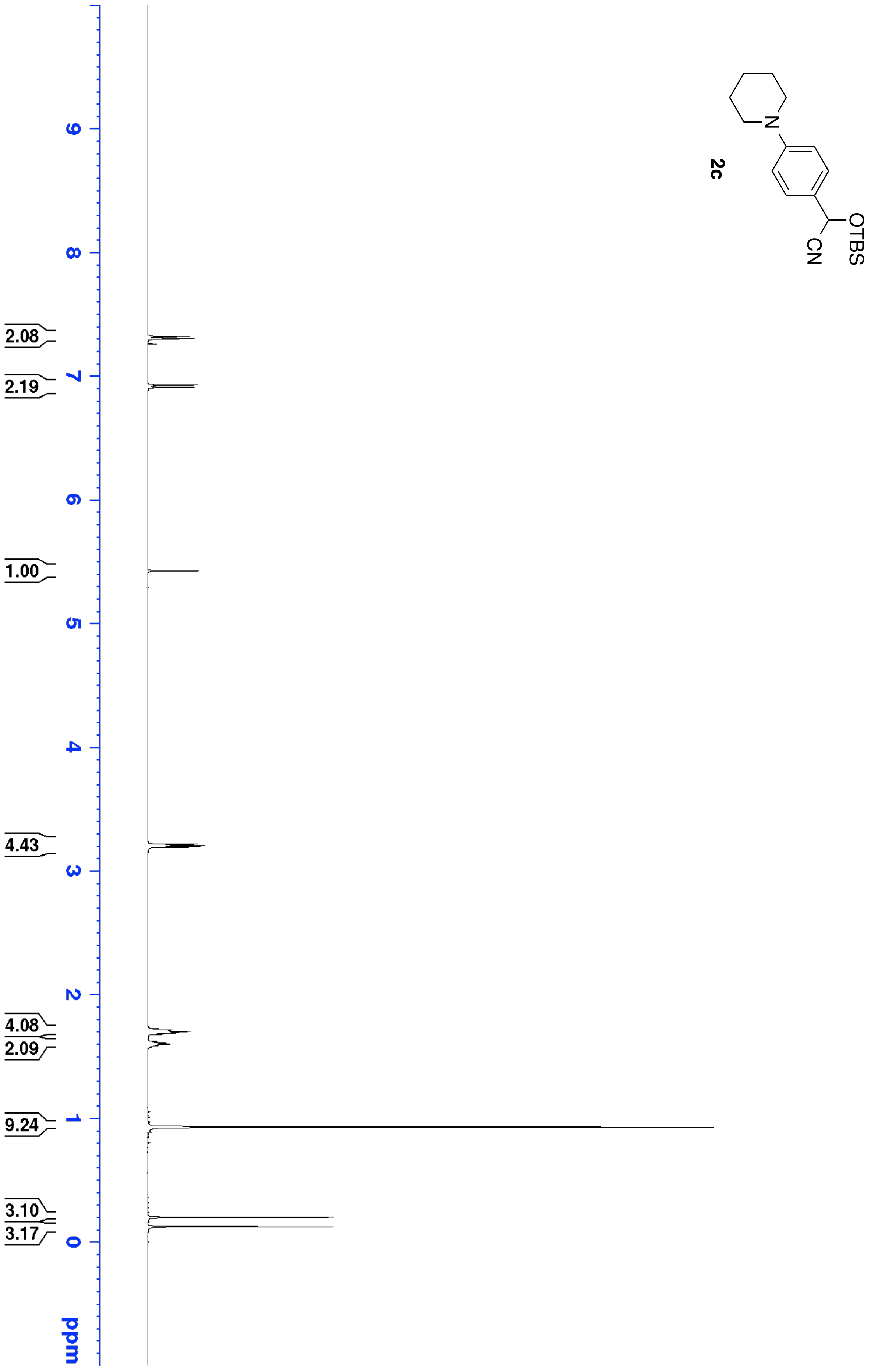

2c
OTBS
CN
N
ppm
9
8
7
6
5
4
3
2
1
0
2.08
2.19
1.00
4.43
4.08
2.09
9.24
3.10
3.17

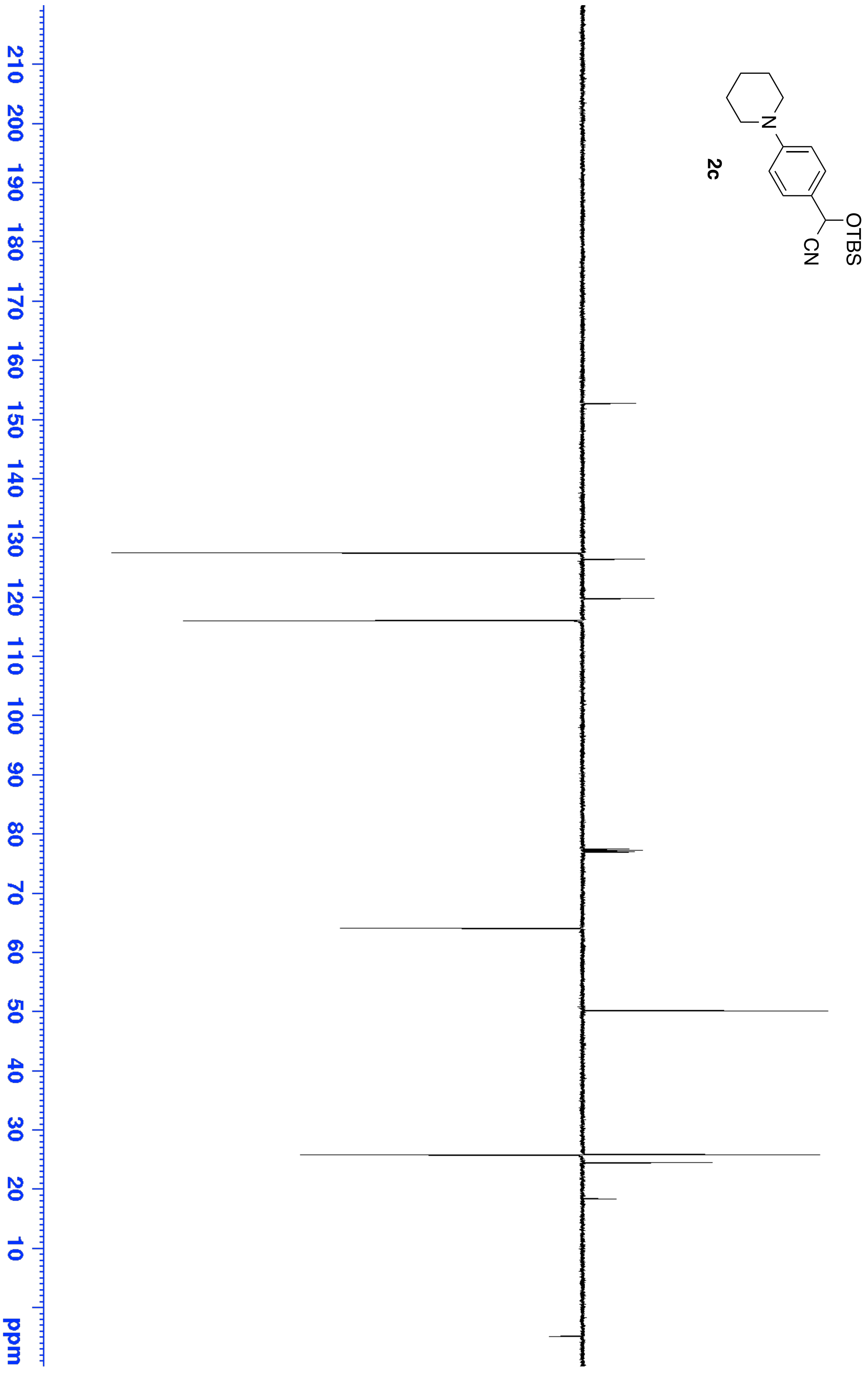
OTBS
CN
N
2c
210 200 190 180 170 160 150 140 130 120 110 100 90 80 70 60 50 40 30 20 10 ppm

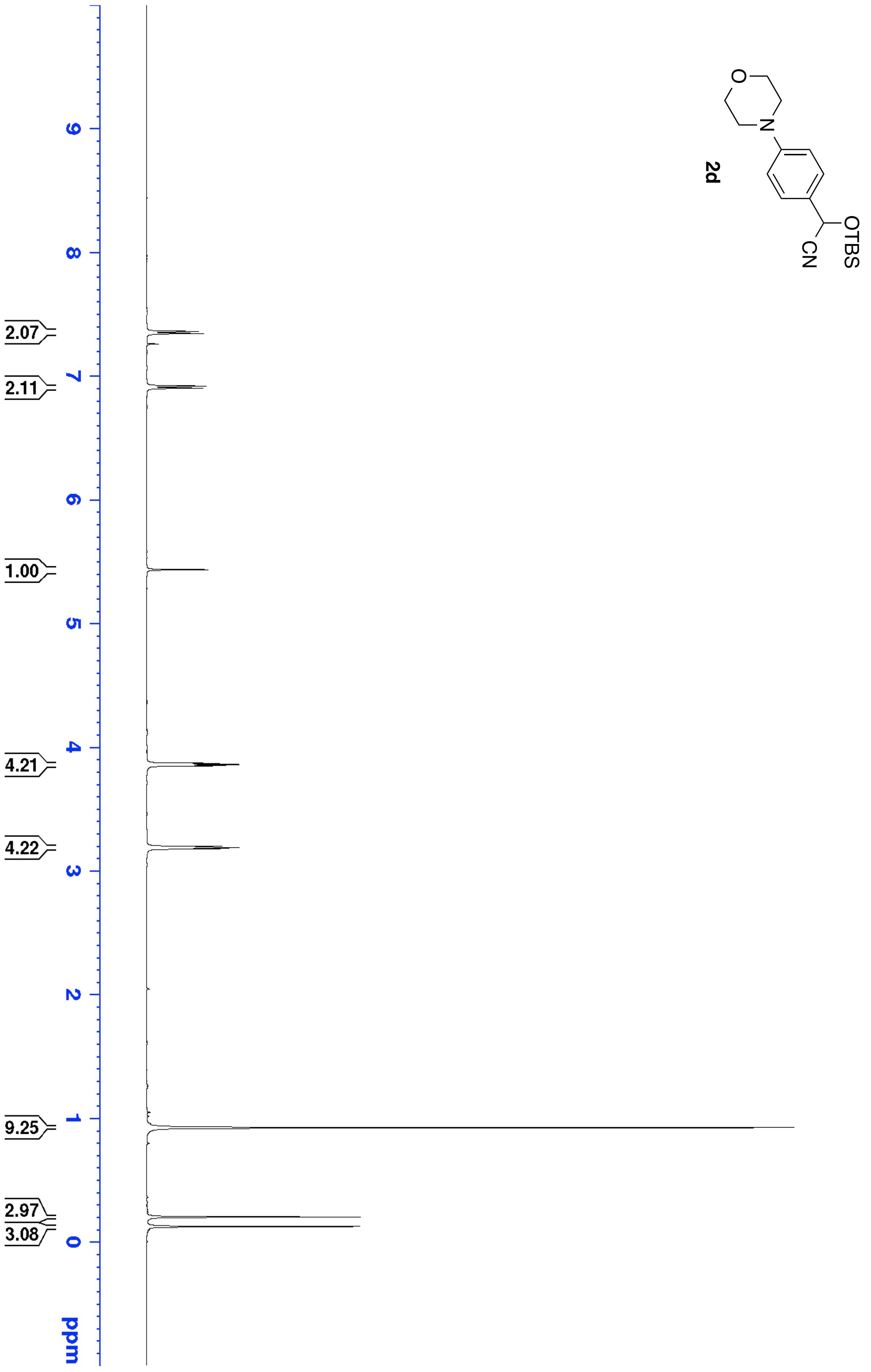
2d
OTBS
CN
N
O
2.07
2.11
1.00
4.21
4.22
9.25
2.97
3.08
9
8
7
6
5
4
3
2
1
0
ppm

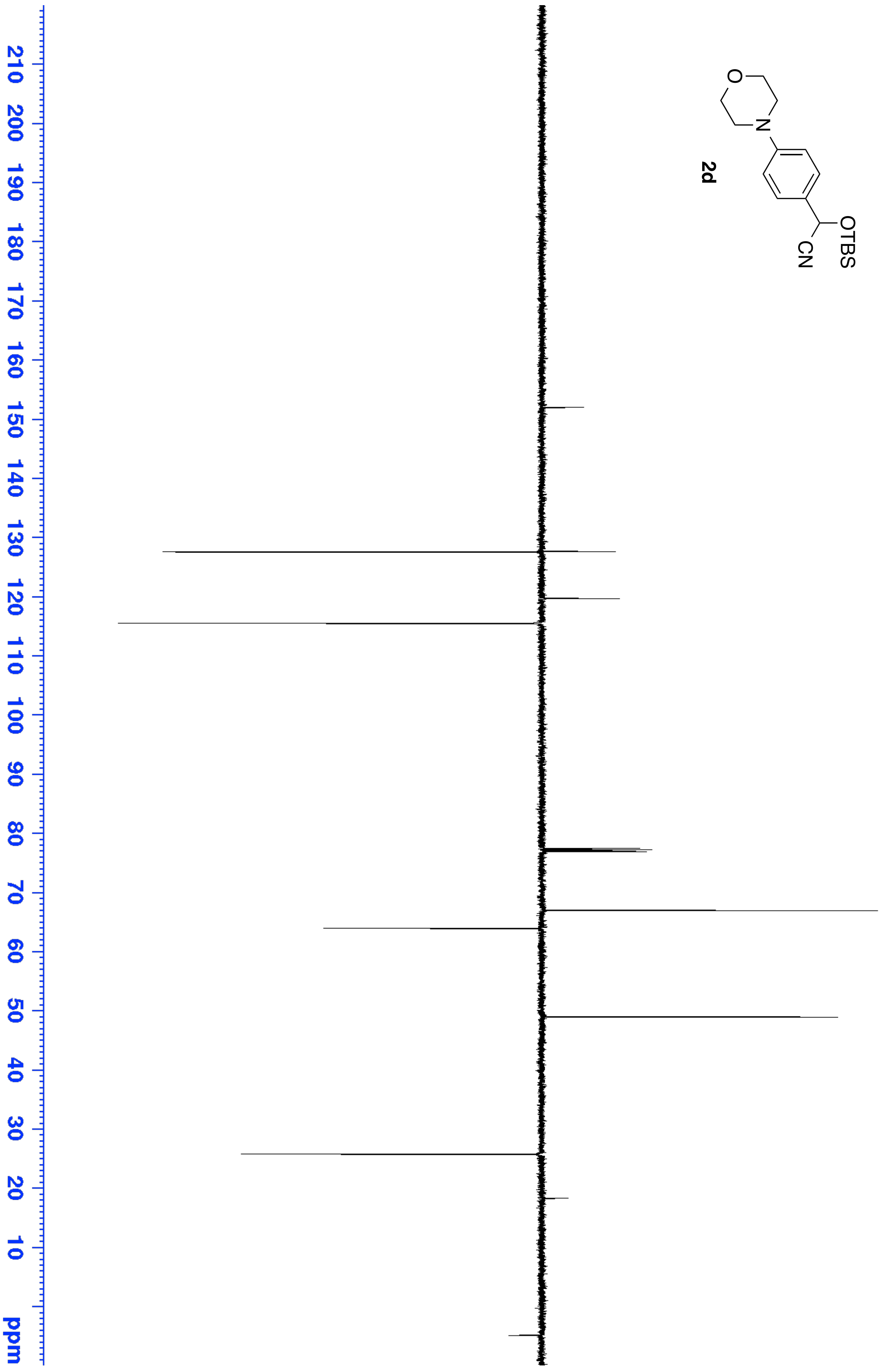

OTBS
CN
O
N
2d
210 200 190 180 170 160 150 140 130 120 110 100 90 80 70 60 50 40 30 20 10 ppm

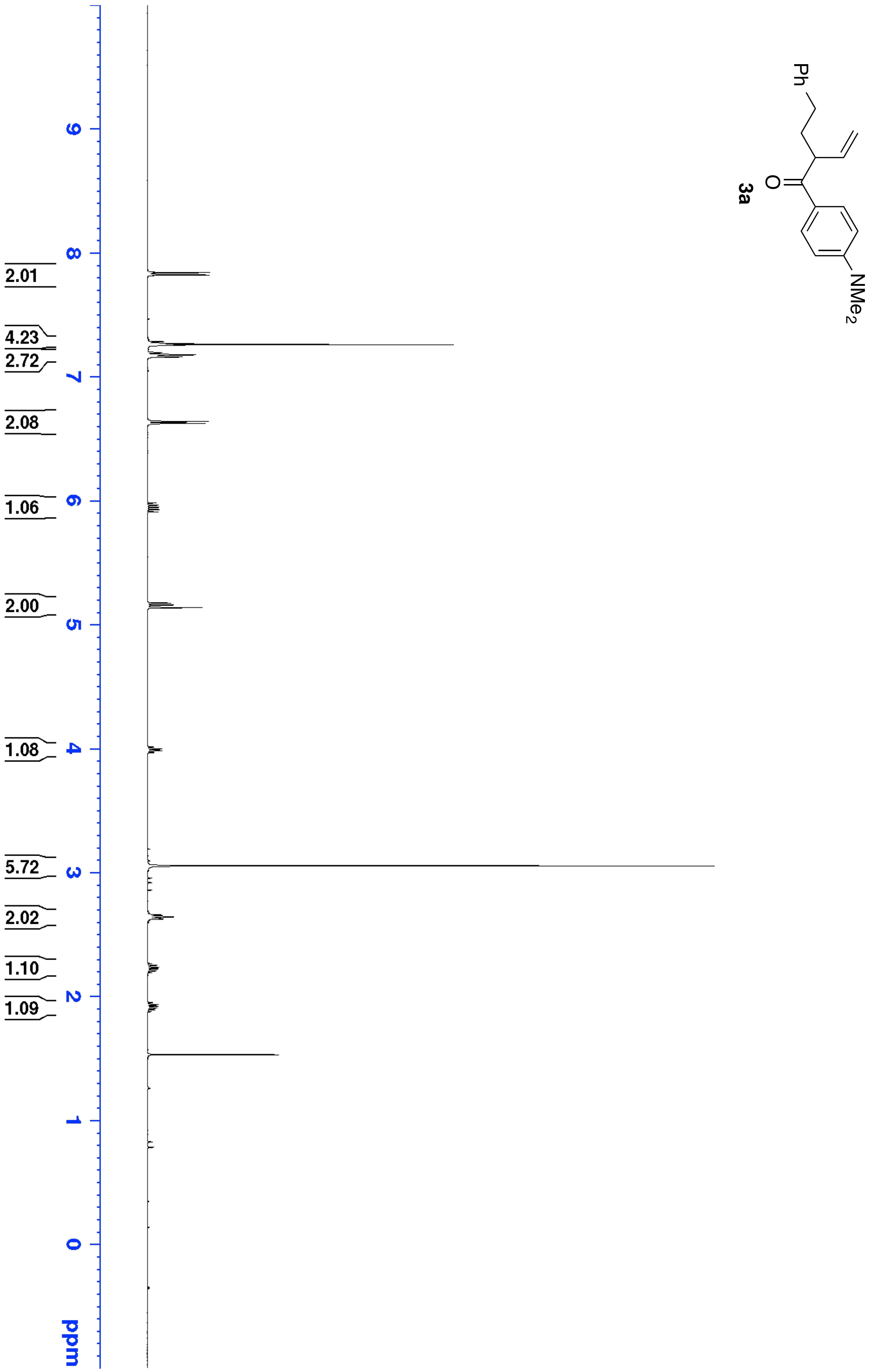
3a
Ph
O
NMe2
2.01
4.23
2.72
2.08
1.06
2.00
1.08
5.72
2.02
1.10
1.09
9
8
7
6
5
4
3
2
1
0
ppm

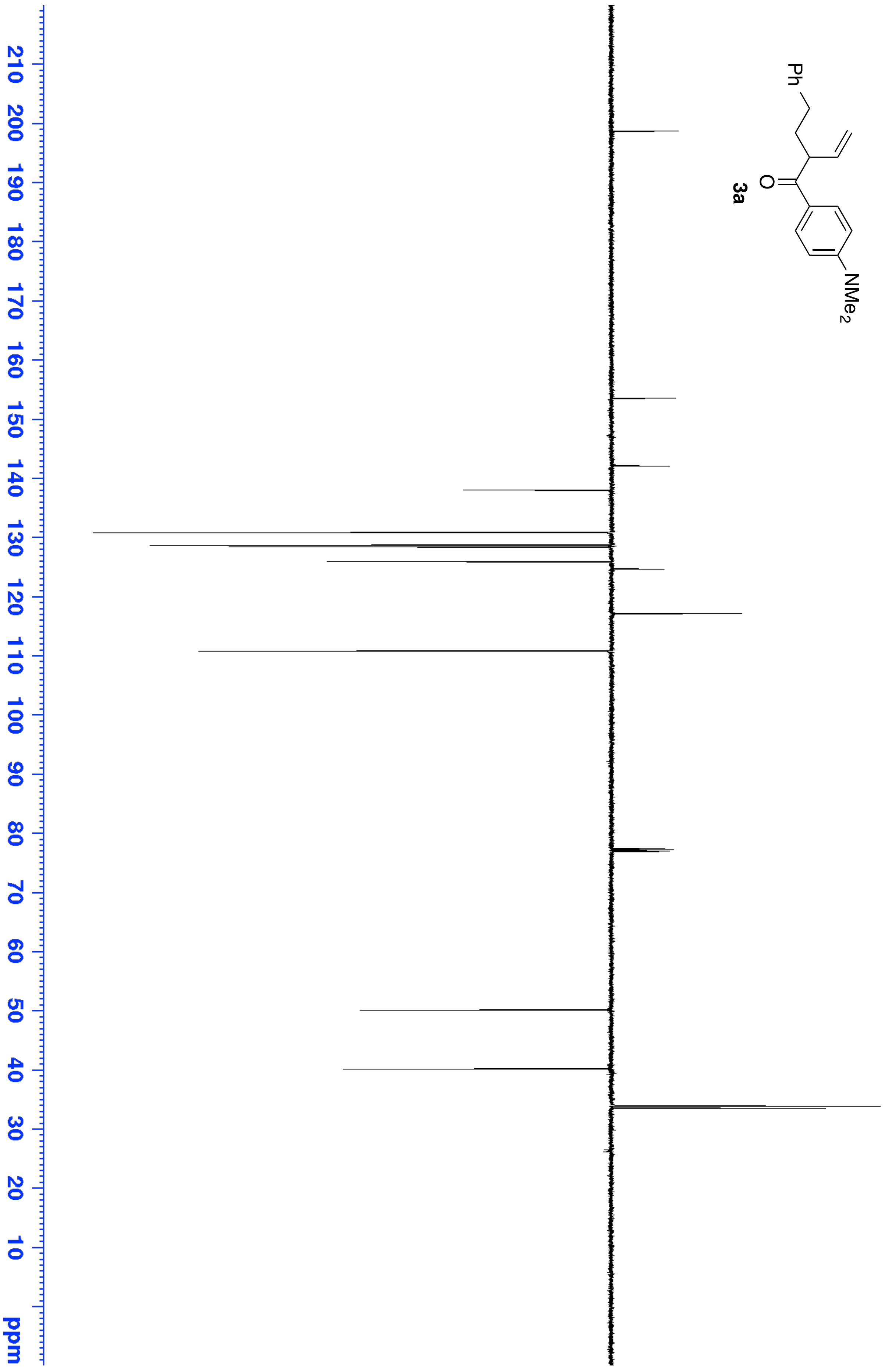
Ph
O
3a
NMe2
210
200
190
180
170
160
150
140
130
120
110
100
90
80
70
60
50
40
30
20
10
ppm

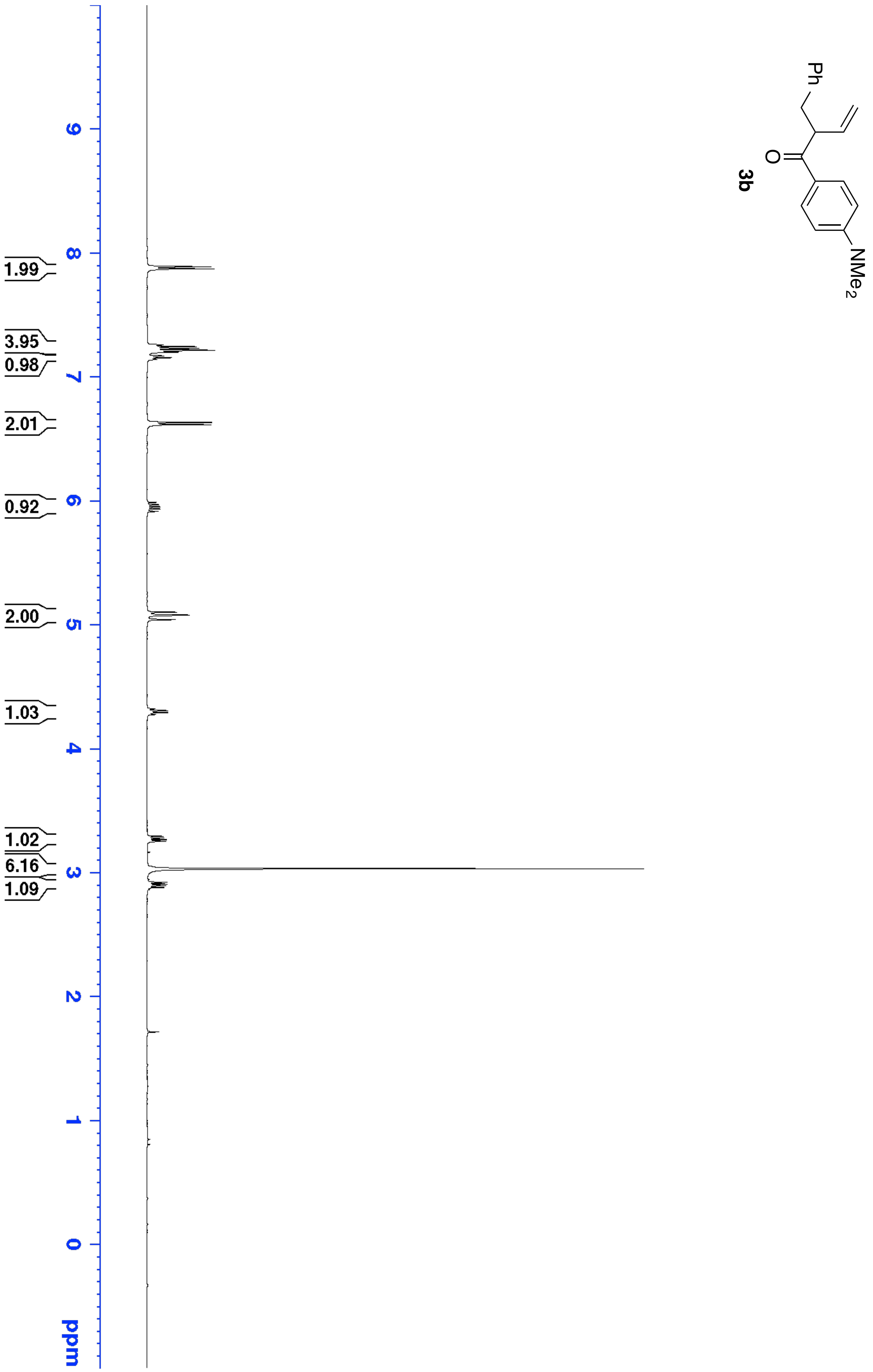

Ph
O
NMe2
3b
1.99
3.95
0.98
2.01
0.92
2.00
1.03
1.02
6.16
1.09
9
8
7
6
5
4
3
2
1
0
ppm

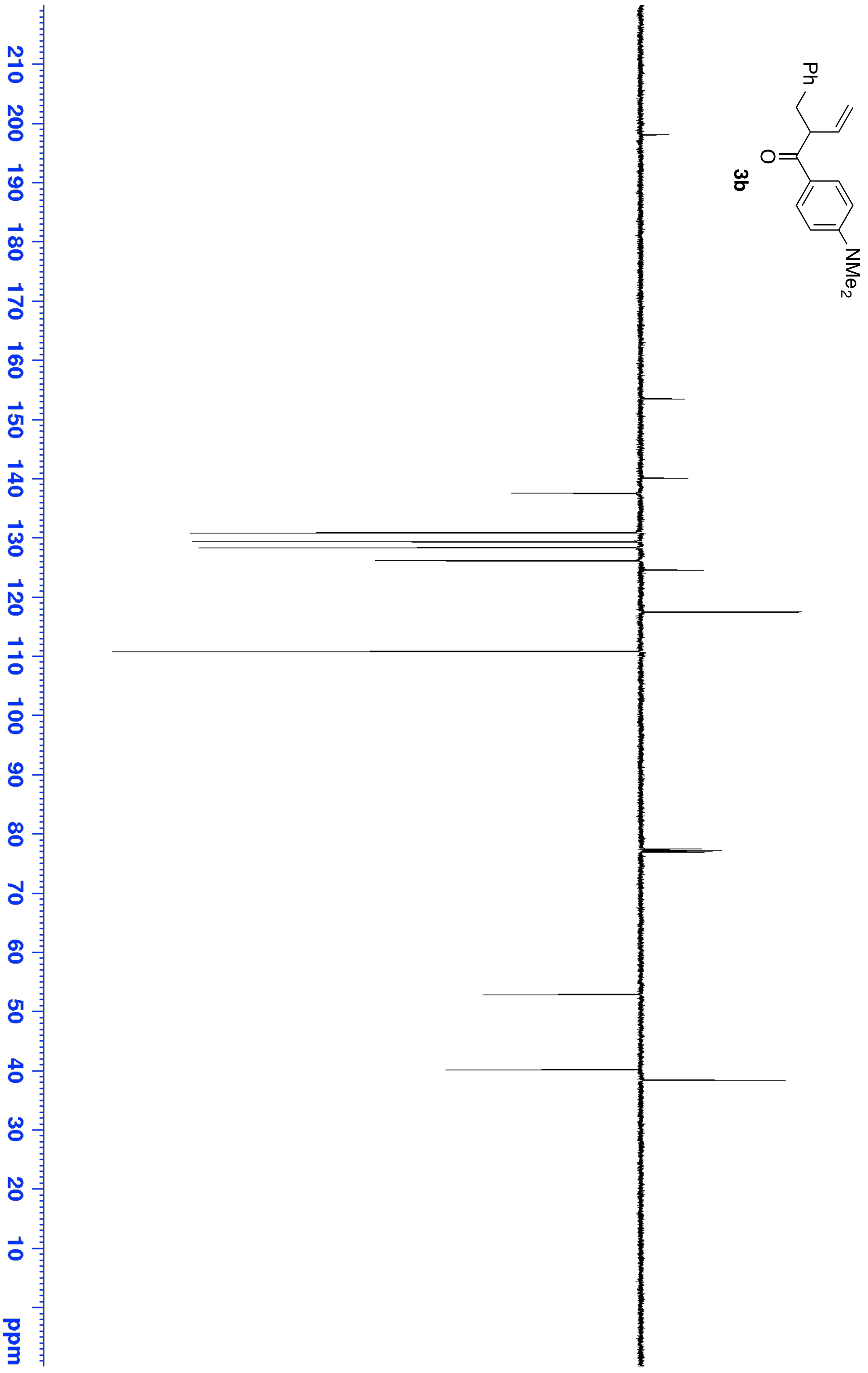
Ph
O
3b
NMe2
210 200 190 180 170 160 150 140 130 120 110 100 90 80 70 60 50 40 30 20 10 ppm

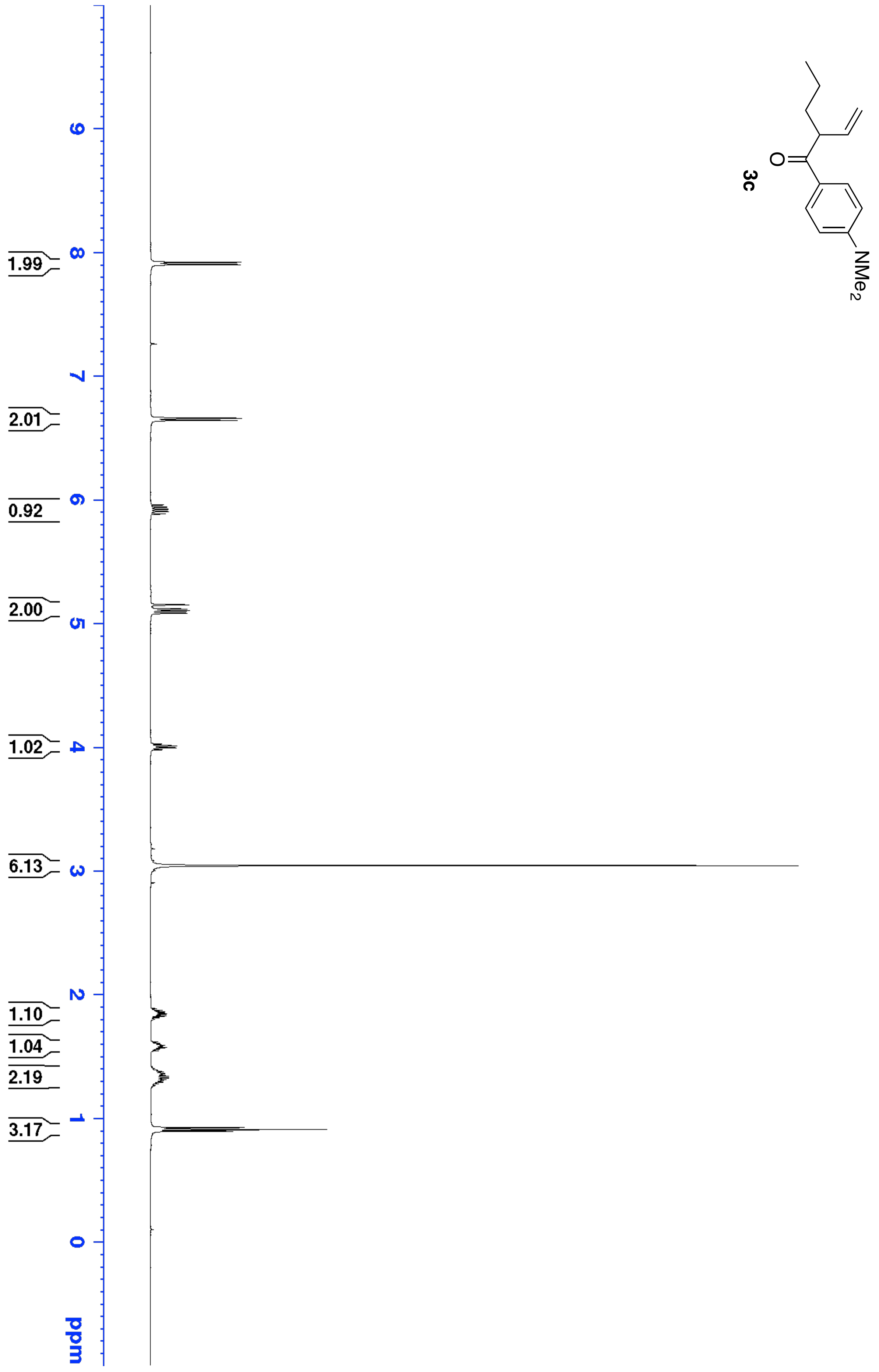
3c
NMe$_2$
O
1.99
2.01
0.92
2.00
1.02
6.13
1.10
1.04
2.19
3.17
9
8
7
6
5
4
3
2
1
0
ppm

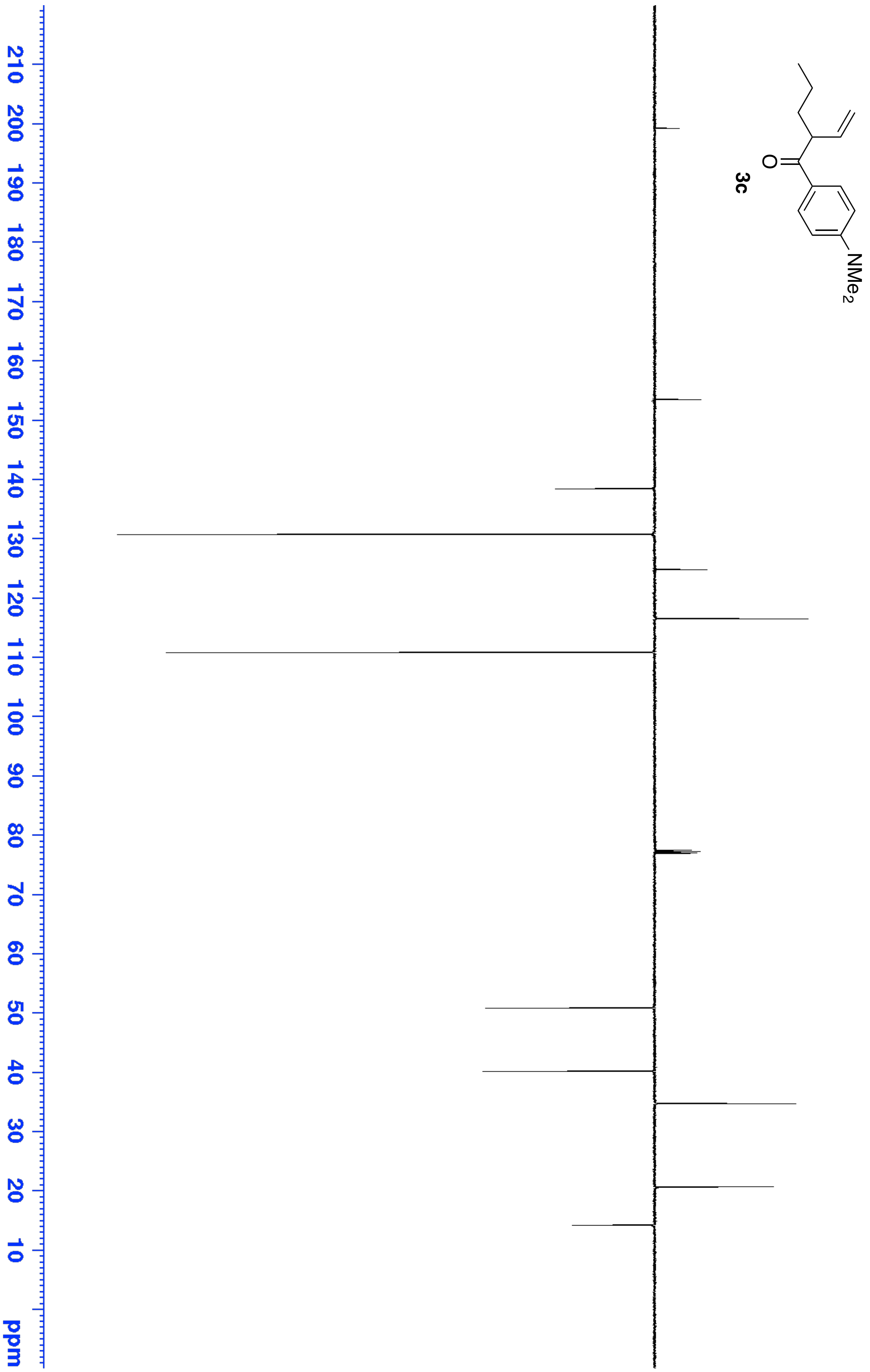
NMe2
O
3c
210 200 190 180 170 160 150 140 130 120 110 100 90 80 70 60 50 40 30 20 10 ppm

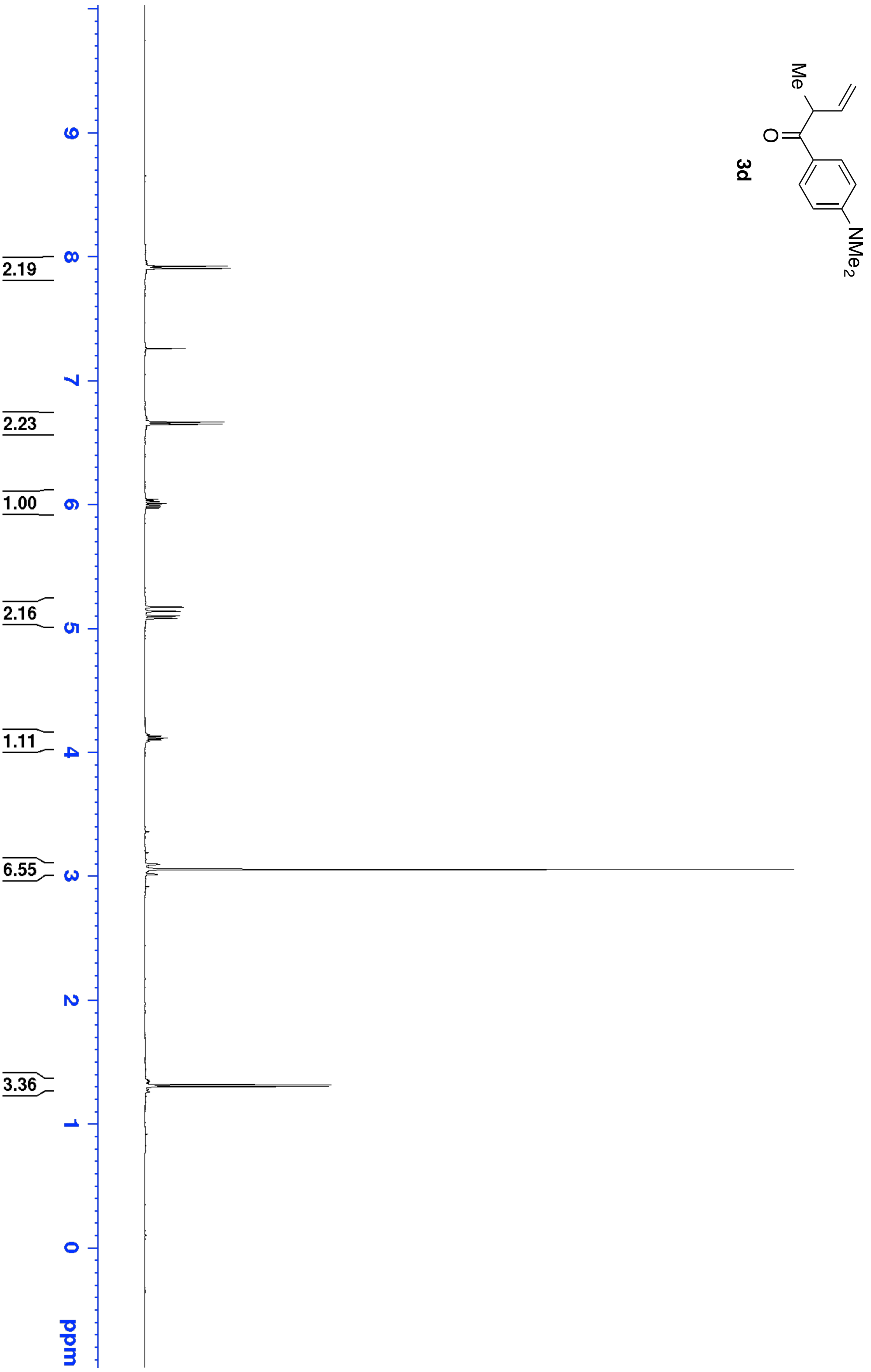

Me
O
NMe2
3d
2.19
2.23
1.00
2.16
1.11
6.55
3.36
9
8
7
6
5
4
3
2
1
0
ppm

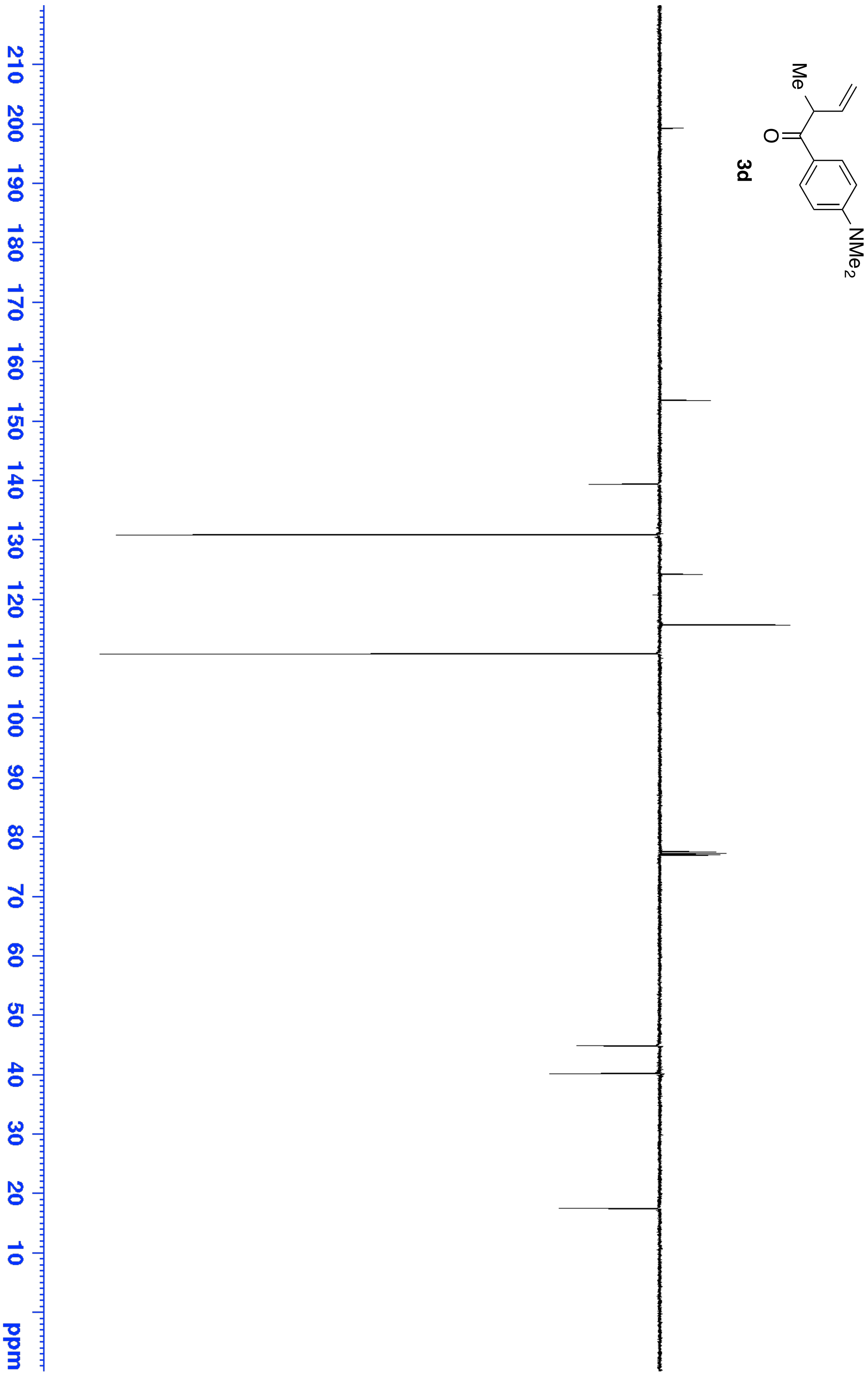
NMe2
Me
O
3d
210
200
190
180
170
160
150
140
130
120
110
100
90
80
70
60
50
40
30
20
10
ppm

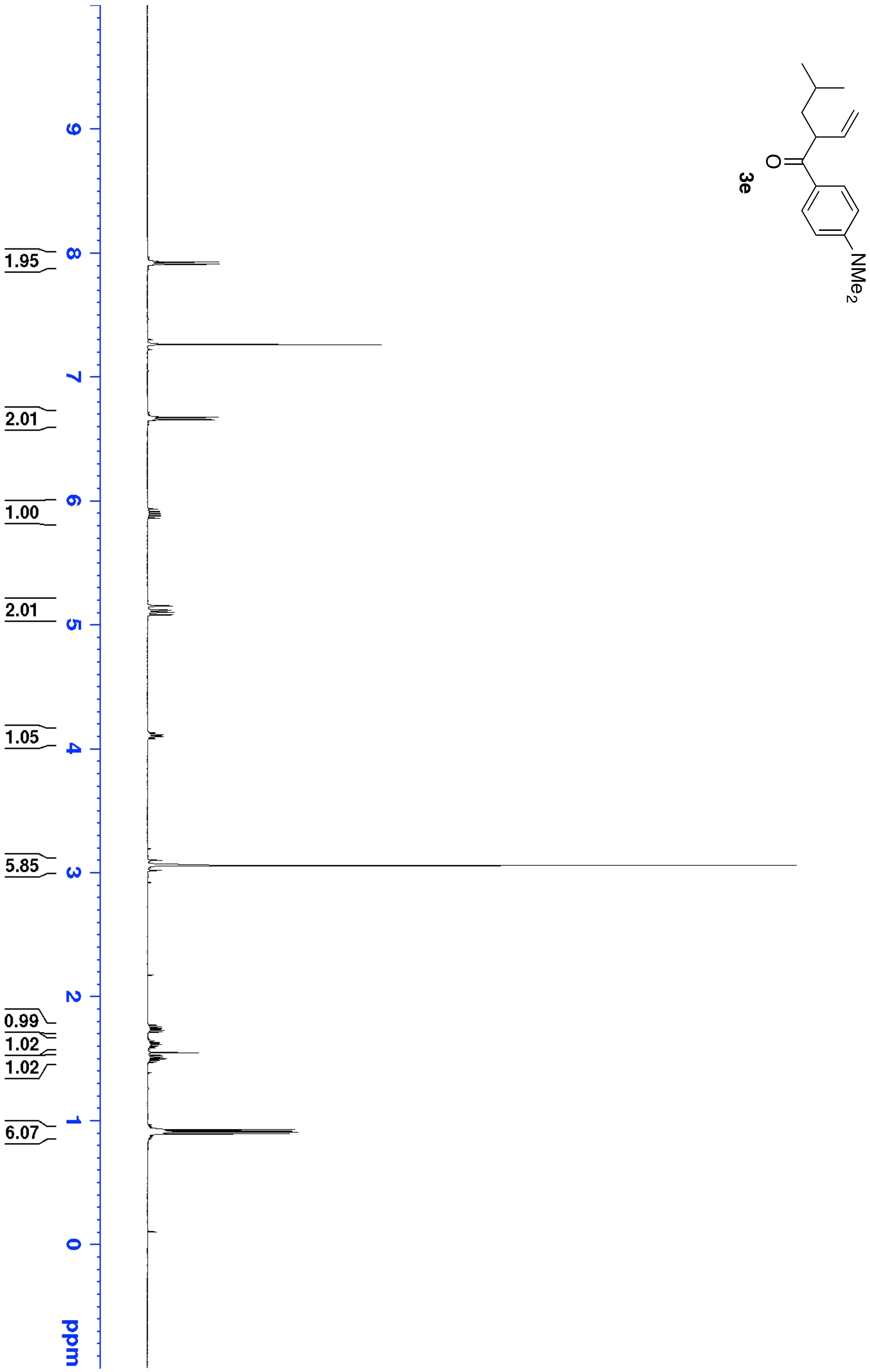
3e
NMe2
O
1.95
2.01
1.00
2.01
1.05
5.85
0.99
1.02
1.02
6.07
9
8
7
6
5
4
3
2
1
0
ppm

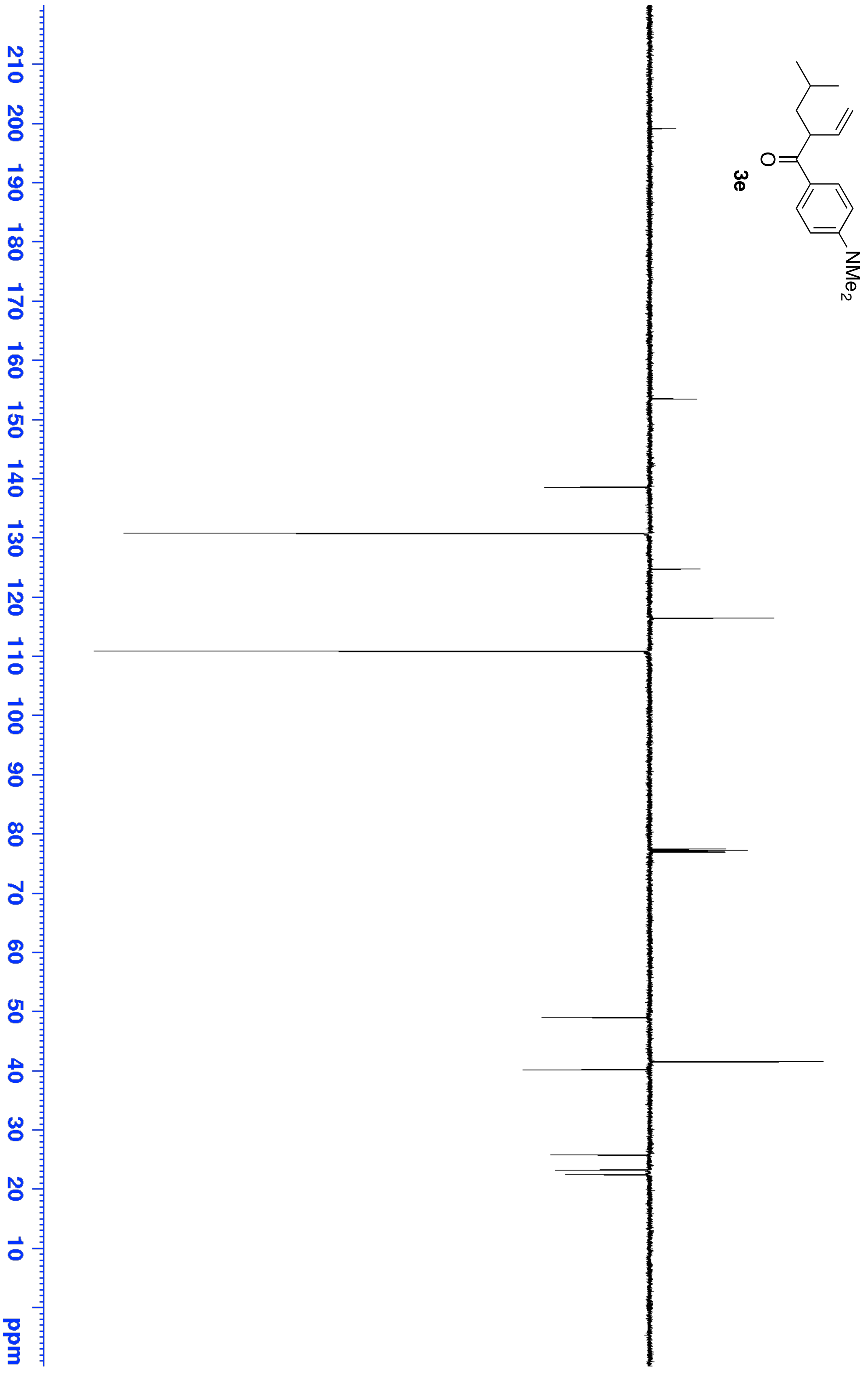
NMe2
O
3e
210 200 190 180 170 160 150 140 130 120 110 100 90 80 70 60 50 40 30 20 10 ppm

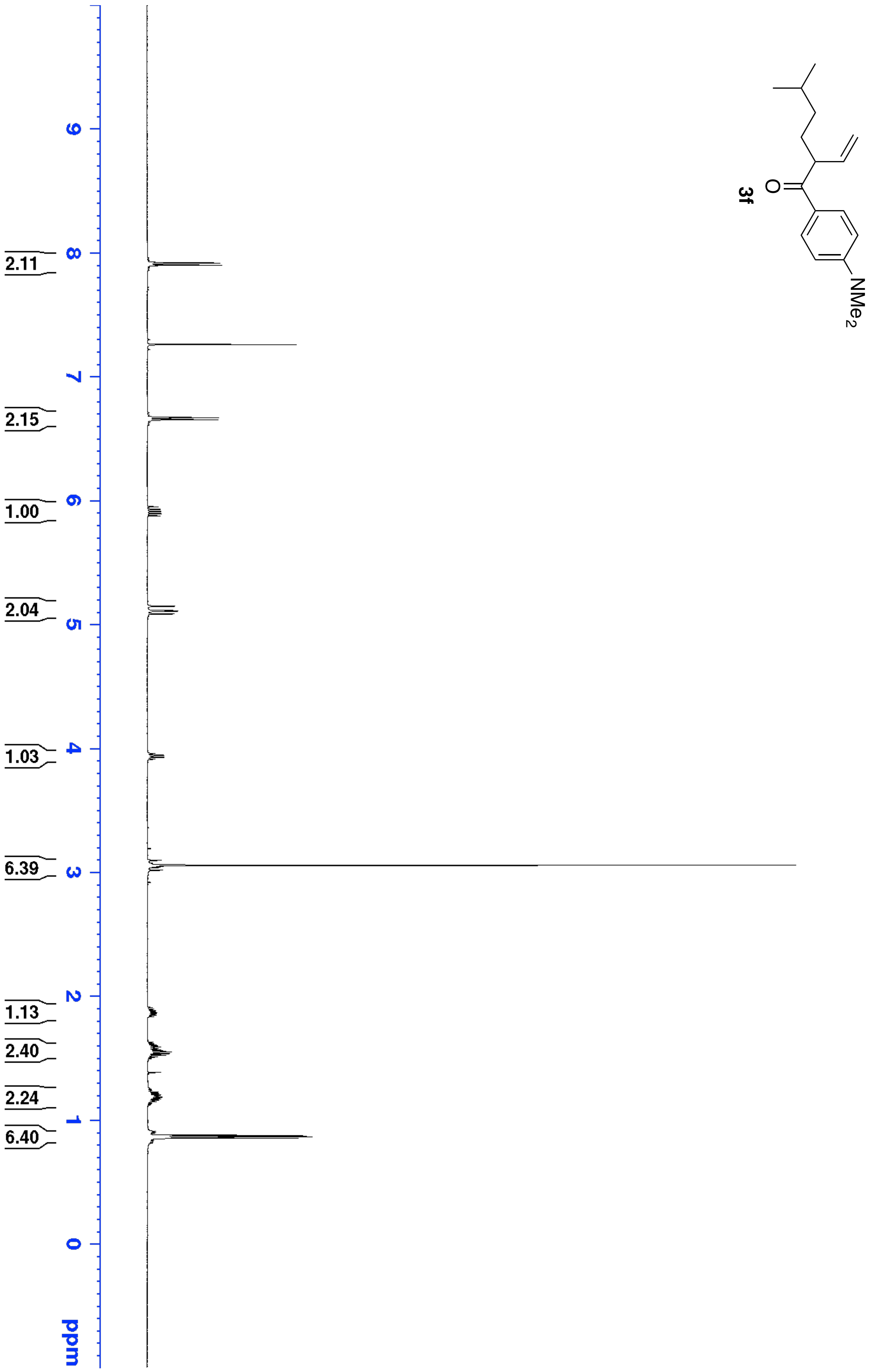
3f
O
NMe2
9
8
7
6
5
4
3
2
1
0
ppm
2.11
2.15
1.00
2.04
1.03
6.39
1.13
2.40
2.24
6.40

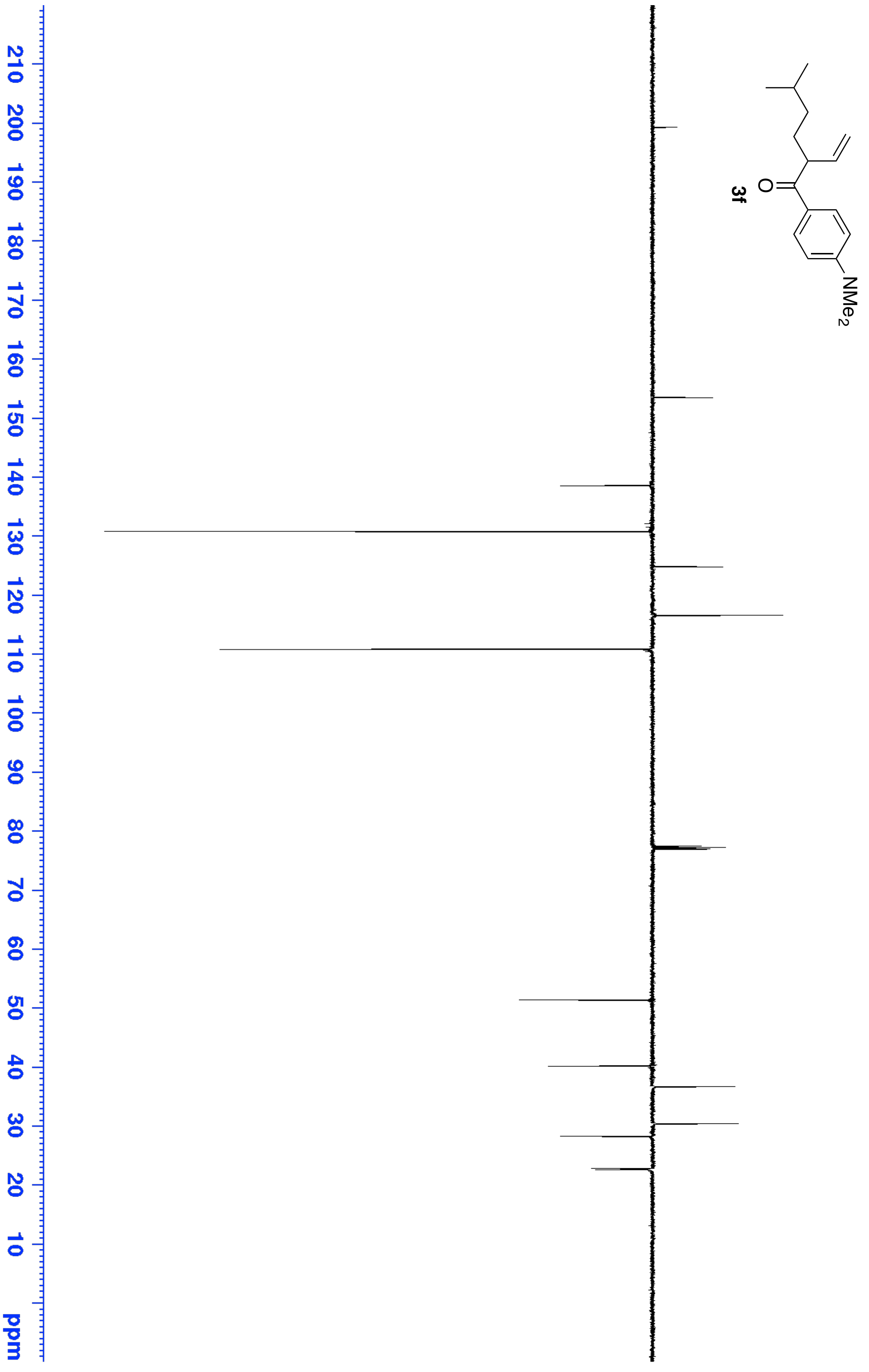
NMe2
O
3f
210 200 190 180 170 160 150 140 130 120 110 100 90 80 70 60 50 40 30 20 10
ppm

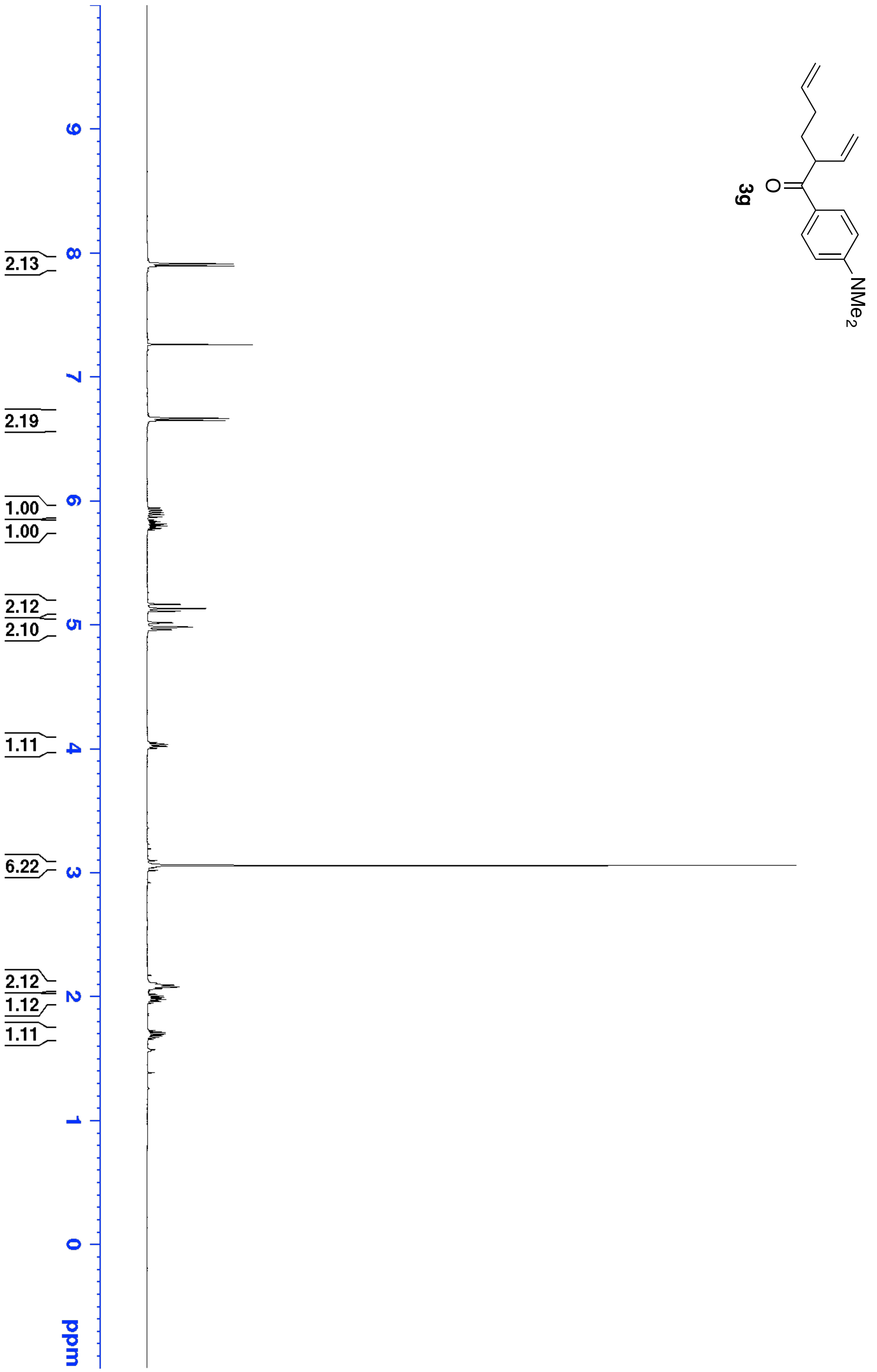
3g
NMe2
2.13
2.19
1.00
1.00
2.12
2.10
1.11
6.22
2.12
1.12
1.11
9
8
7
6
5
4
3
2
1
0
ppm

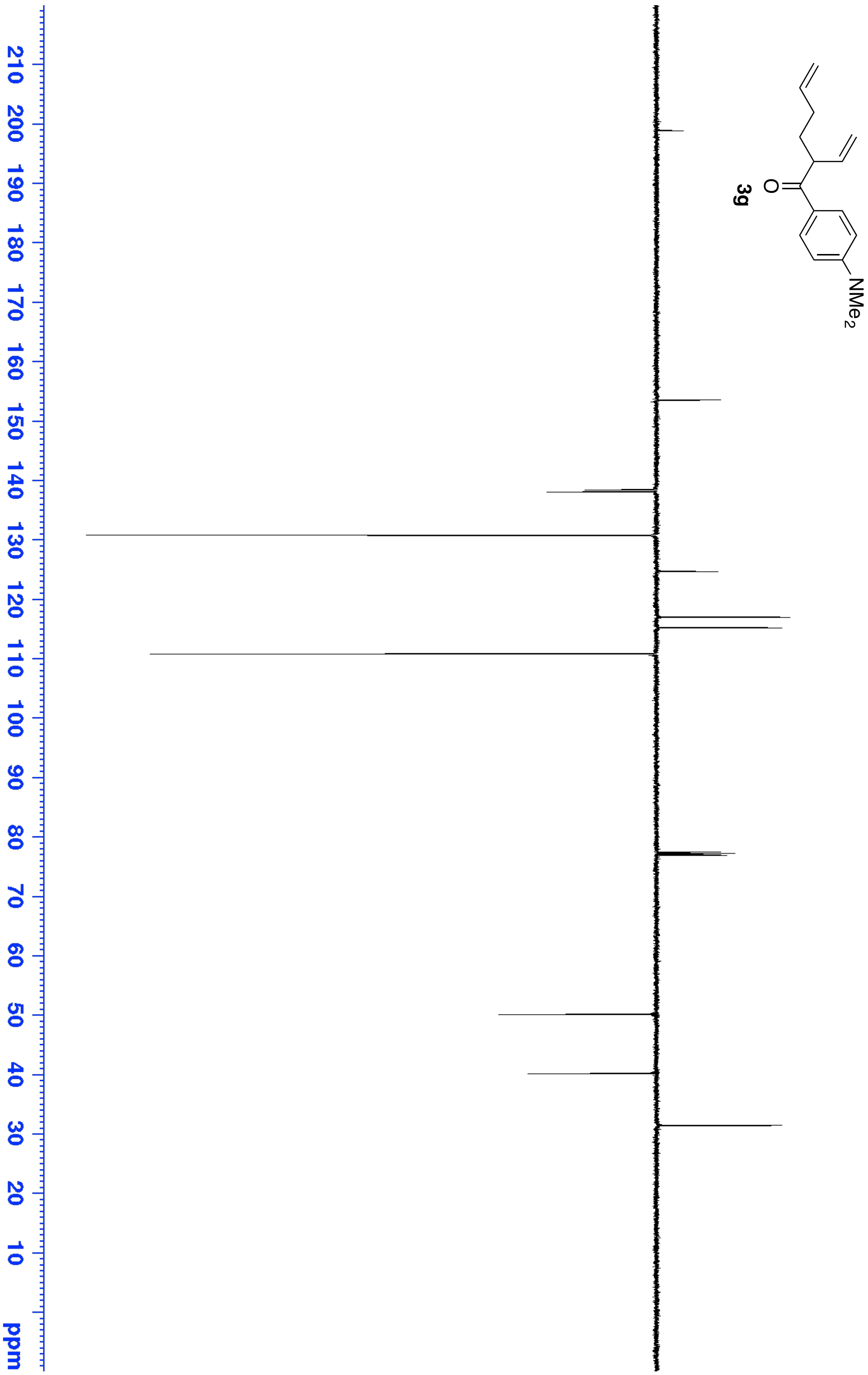
NMe2
O
3g
210 200 190 180 170 160 150 140 130 120 110 100 90 80 70 60 50 40 30 20 10 ppm

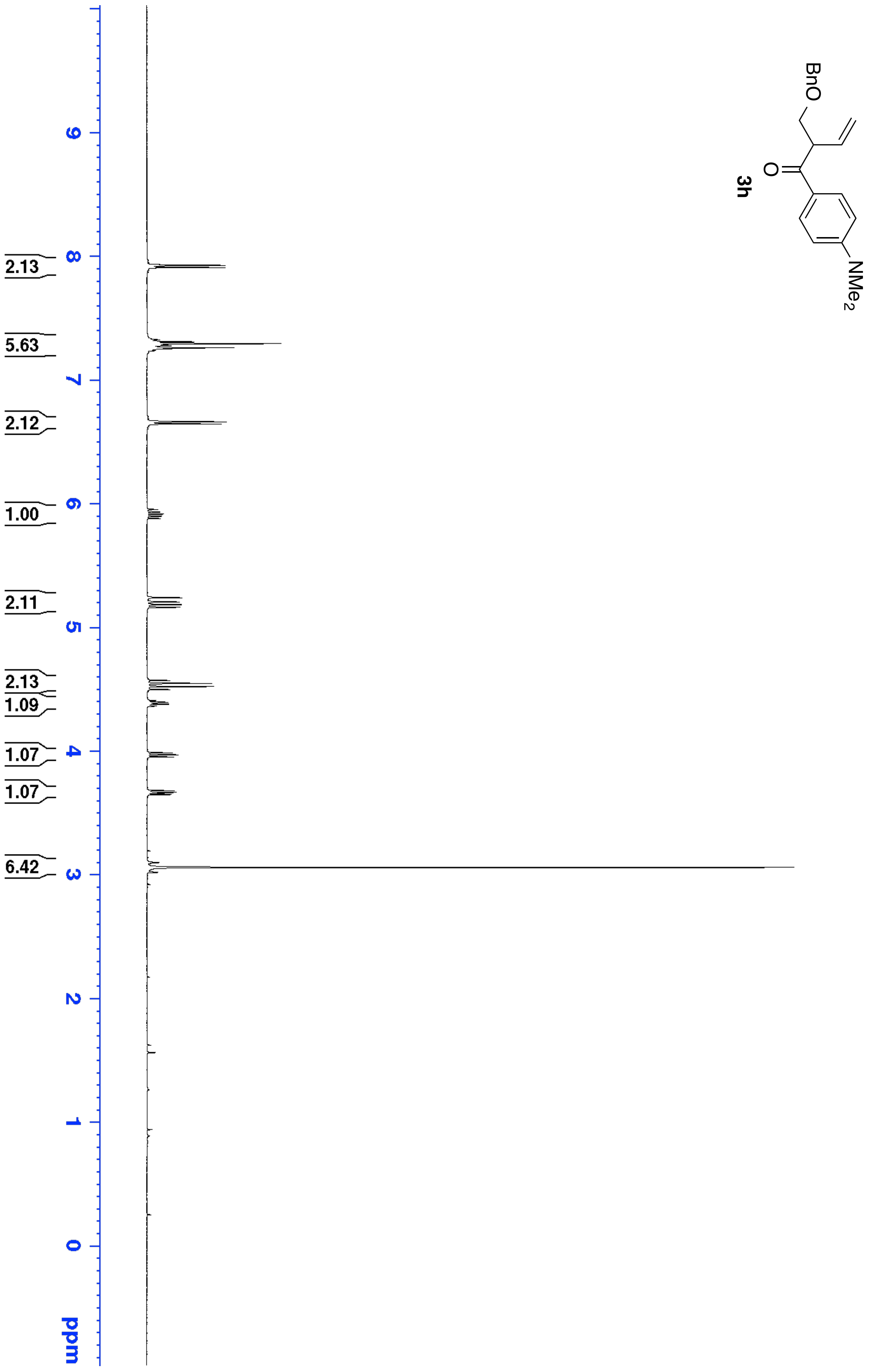
BnO
O
NMe2
3h
2.13
5.63
2.12
1.00
2.11
2.13
1.09
1.07
1.07
6.42
9
8
7
6
5
4
3
2
1
0
ppm

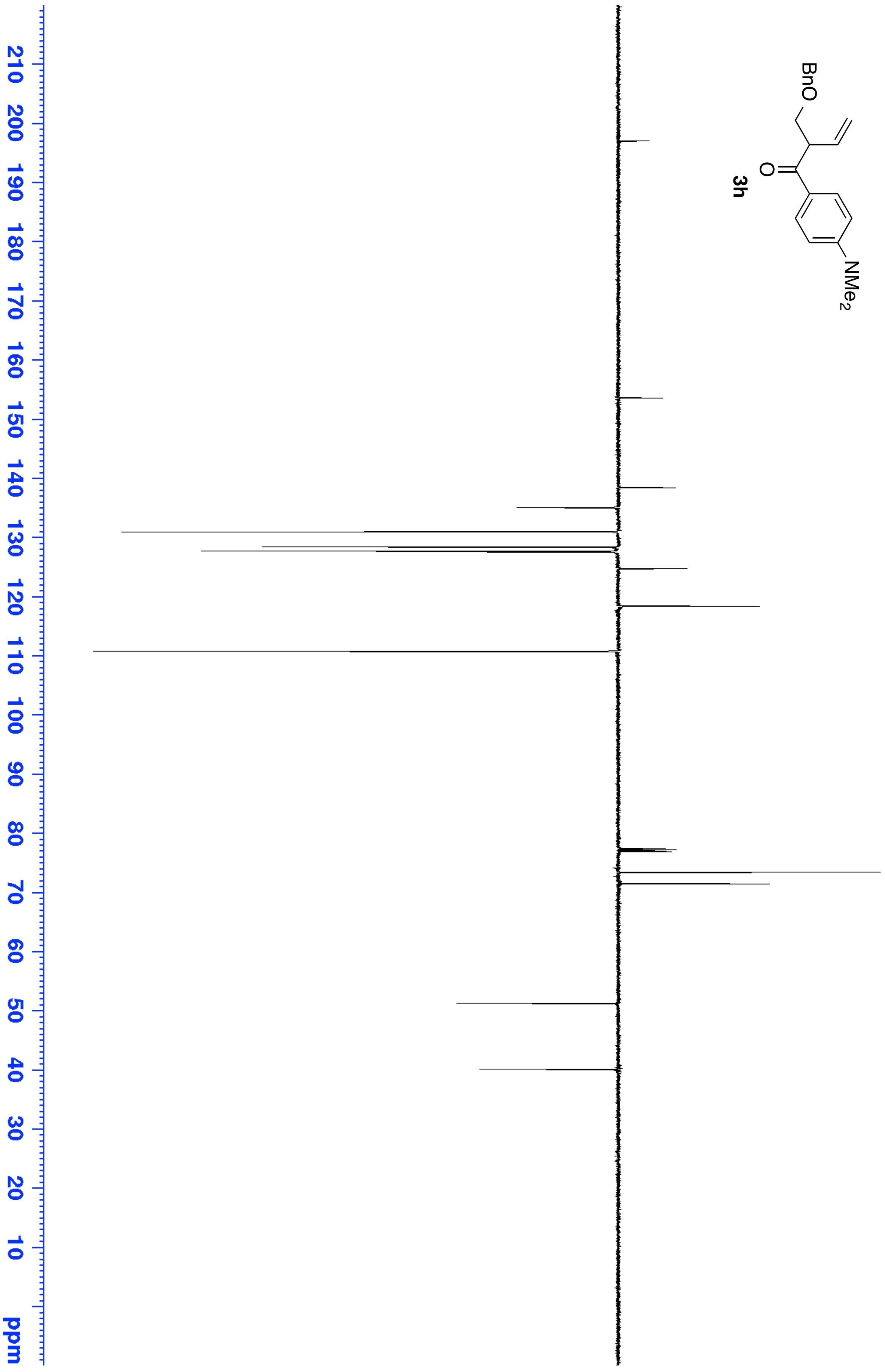
BnO
O
NMe2
3h
210 200 190 180 170 160 150 140 130 120 110 100 90 80 70 60 50 40 30 20 10 ppm

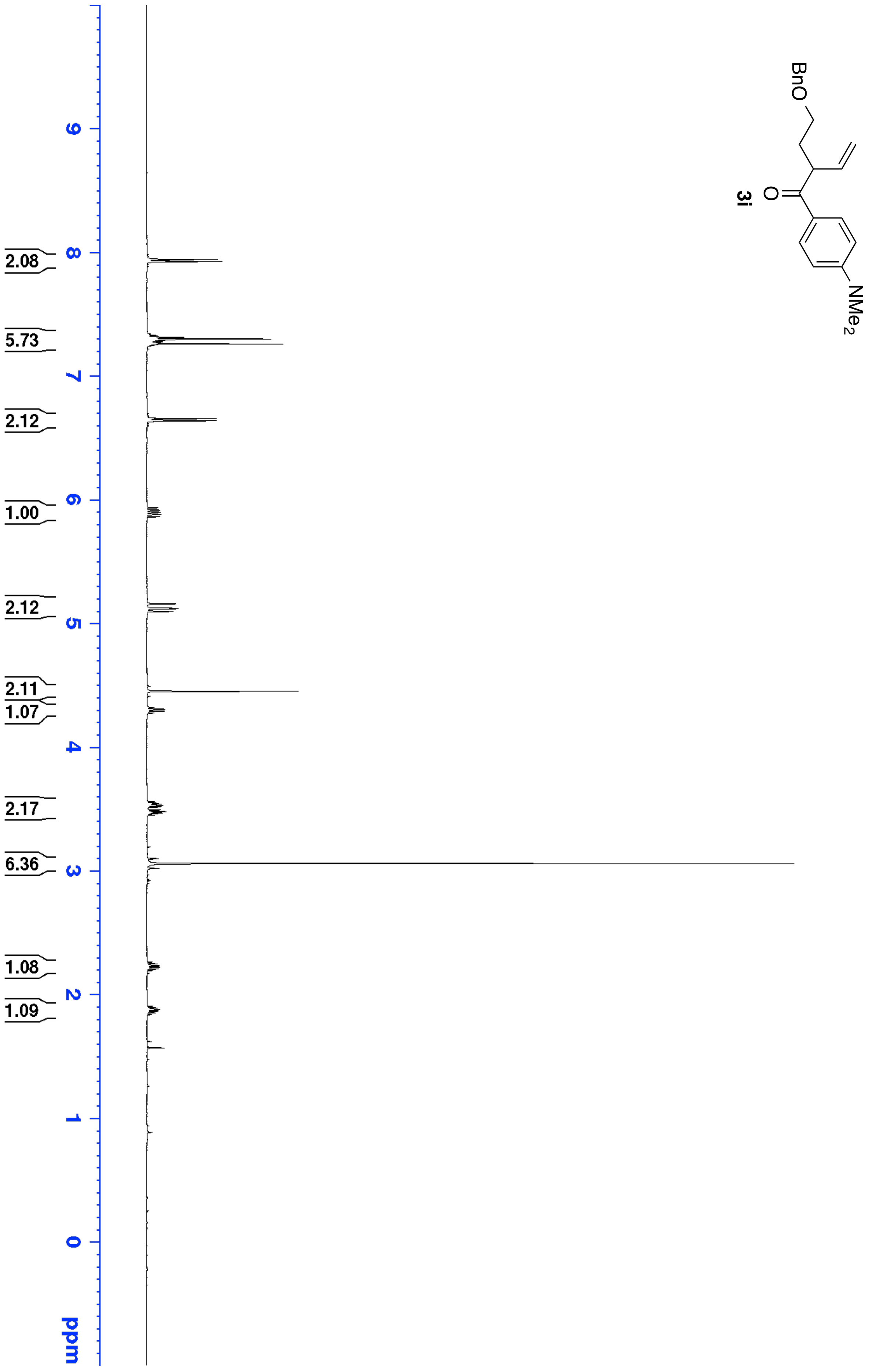
BnO
O
3i
NMe2
2.08
5.73
2.12
1.00
2.12
2.11
1.07
2.17
6.36
1.08
1.09
9
8
7
6
5
4
3
2
1
0
ppm

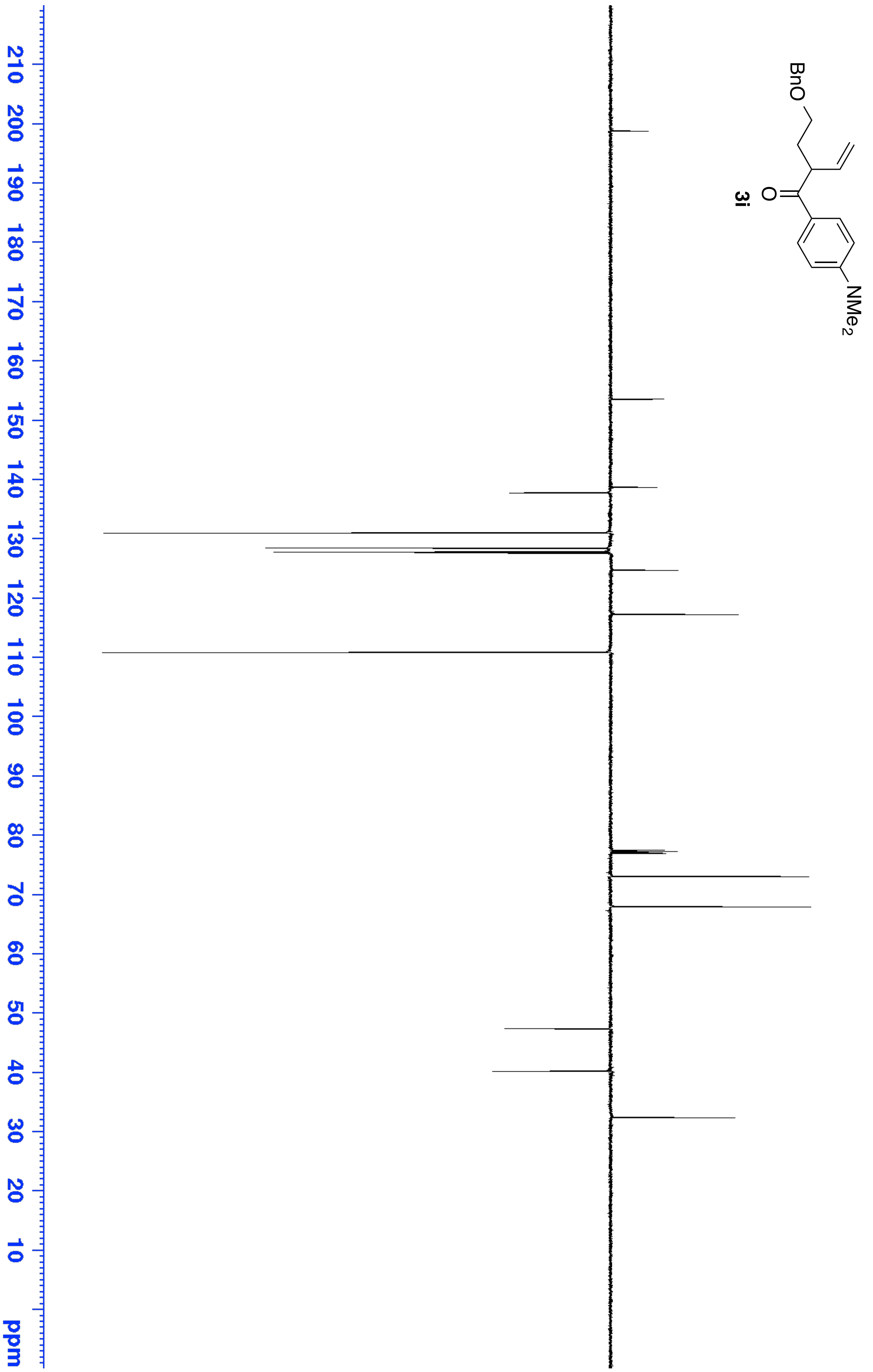
BnO
O
NMe2
3i
210 200 190 180 170 160 150 140 130 120 110 100 90 80 70 60 50 40 30 20 10 ppm

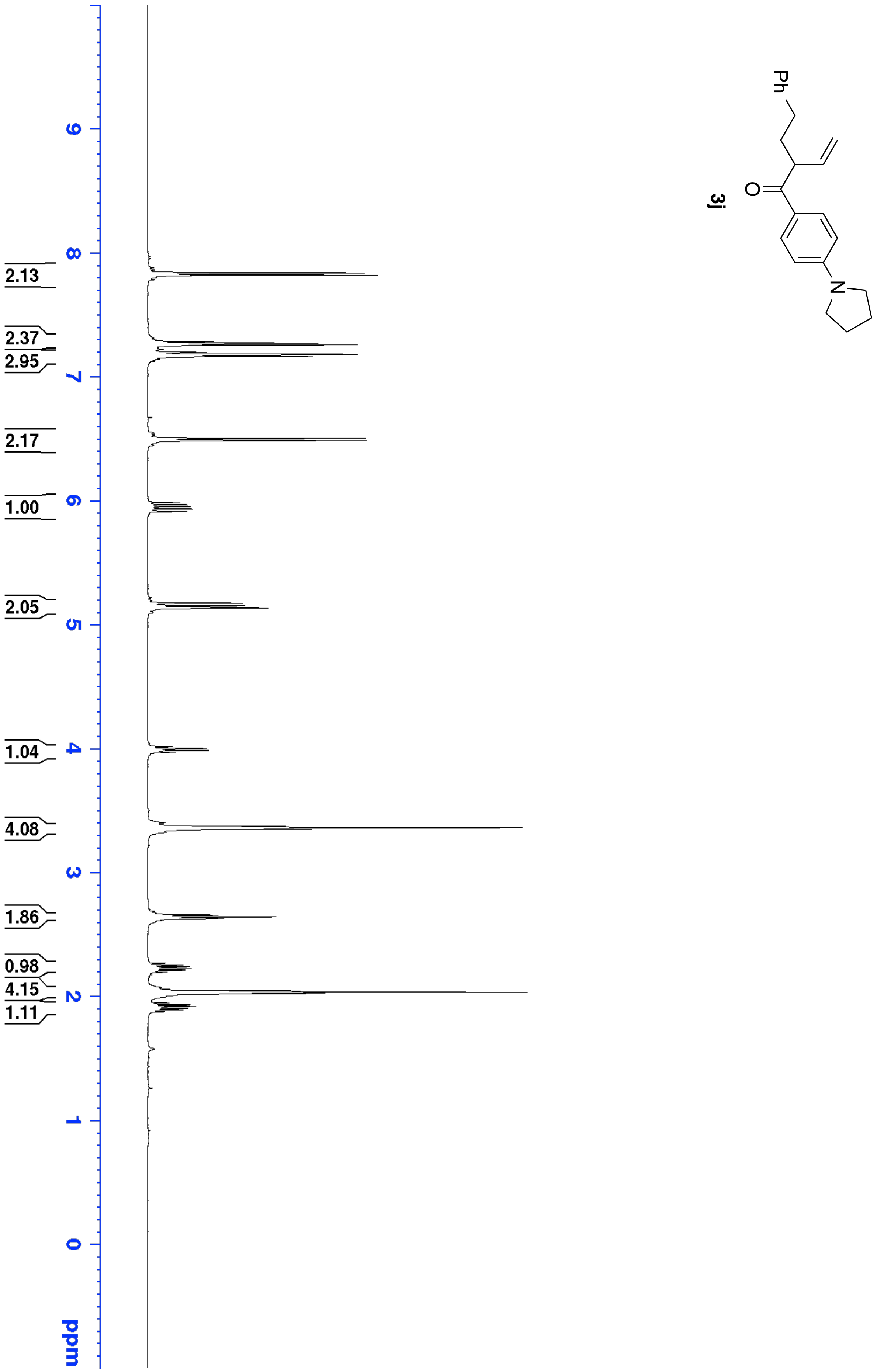
3j
Ph
O
N
2.13
2.37
2.95
2.17
1.00
2.05
1.04
4.08
1.86
0.98
4.15
1.11
9
8
7
6
5
4
3
2
1
0
ppm

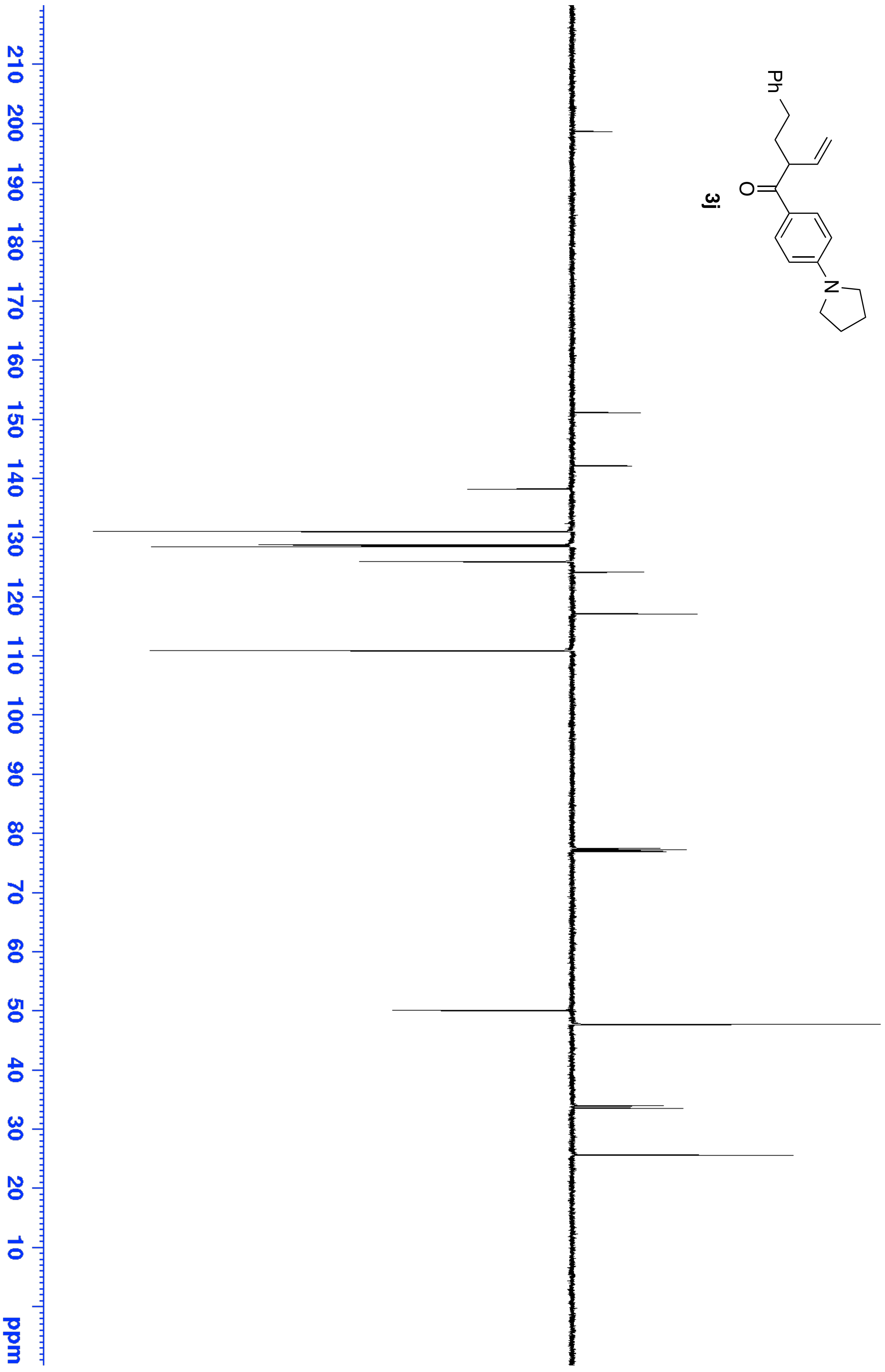
Ph
O
N
3j
210 200 190 180 170 160 150 140 130 120 110 100 90 80 70 60 50 40 30 20 10 ppm

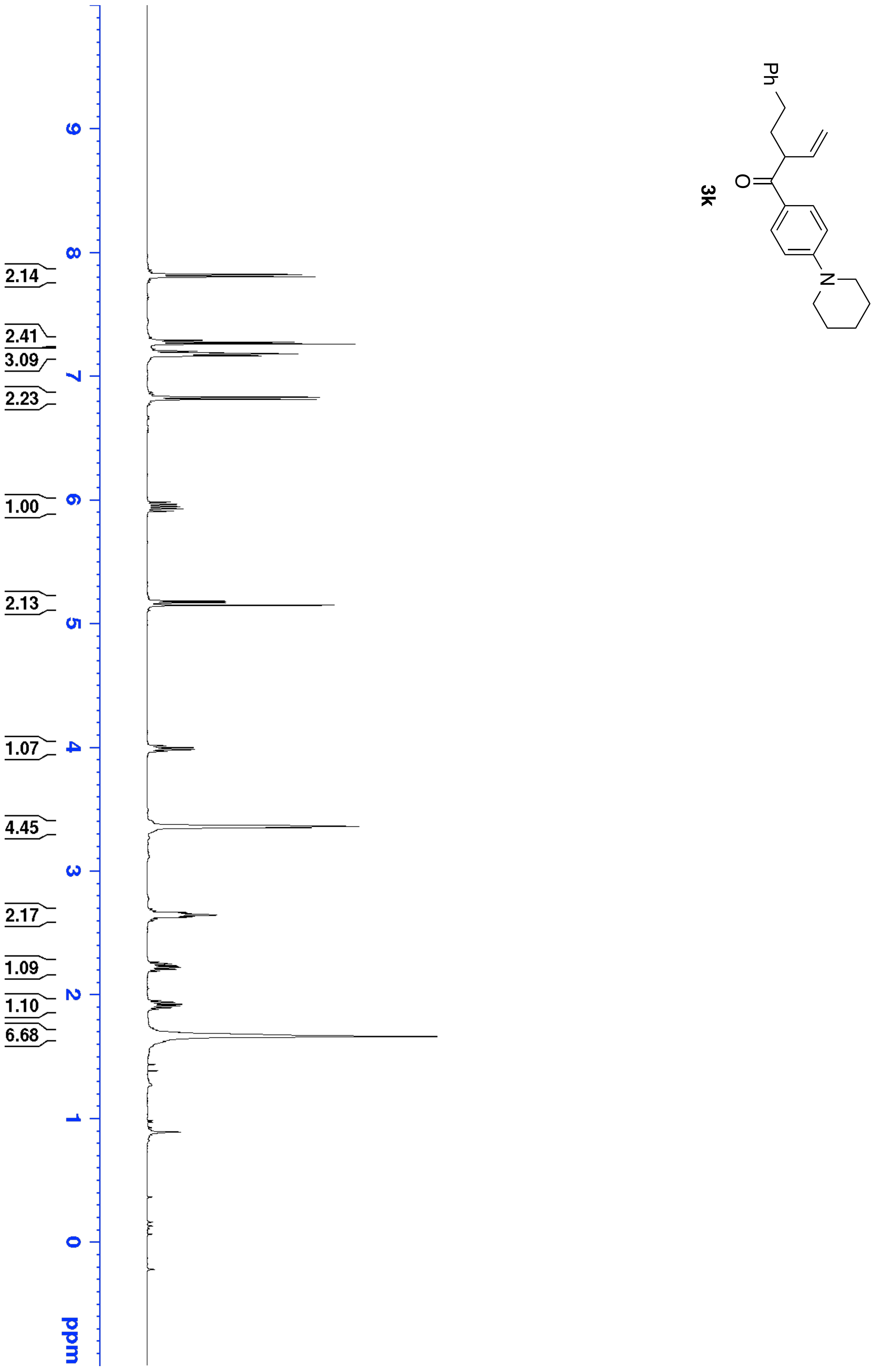
3k
Ph
O
N
ppm
2.14
2.41
3.09
2.23
1.00
2.13
1.07
4.45
2.17
1.09
1.10
6.68

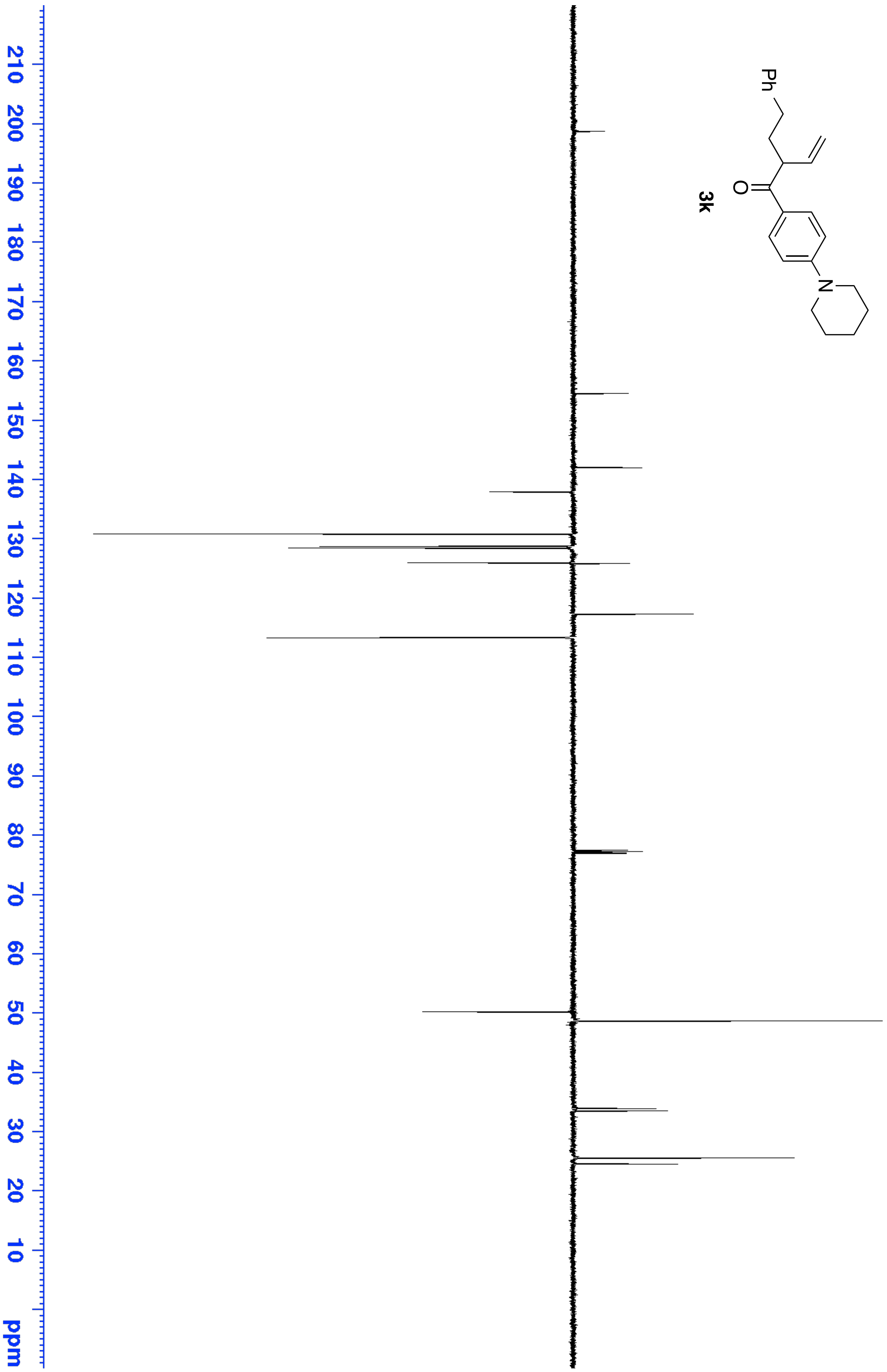
Ph
O
N
3k
210 200 190 180 170 160 150 140 130 120 110 100 90 80 70 60 50 40 30 20 10 ppm

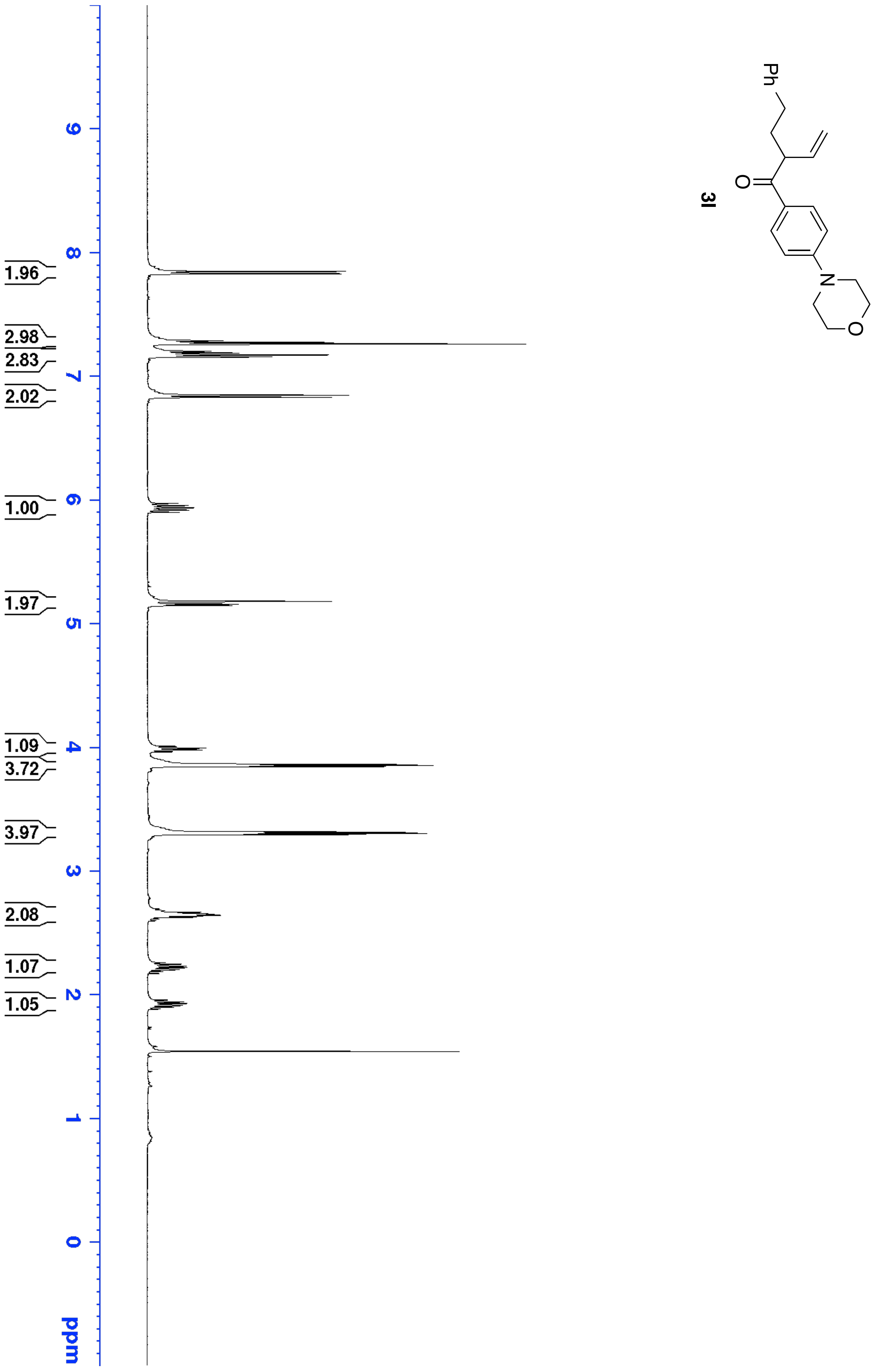
3l
Ph
O
N
ppm
9
8
7
6
5
4
3
2
1
0
1.96
2.98
2.83
2.02
1.00
1.97
1.09
3.72
3.97
2.08
1.07
1.05

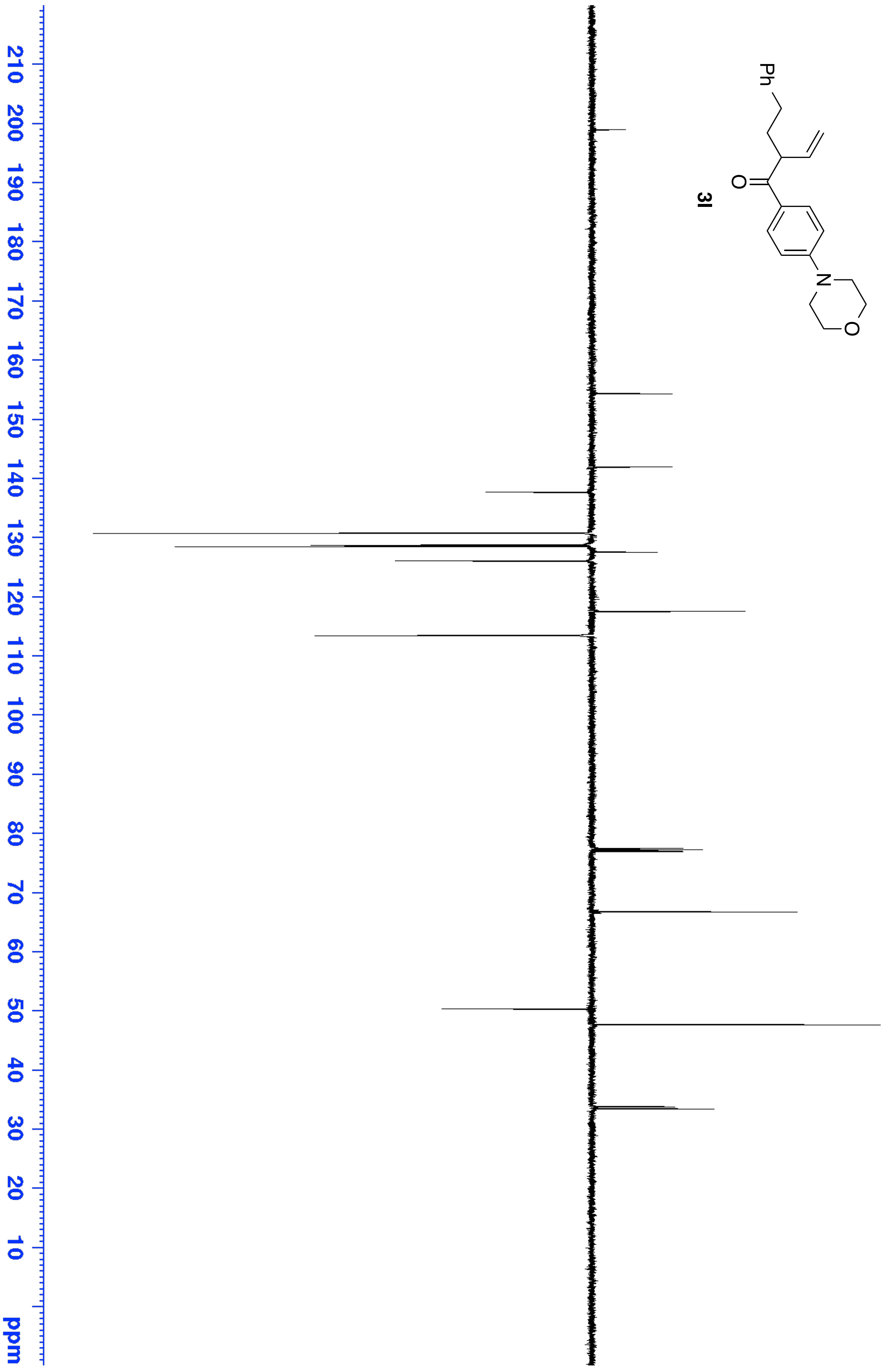
Ph
O
N
O
3l
210
200
190
180
170
160
150
140
130
120
110
100
90
80
70
60
50
40
30
20
10
ppm

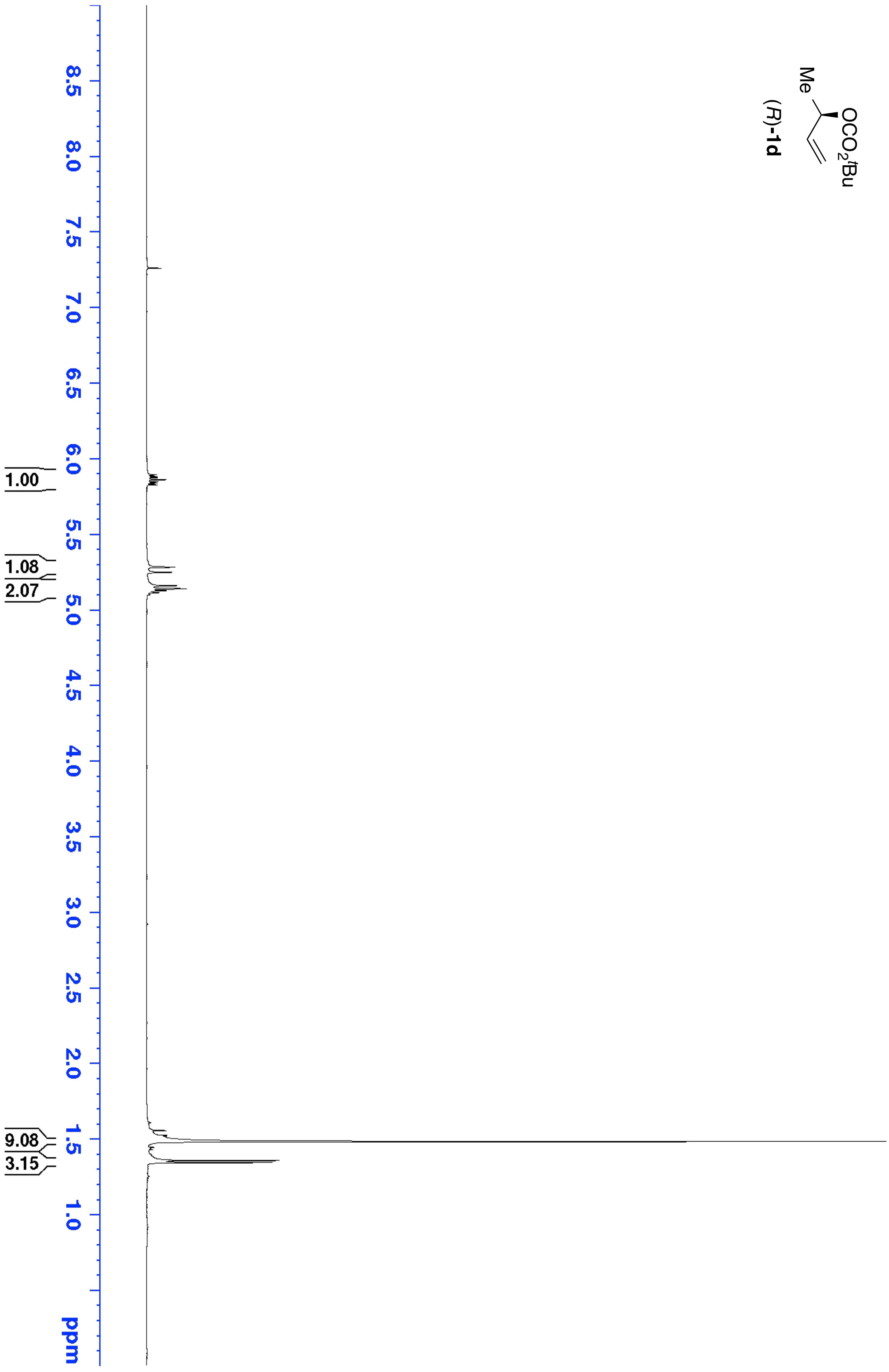

OCO2tBu
Me
(R)-1d
1.00
1.08
2.07
9.08
3.15
8.5
8.0
7.5
7.0
6.5
6.0
5.5
5.0
4.5
4.0
3.5
3.0
2.5
2.0
1.5
1.0
ppm

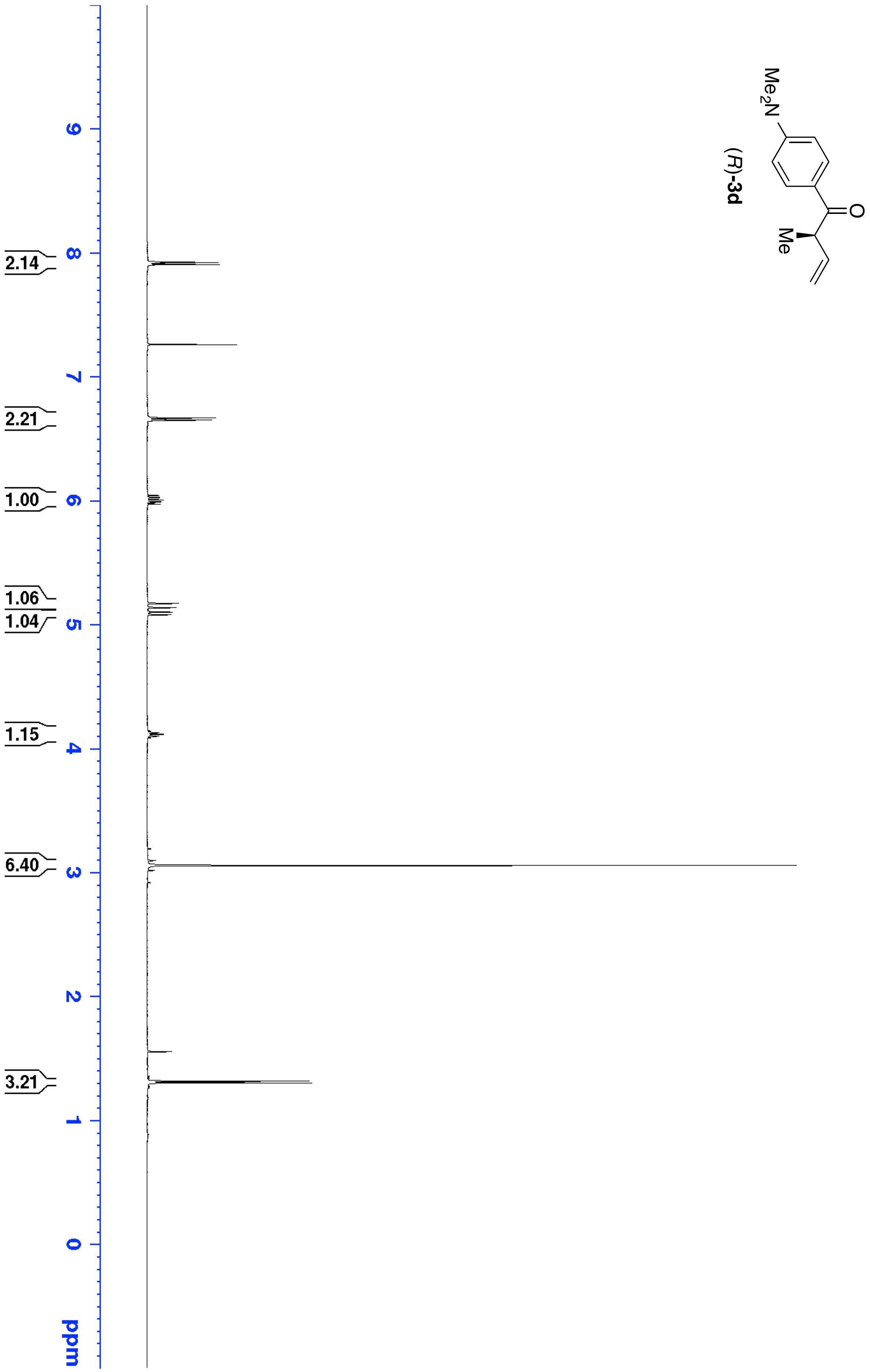
Me2N
O
Me
(R)-3d
2.14
2.21
1.00
1.06
1.04
1.15
6.40
3.21
9
8
7
6
5
4
3
2
1
0
ppm

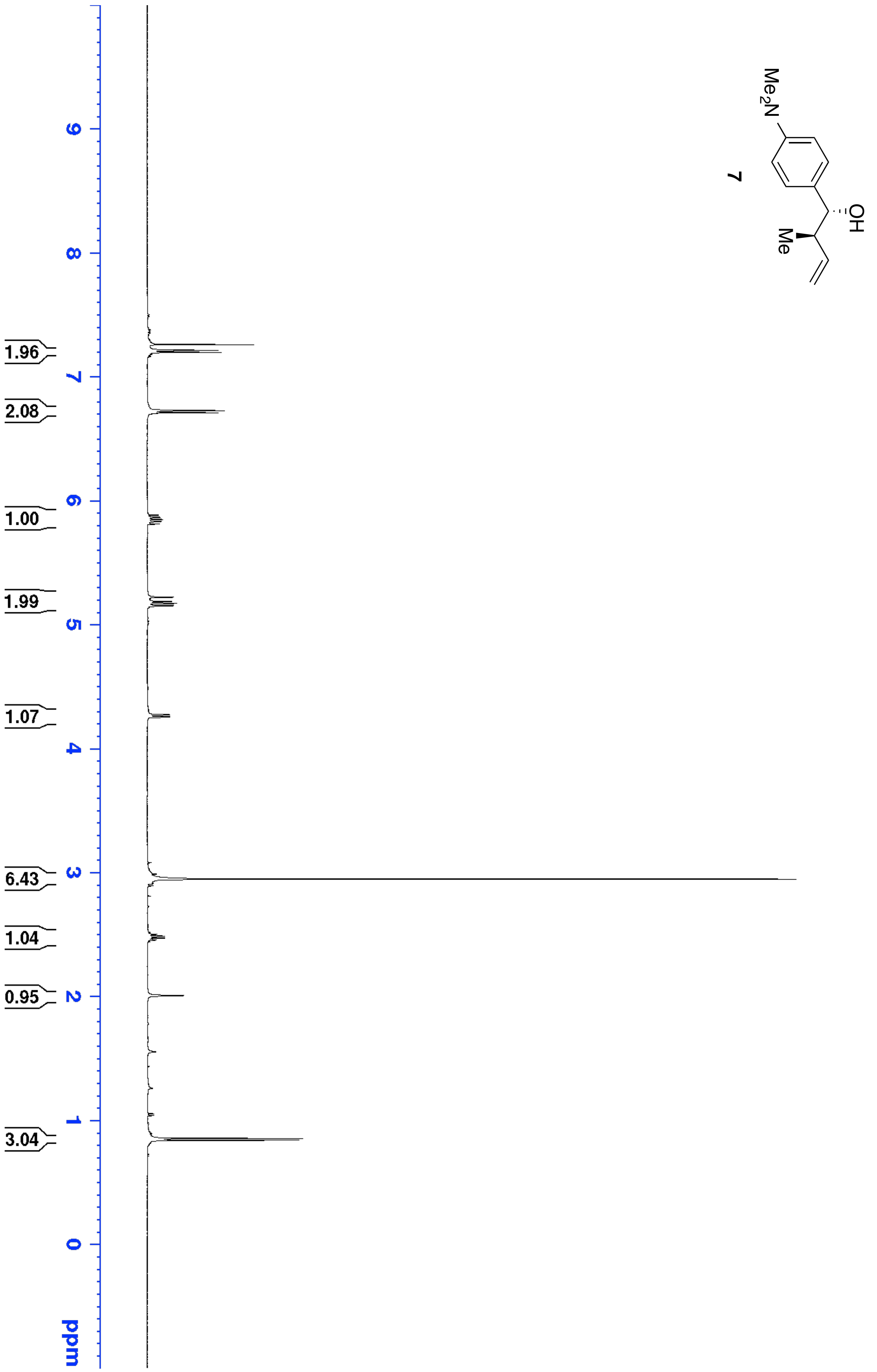
7
Me2N
OH
Me
1.96
2.08
1.00
1.99
1.07
6.43
1.04
0.95
3.04
9
8
7
6
5
4
3
2
1
0
ppm

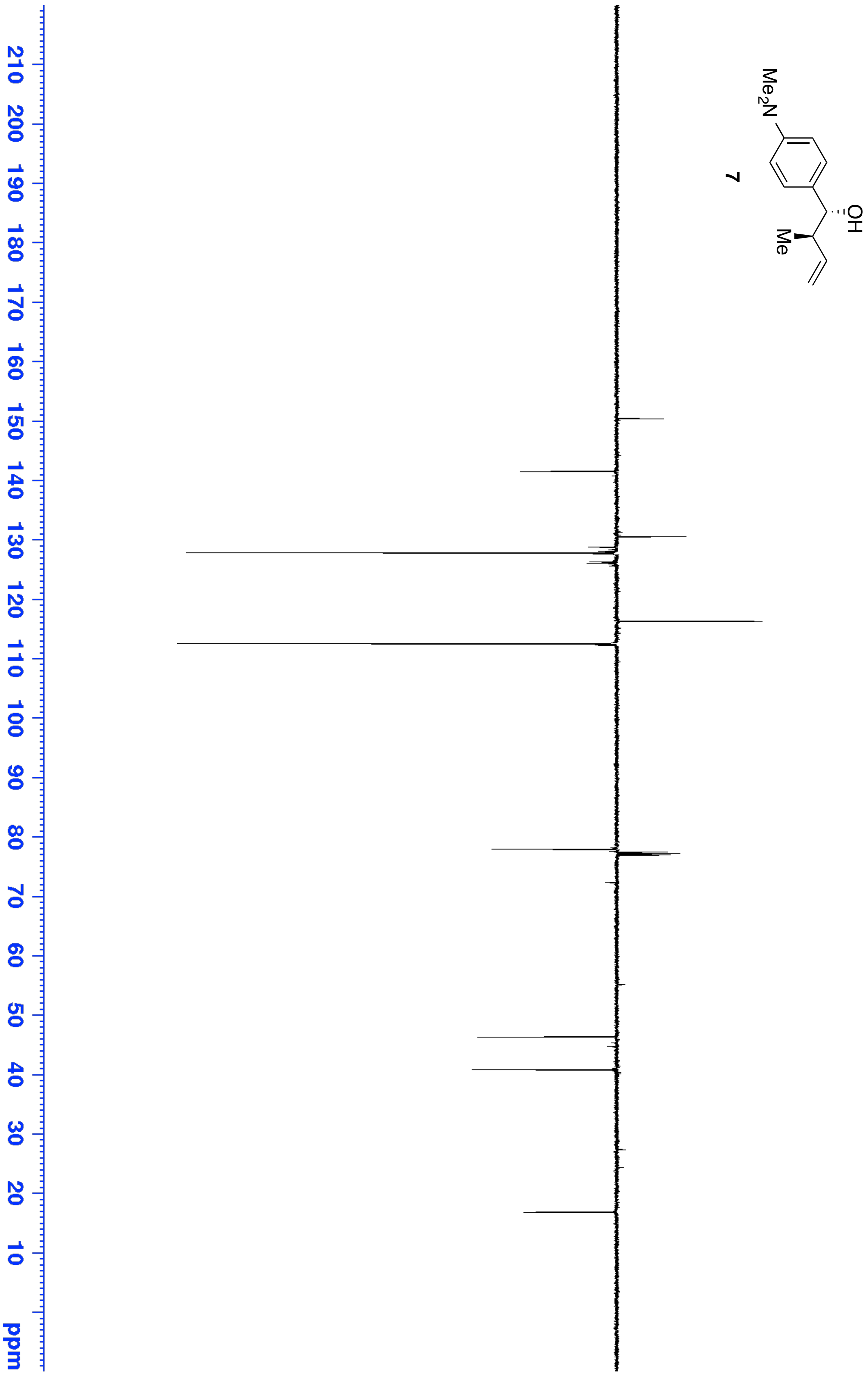
OH
Me
Me_2N
7
210 200 190 180 170 160 150 140 130 120 110 100 90 80 70 60 50 40 30 20 10 ppm

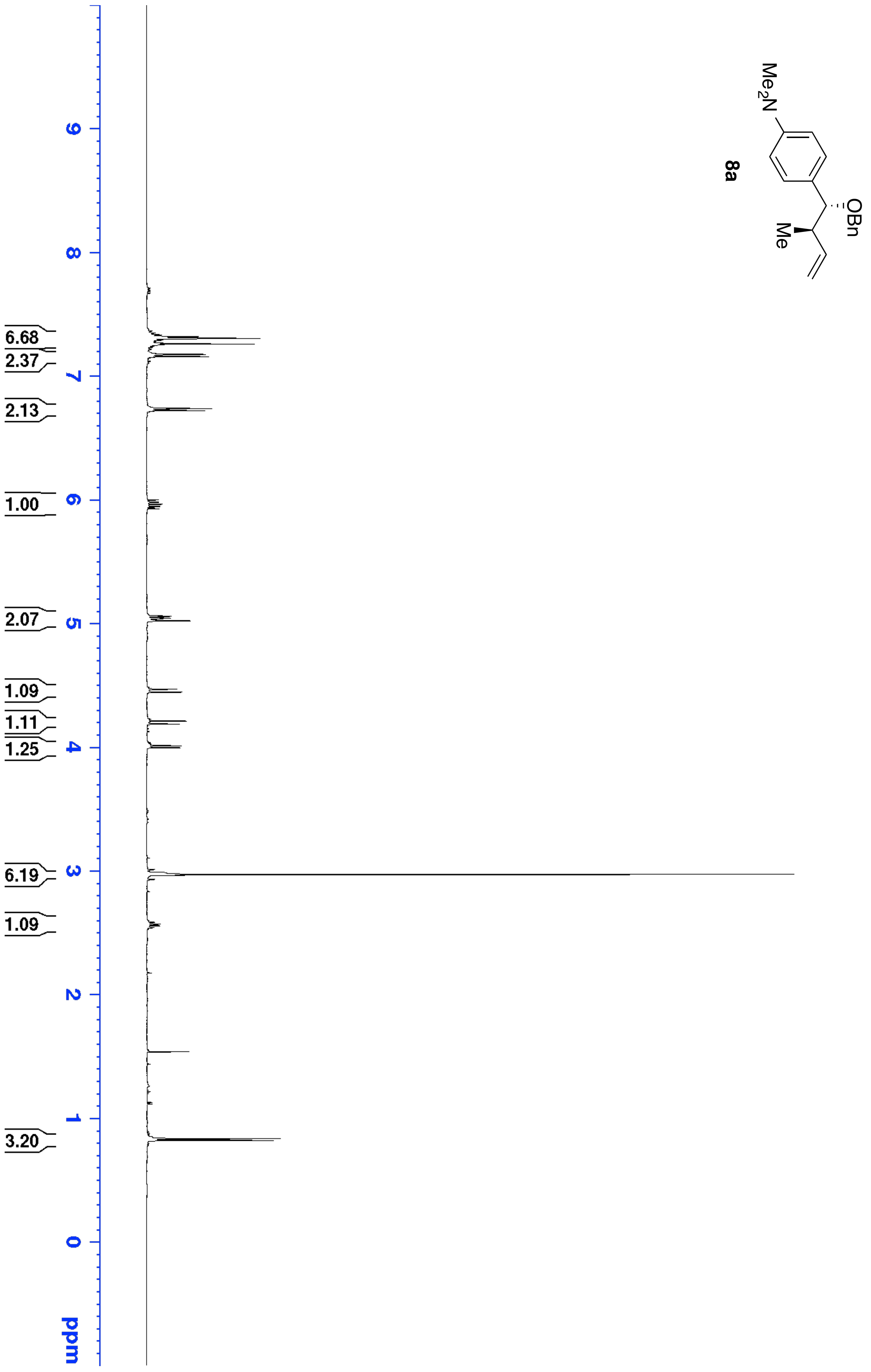
Me$_2$N
OBn
Me
8a
6.68
2.37
2.13
1.00
2.07
1.09
1.11
1.25
6.19
1.09
3.20
9
8
7
6
5
4
3
2
1
0
ppm

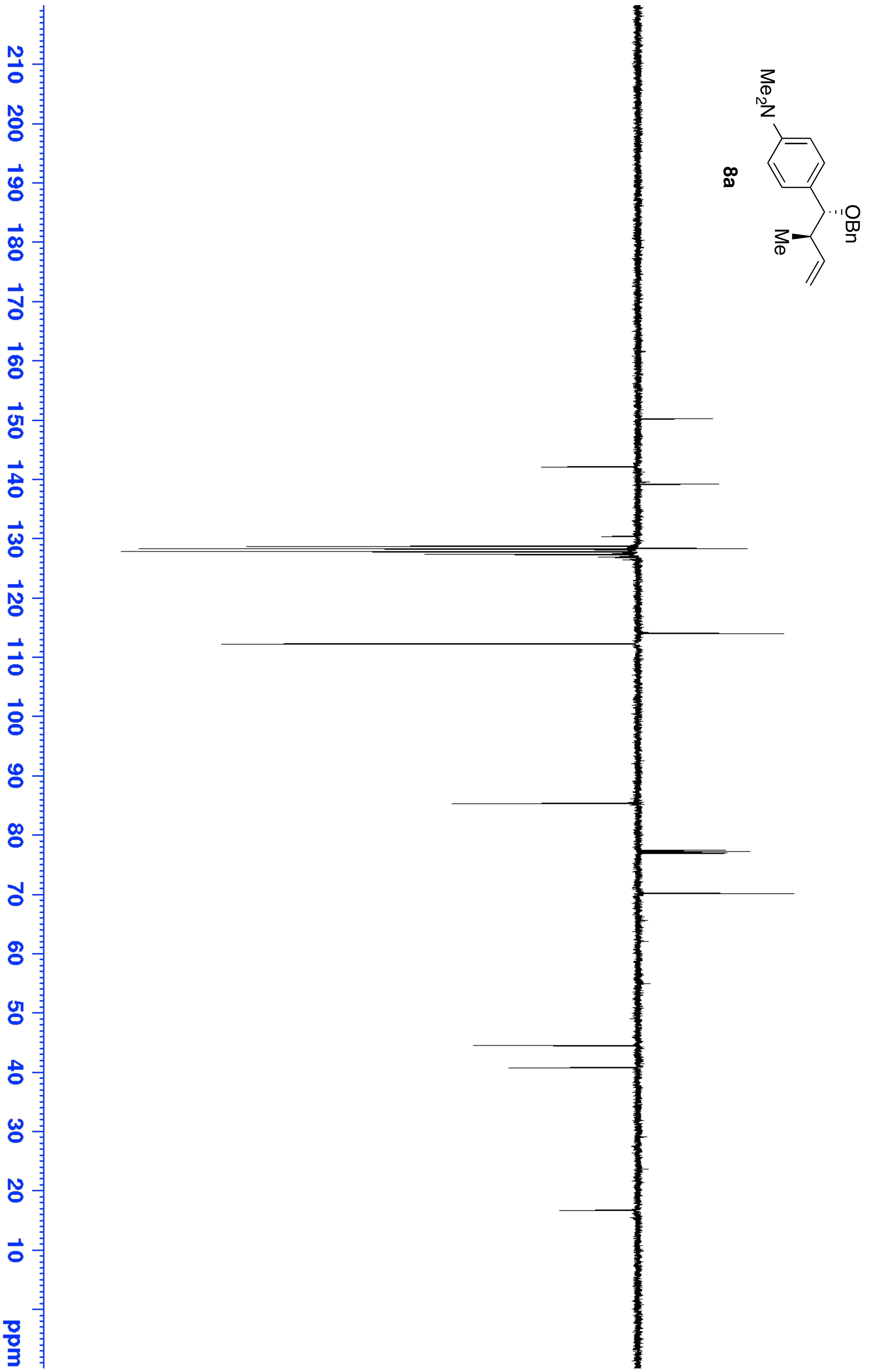
OBn
Me
Me_2N
8a
ppm

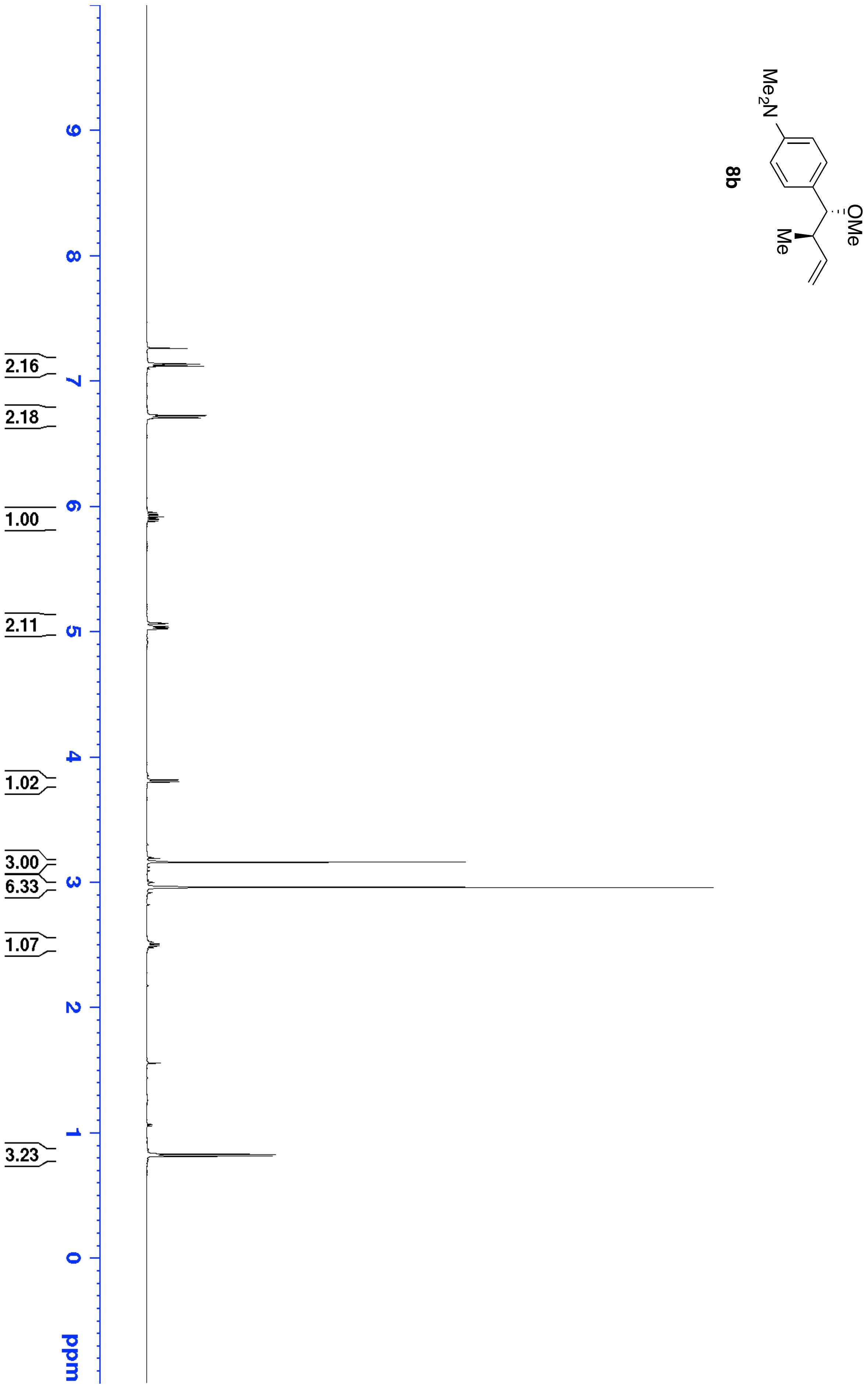
Me$_2$N
OMe
Me
8b
2.16
2.18
1.00
2.11
1.02
3.00
6.33
1.07
3.23
9
8
7
6
5
4
3
2
1
0
ppm

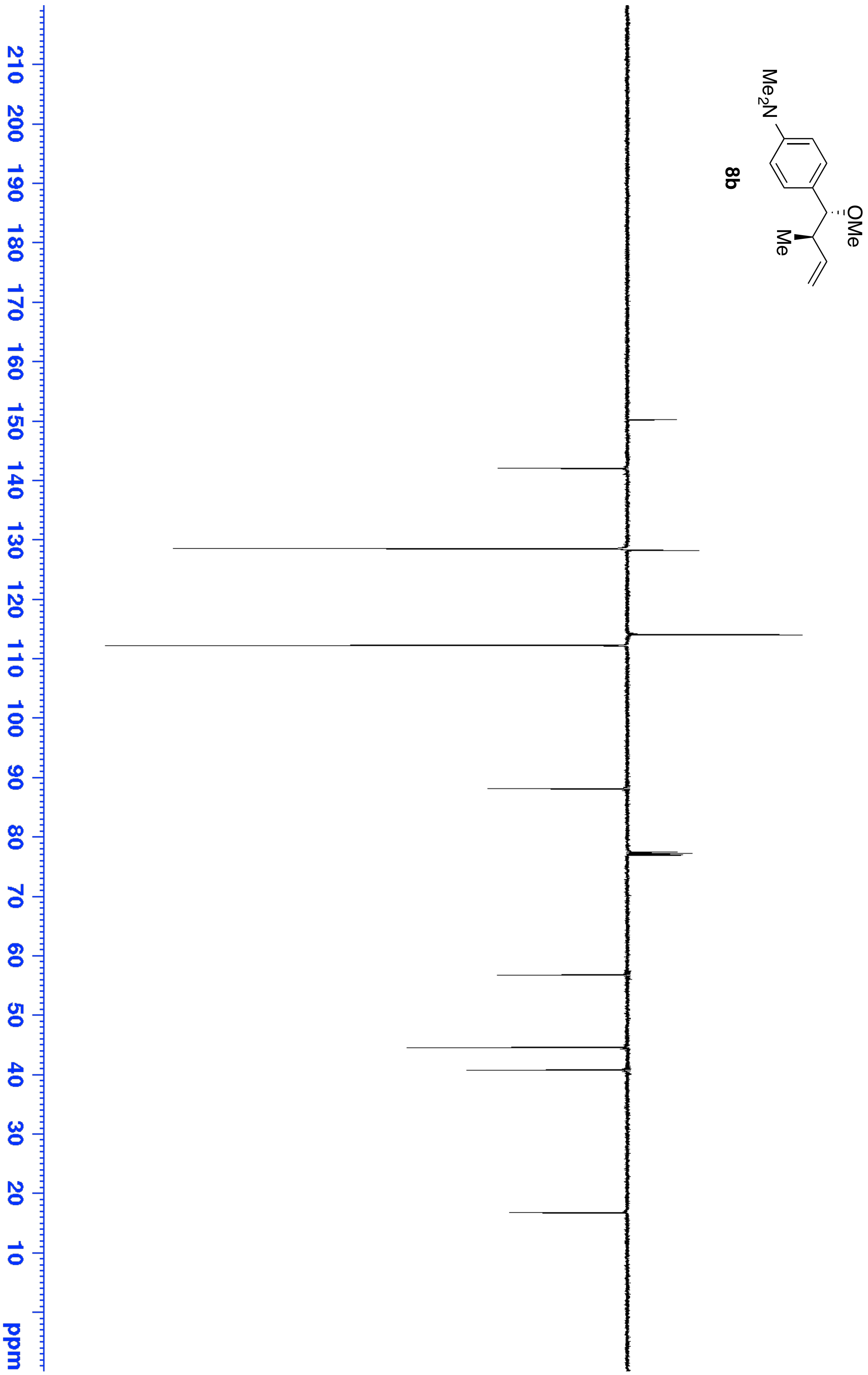

Me2N
OMe
Me
8b
210 200 190 180 170 160 150 140 130 120 110 100 90 80 70 60 50 40 30 20 10 ppm

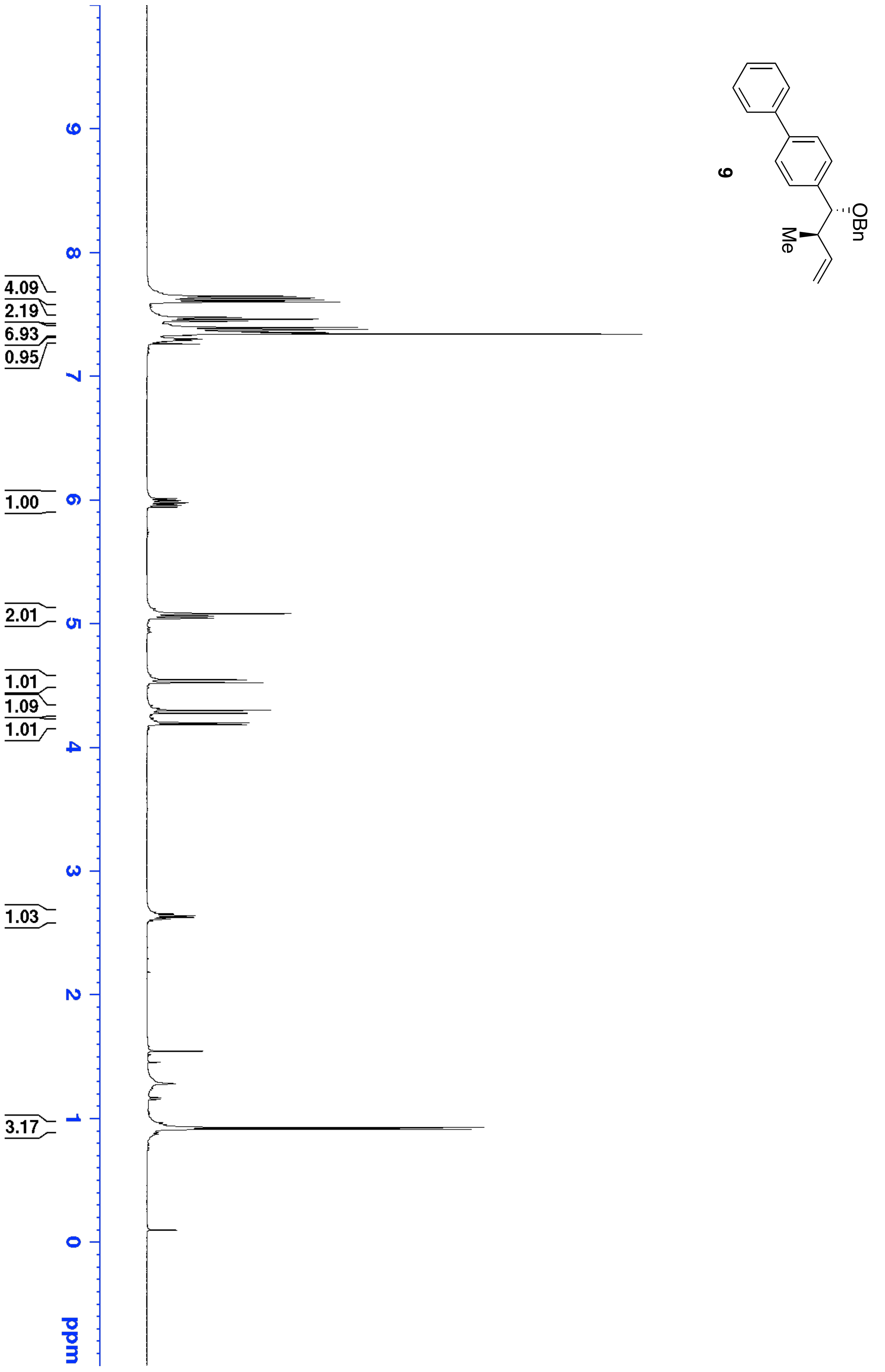

9
OBn
Me
4.09
2.19
6.93
0.95
1.00
2.01
1.01
1.09
1.01
1.03
3.17
9
8
7
6
5
4
3
2
1
0
ppm

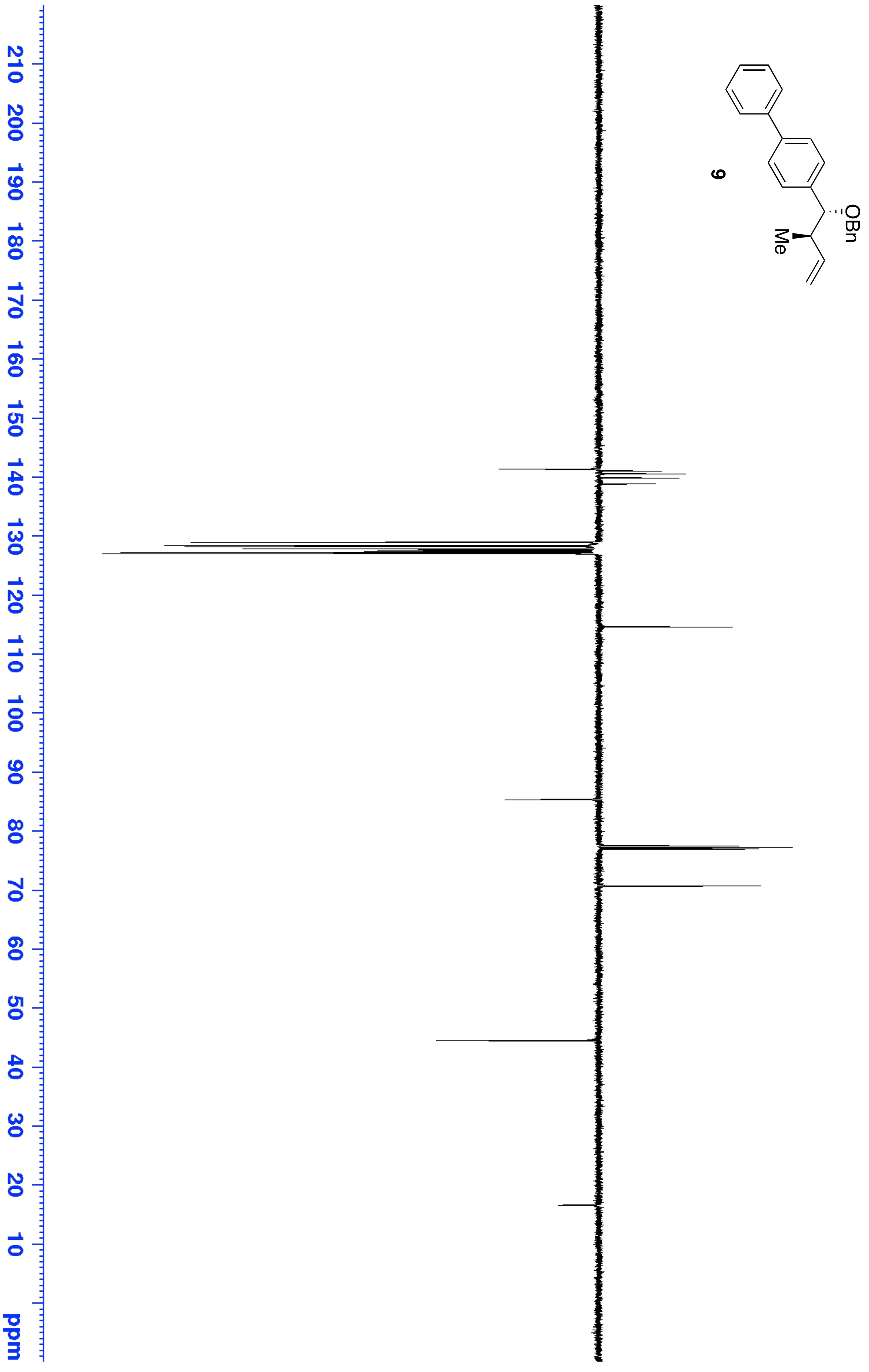
OBn
Me
9
210 200 190 180 170 160 150 140 130 120 110 100 90 80 70 60 50 40 30 20 10 ppm

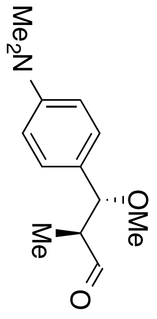

10

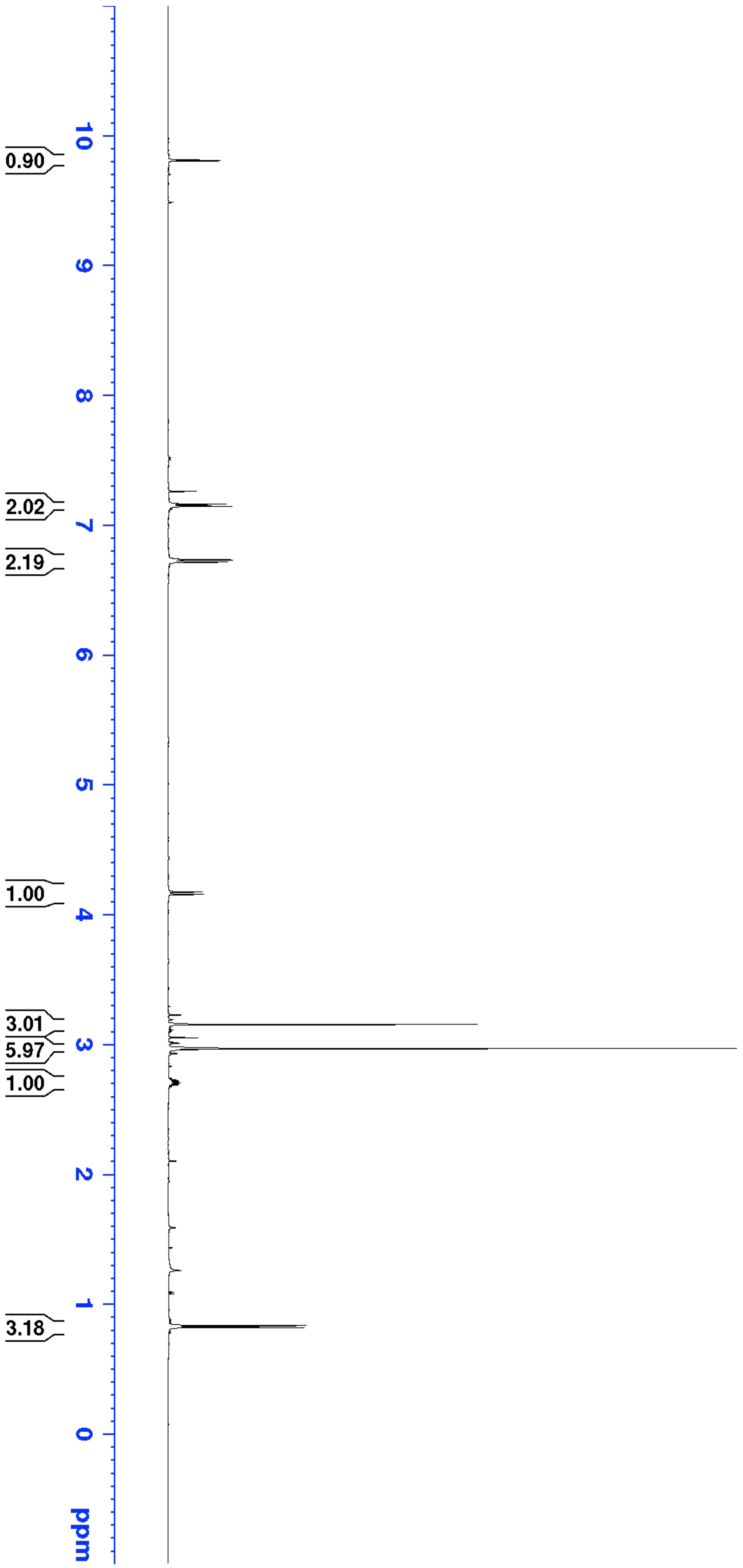

12. HPLC Chromatograms

(*R*)-1-(4-(Dimethylamino)phenyl)-2-methylbut-3-en-1-one ((*R*)-3d)

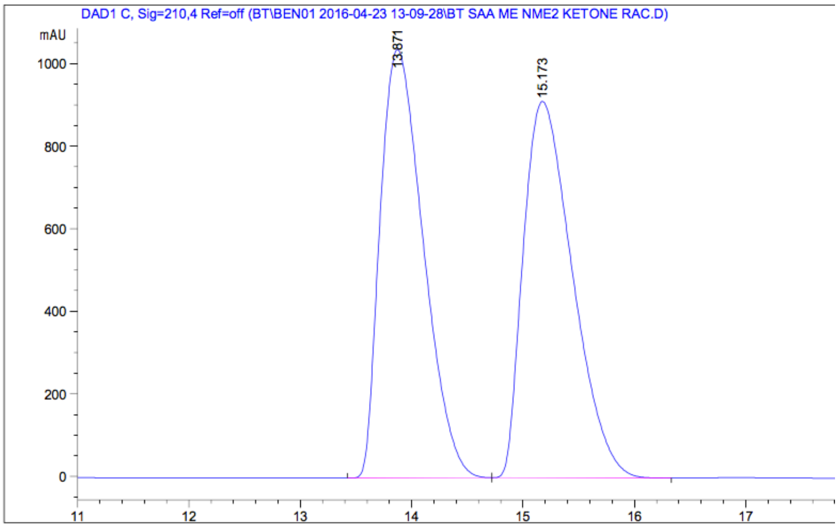

Peak #	RetTime [min]	Type	Width [min]	Area [mAU*s]	Height [mAU]	Area %
1	13.871	BB	0.4270	2.78423e4	1037.69739	49.9042
2	15.173	BB	0.4878	2.79492e4	911.43732	50.0958

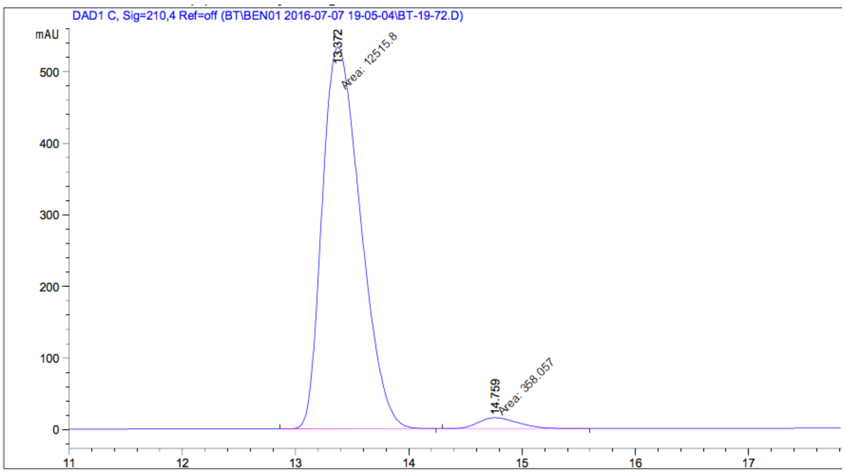

Peak #	RetTime [min]	Type	Width [min]	Area [mAU*s]	Height [mAU]	Area %
1	13.372	MM	0.3917	1.25158e4	532.60858	97.2187
2	14.759	MM	0.3979	358.05652	14.99719	2.7813
